# Supplementary material for: Reprogramming of bacterial virulence by lysine acetylation
Source: Nat Commun. 2026 Apr 27;17:3859. doi: 10.1038/s41467-026-72244-8 (PMC13125535; doi:10.1038/s41467-026-72244-8)
Supplement: Supplementary file 1 — Supplementary Information [file 41467_2026_72244_MOESM1_ESM.pdf]

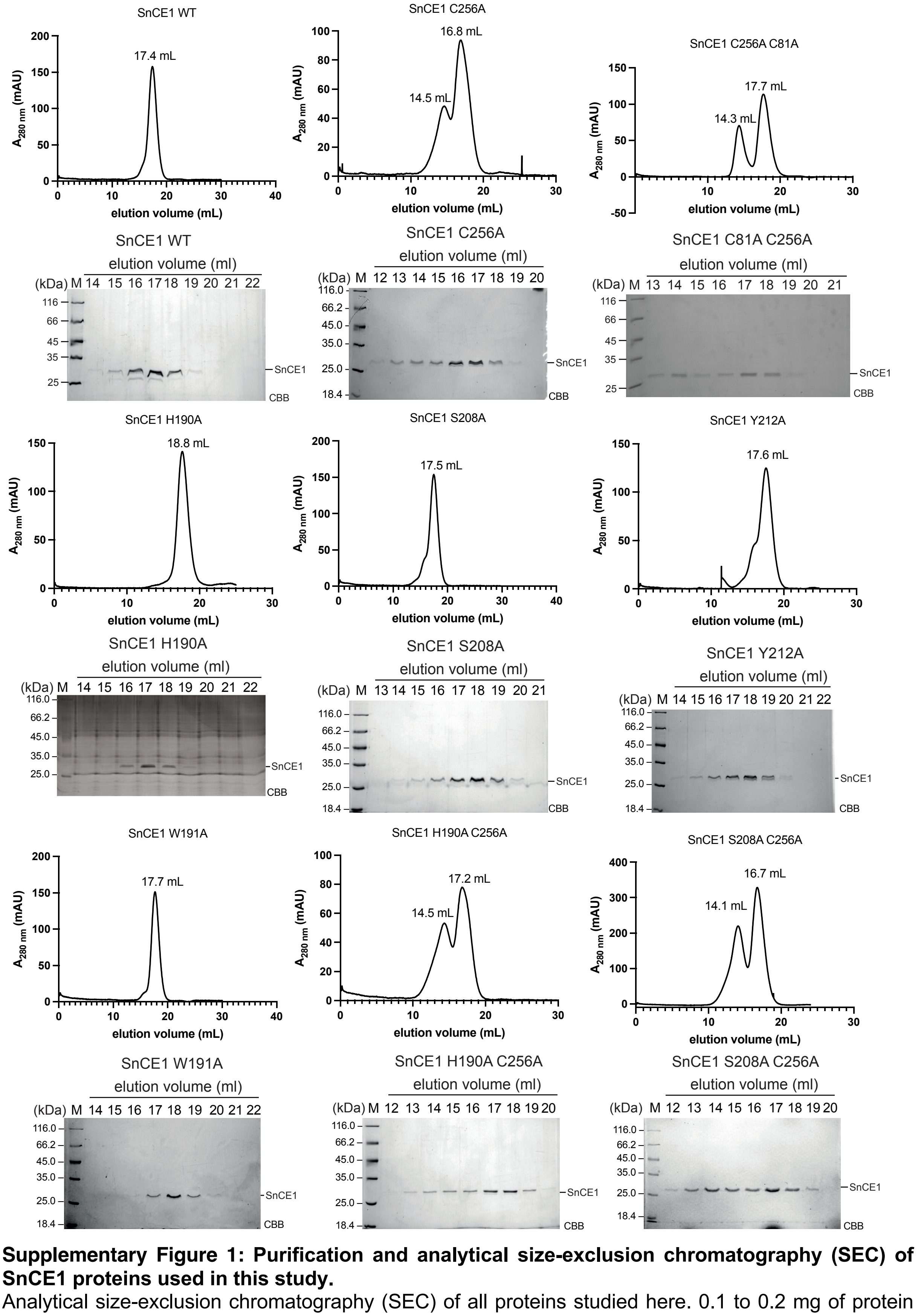

Supplementary Figure 1: Purification and analytical size-exclusion chromatography (SEC) of SnCE1 proteins used in this study.

Analytical size-exclusion chromatography (SEC) of all proteins studied here. 0.1 to 0.2 mg of protein was analysed on a Superdex 200 Increase 10/300 GL column. Below the SEC elution profiles showing the absorption at 280 nm ($A_{280\ nm}$) in mAU (milli absorbance units) the SDS-PAGE gels were shown analysing fractions of the observed absorption peaks. SDS-PAGE gel was stained using Coomassie brilliant blue (CBB). Source data are provided as Source Data file.

a

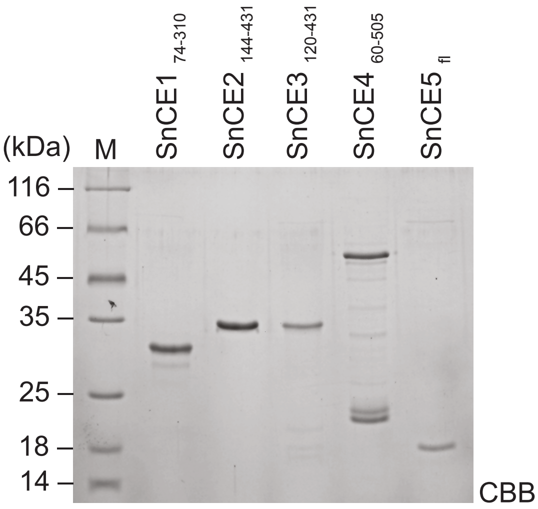

b

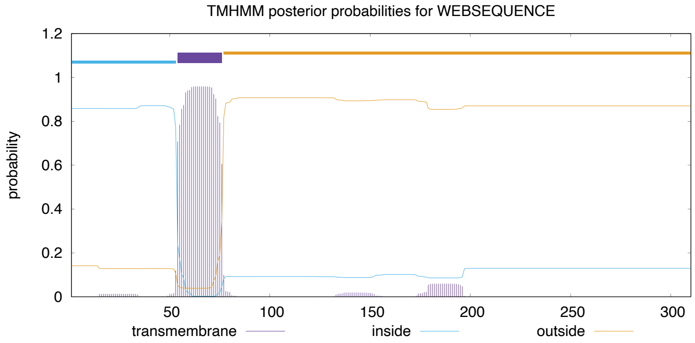

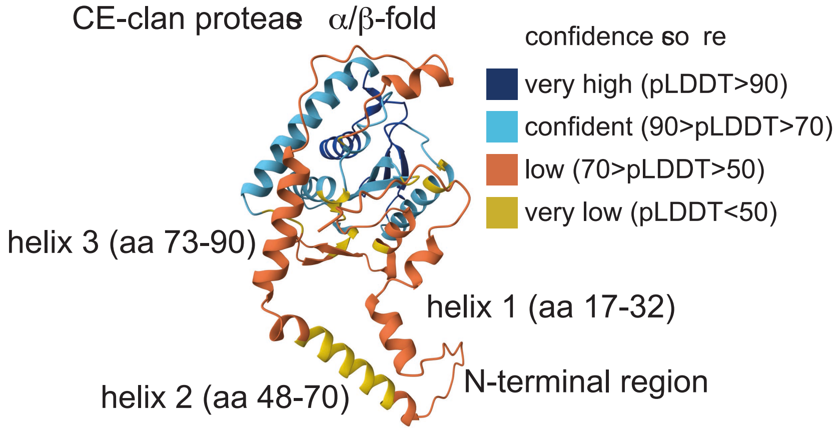

c

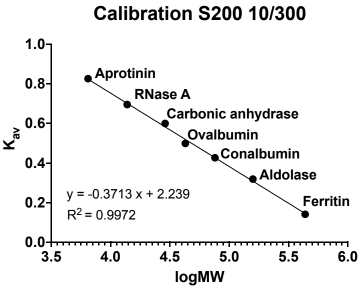

		peak 1			peak 2		
protein	exp. MW [Da]	elution volume [mL]	calc. MW [Da]	oligomeric state [calc. MW/exp. MW]	elution volume [mL]	calc. MW [Da]	oligomeric state [calc. MW/exp. MW]
SnCE1$_{74\text{-}310}$	28354				17.4	36178	1.3
SnCE1$_{104\text{-}310}$	25079				18.5	22260	0.9
SnCE1$_{104\text{-}310}$ C256A	25047	15.0	99715	4.0	18.4	24034	1.0
SnCE1$_{74\text{-}310}$ C256A	28322	14.5	124443	4.3	16.8	46714	1.7
SnCE1$_{74\text{-}310}$ Y212A	28262				17.6	33223	1.2
SnCE1$_{74\text{-}310}$ S208A	28338				17.5	34669	1.2
SnCE1$_{74\text{-}310}$ C81A/C256A	28290	14.3	135510	4.8	17.7	31837	1.1
SnCE1$_{74\text{-}310}$ S208A/C256A	28306	14.3	124443	4.8	17.0	42899	1.5
SnCE1$_{74\text{-}310}$ H190A	28288				18.8	19926	0.7
SnCE1$_{74\text{-}310}$ H190A/C256A	28256	14.5	124443	4.3	17.2	39395	1.4
SnCE1$_{74\text{-}310}$ W191A	28239				17.7	31837	1.1
SnCE1$_{74\text{-}310}$ C256A AcK78	28364	14.4	129858	4.6	17.8	30510	1.1
SnCE1$_{74\text{-}310}$ C256A AcK94	28364	14.3	135510	4.8	17.8	30510	1.1
SnCE1$_{74\text{-}310}$ C256A AcK98	28364	14.4	129858	4.6	17.9	29237	1.0
SnCE1$_{74\text{-}310}$ C256A AcK103	28364	14.2	141407	5.0	17.6	33223	1.2
SnCE1$_{74\text{-}310}$ C256A AcK106	28364	14.3	135510	4.8	17.9	29237	1.0
SnCE1$_{74\text{-}310}$ C256A AcK209	28364	14.4	129858	4.6	17.7	31837	1.1
SnCE1$_{74\text{-}310}$ Y212A AcK231	28304				18.3	24656	0.9
SnCE1$_{74\text{-}310}$ C256A AcK231	28364				17.6	33223	1.2
SnCE1$_{74\text{-}310}$ C256A AcK248	28364	14.2	141407	5.0	17.5	34669	1.2
SnCE1$_{74\text{-}310}$ C256A S/T/CtoA	28164	14.0	153983	5.5	17.4	36178	1.3
SnCobB (S200 10/300)	30524	13.7	174975	5.7	17.7	31837	1.0

Supplementary Figure 2: Final purity obtained for SnCE1-5 and determination of oligomeric states by analytical SEC of proteins expressed and purified in this study.

a. SDS-PAGE gel showing the final purity obtained for *Simkania negevensis* SnCE1-5. 1 µg of recombinantly expressed and purified protein was loaded on the SDS-PAGE gel. Staining was done with Coomassie brilliant blue (CBB).

b. Prediction of a potential N-terminal transmembrane helices in SnCE1. The prediction of transmembrane helices in SnCE1 was done with TMHMM2.0 (https://services.healthtech.dtu.dk/services/TMHMM-2.0/). It predicts the presence of a transmembrane helix with high probability covering residues 54 to 74. This agrees in part with the AlphaFold3 model obtained for full length SnCE1 (https://alphafoldserver.com/) showing the presence of α-helices in the N-terminal part covering residues 17-32 with very low confidence (pLDDT<50), residues 48-70 with low confidence (50>pLDDT>70) and residues 73-90 again with very low confidence (pLDDT<50)[1-3].

c. The elution volumes and calculated molecular weights (calc. MW) were used in comparison to expected molecular weights (exp. MW) to calculate the apparent oligomeric state of the proteins analysed in this study. The calculated molecular weights were obtained based on the elution volume using a calibration curve. Source data are provided as Source Data file.

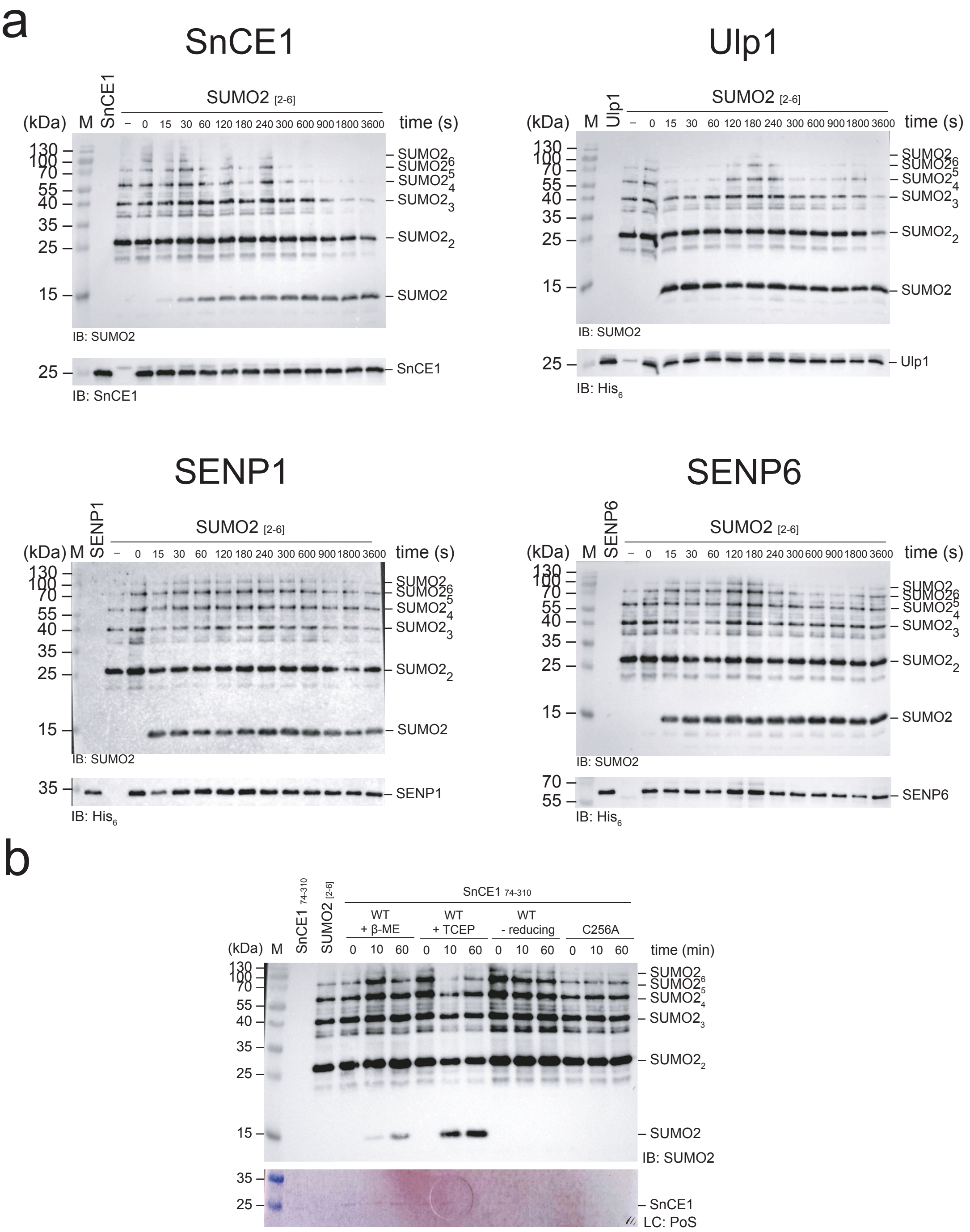

Supplementary Fig. 3: SnCE1 cleaves polymeric SUMO2$_{[2\text{-}6]}$-chains less efficiently compared to well-characterised deSUMOylases and its activity requires reducing conditions.

a. To compare the catalytic efficiency of SnCE1 to cleave SUMO2-chains, we produced recombinant human SENP1, SENP6 and yeast Ulp1 and compared their activities to cleave polymeric SUMO2$_{[2\text{-}6]}$-chains yielding mono-SUMO2. SnCE1 is an efficient deSUMOylase, however, less efficient compared to human SENP1, human SENP6 or yeast Ulp1. Shown are immunoblots using an anti-SUMO2 antibody to follow SUMO2$_{[2\text{-}6]}$-chain cleavage (IB: SUMO2). As loading controls, SnCE1 was stained with an anti-SnCE1 antibody (IB: SnCE1) and SENP1, SENP6 and Ulp1 with anti-His$_6$ antibody (IB: His$_6$). The experiment was performed in two independent technical replicates (*n*=2), one example is shown. Source data are provided as Source Data file.

b. SnCE1 deSUMOylase activity was analysed in presence of β-mercaptoethanol, TCEP and in absence of reducing agent. Presence of a reducing agent is essential for its catalytic activity, while it is inactive without. Shown is an immunoblot using an anti-SUMO2 antibody to follow SUMO2$_{[2\text{-}6]}$-chain cleavage (IB: SUMO2). As loading controls, the membranes were stained with Ponceau S-red solution (LC: PoS). The experiment was performed in two independent technical replicates (*n*=2), one example is shown. Source data are provided as Source Data file.

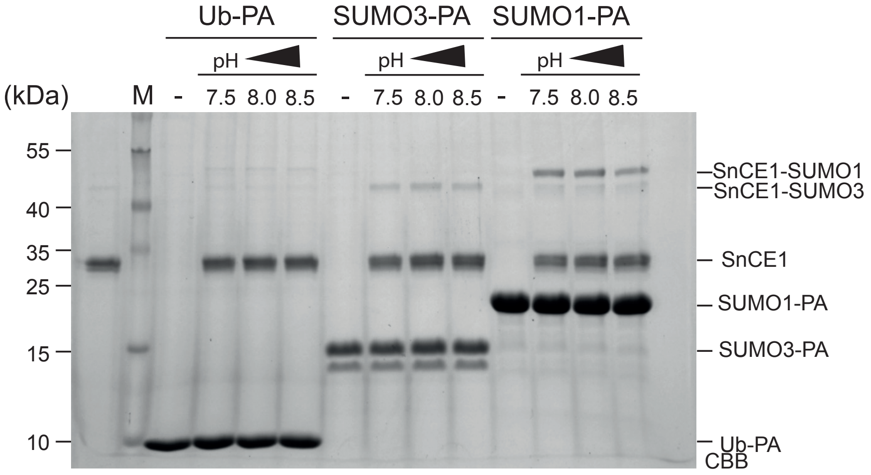

Supplementary Fig. 4: Changes in the pH-value from pH 7.5 to 8.5 have no effect on the reactivity of SnCE1 with the activity-based Ub-PA, SUMO1-PA and SUMO3-PA probes. The SDS-PAGE was stained with Coomassie brilliant blue (CBB).

a

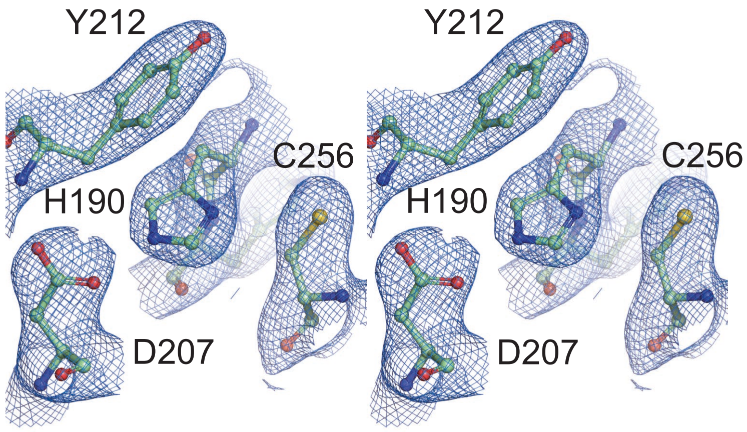

b

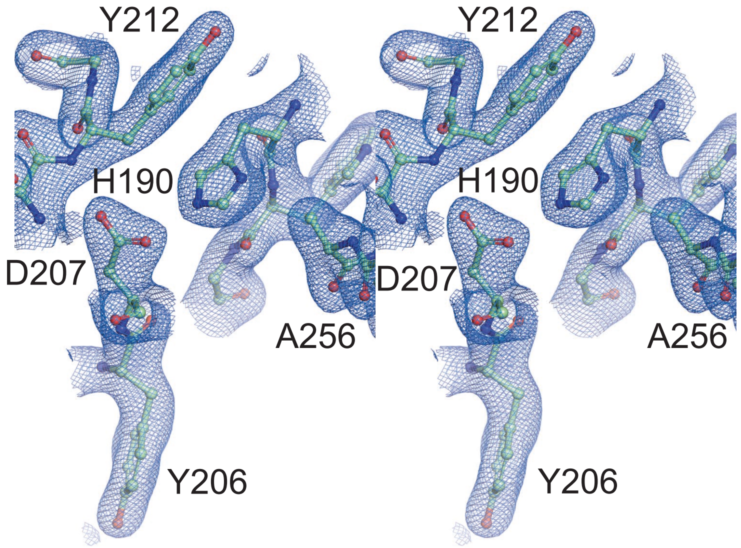

Supplementary Figure 5: Representative electron density for the apo structures of SnCE1 wildtype and SnCE1 C256A solved in this study.

Shown is a closeup of the active site including the catalytic triad residues, i.e. the nucleophile Cys256 or the replacement of Cys256 with Ala, the His190 base and the acid Asp207. SnCE1 backbone trace is coloured in green. The figure was prepared with PyMOL version 2.3.4[4]

a. Stereo figure of the active site in wall-eyed-viewing mode. Shown is the $2F_o$-F_c electron density map of a closeup of the active site for SnCE1 wildtype contoured at 1σ (PDB: 9QTE, [https://doi.org/10.2210/pdb9QTE/pdb]).

b. Stereo figure of the active site in wall-eyed-viewing mode. Shown is the $2F_o$-F_c electron density map of a closeup of the active site for catalytic mutant SnCE1 C256A contoured at 1σ (PDB: 9QTF, [https://doi.org/10.2210/pdb9QTF/pdb]).

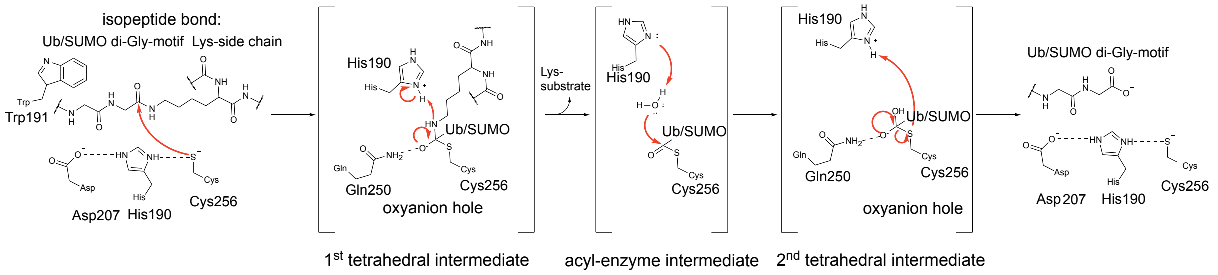

Supplementary Fig. 6: Proposed catalytic mechanism underlying the deSUMOylase activity of *S. negevensis* SnCE1. SnCE1 uses a catalytic triad consisting of His190 acting as general base/general acid, Asp207 acting as acid to position and polarizing His190 and Cys256 acting as nucleophile. Nucleophilic attack of the Cys256 on the isopeptide bond results in formation of a first tetrahedral intermediate, the isopeptide bond is cleaved initially releasing the C-terminal fragment, i.e. the lysine-containing SUMO/Ub/substrate protein, a covalent acyl-enzyme intermediate is formed. Attack of a water molecule as nucleophile resolves the covalent intermediate via formation of a second tetrahedral intermediate, here, His190 acting as acid to promote Cys256 leaving group departure. The tetrahedral intermediates with negative charge at the oxygen are stabilized by the oxyanion hole formed by amide side-chain of Gln250. The gatekeeper Trp191 restricts access to the active site to SUMO/Ub with a C-terminal diGly motif. The figure was created with ChemDraw Prime 23.0.1[5].

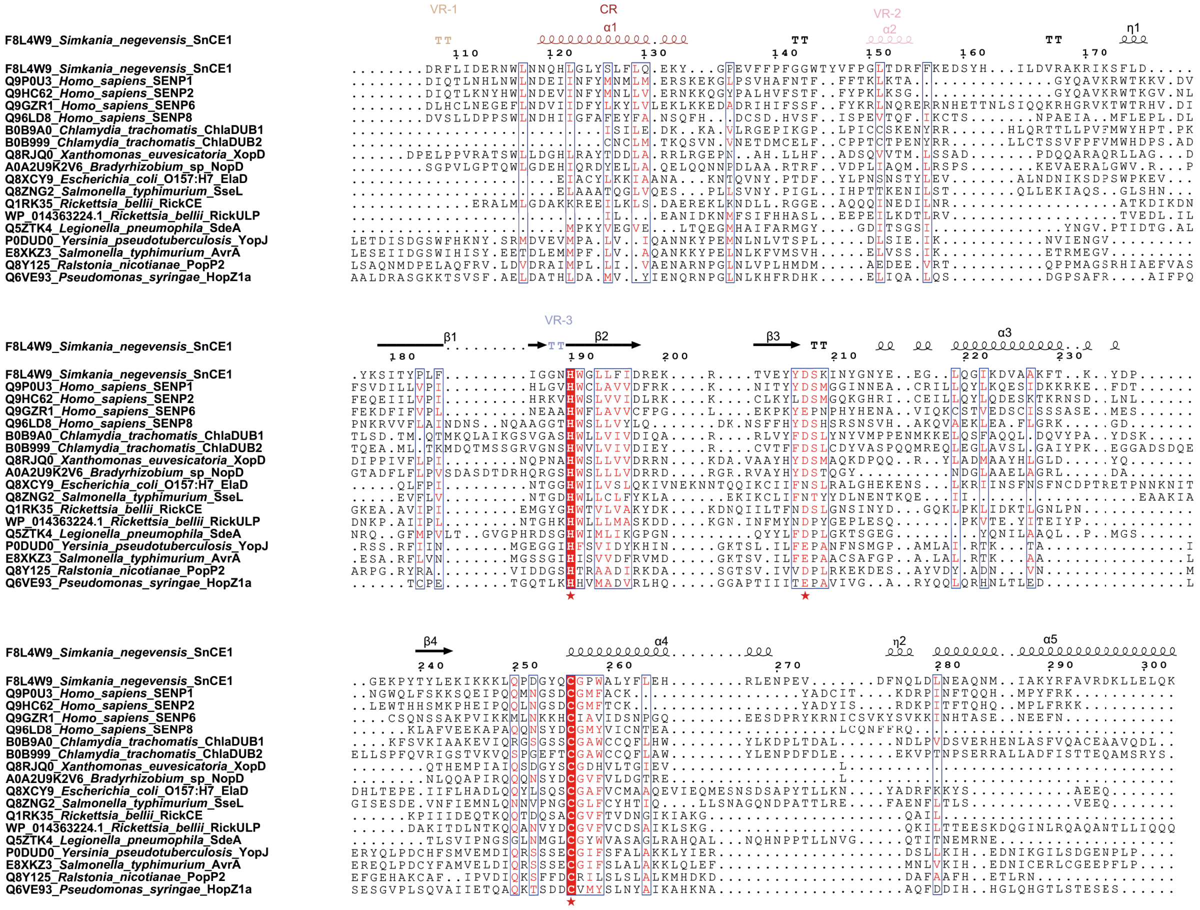

Supplementary Figure 7: Sequence alignment for the catalytic CE-clan protease domain for selected bacterial CE-clan protease-related DUBs/ULPs.

The alignment was conducted to uncover conserved and diverse regions in bacterial DUBs/ULPs. The following sequences for the CE-clan protease (α+β)-fold were used in the alignment: F8L4W9: *Simkania negevensis* SnCE1; Q9P0U3: *Homo sapiens* SENP1; Q9HC62: *Homo sapiens* SENP2; Q9GZR1: *Homo sapiens* SENP6; Q96LD8: *Homo sapiens* SENP8; B0B9A0: *Chlamydia trachomatis* ChlaDUB1; B0B999: *Chlamydia trachomatis* ChlaDUB2; Q8RJQ0: *Xanthomonas euvesicatoria* XopD; A0A2U9K2V6: *Bradyrhizobium* sp. NopD; Q8XCY9: *Escherichia coli* O157:H7 ElaD; Q8ZNG2: *Salmonella typhimurium* SseL; Q1RK35: *Rickettsia bellii* RickCE; WP_014363224.1: *Rickettsia bellii* RickULP; Q5ZTK4: *Legionella pneumophila* SdeA; P0DUD0: *Yersinia pseudotuberculosis* YopJ; E8XKZ3: *Salmonella typhimurium* AvrA; Q8Y125: *Ralstonia nicotianae* PopP2; Q6VE93: *Pseudomonas syringae* HopZ1a. The secondary structure elements are given on top for *S. negevensis* SnCE1. Red asterisks show the conserved residues of the catalytic triad, i.e. Asp207, His190 and Cys256 in SnCE1. The numbering is shown for SnCE1 full length. The variable regions 1-3 (VR-1-3) and the constant region (CR) are also indicated. The sequence alignments were conducted with ClustalW and ESPript 3.0 was used to render sequence similarities and information on secondary structure elements derived from the experimental structure of SnCE1 wildtype determined here (PDB: 9QTE) [https://doi.org/10.2210/pdb9QTE/pdb])[6].

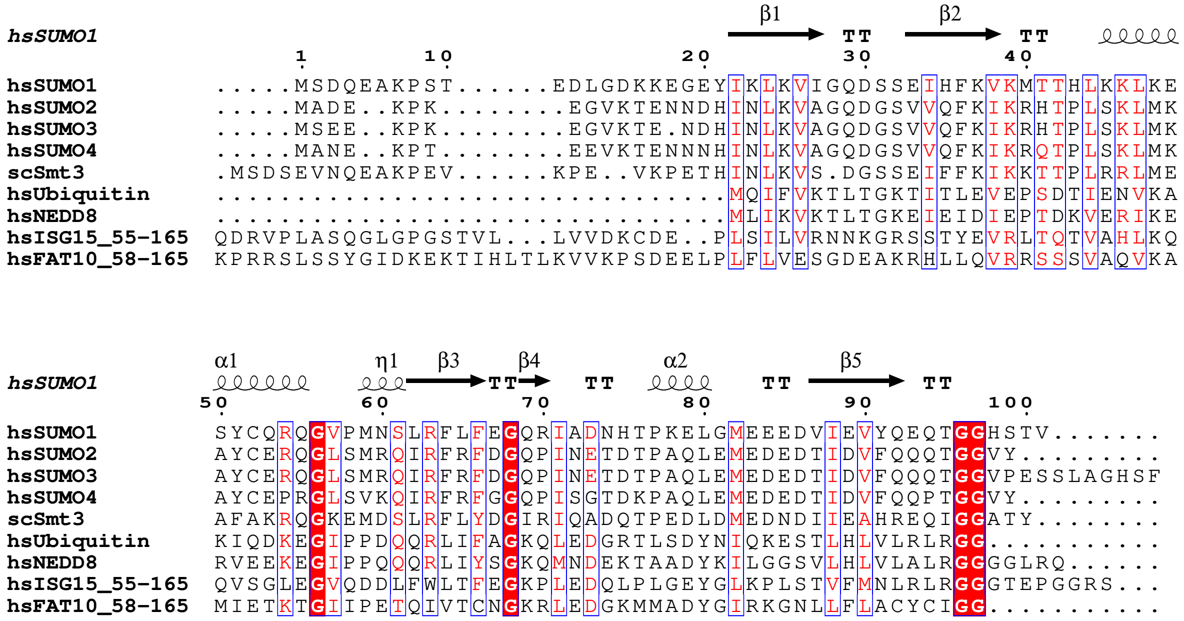

Supplementary Figure 8: Sequence alignment of human ubiquitin, SUMO1, SUMO2, SUMO3, SUMO4, NEDD8, ISG15 and FAT10. The alignment was conducted to uncover conserved and diverse regions in human ubiquitin and Ubl-proteins. The following sequences were used in the alignment: P63165: *Homo sapiens* SUMO1, hsSUMO1; P61956: *Homo sapiens* SUMO2, hsSUMO2; P55854: *Homo sapiens* SUMO3, hsSUMO3; P63165: *Homo sapiens* SUMO4, hsSUMO4; Q12306: *Saccharomyces cerevisiae* SMT3, scSMT3; P0CG47: *Homo sapiens* Ubiquitin, hsUb; Q15843: *Homo sapiens* NEDD8; hsNEDD8; P05161: *Homo sapiens* ISG15, hsISG15; O15205: *Homo sapiens* FAT10, hsFAT10. The secondary structure elements are given on top for SUMO1 from the SnCE1-SUMO1-PA structure determined here. The sequence alignments were conducted with ClustalW and ESPript 3.0 was used to render sequence similarities and information on secondary structure elements derived from the experimental structure of SnCE1 wildtype determined here (PDB: 9QTE) [https://doi.org/10.2210/pdb9QTE/pdb])[6].

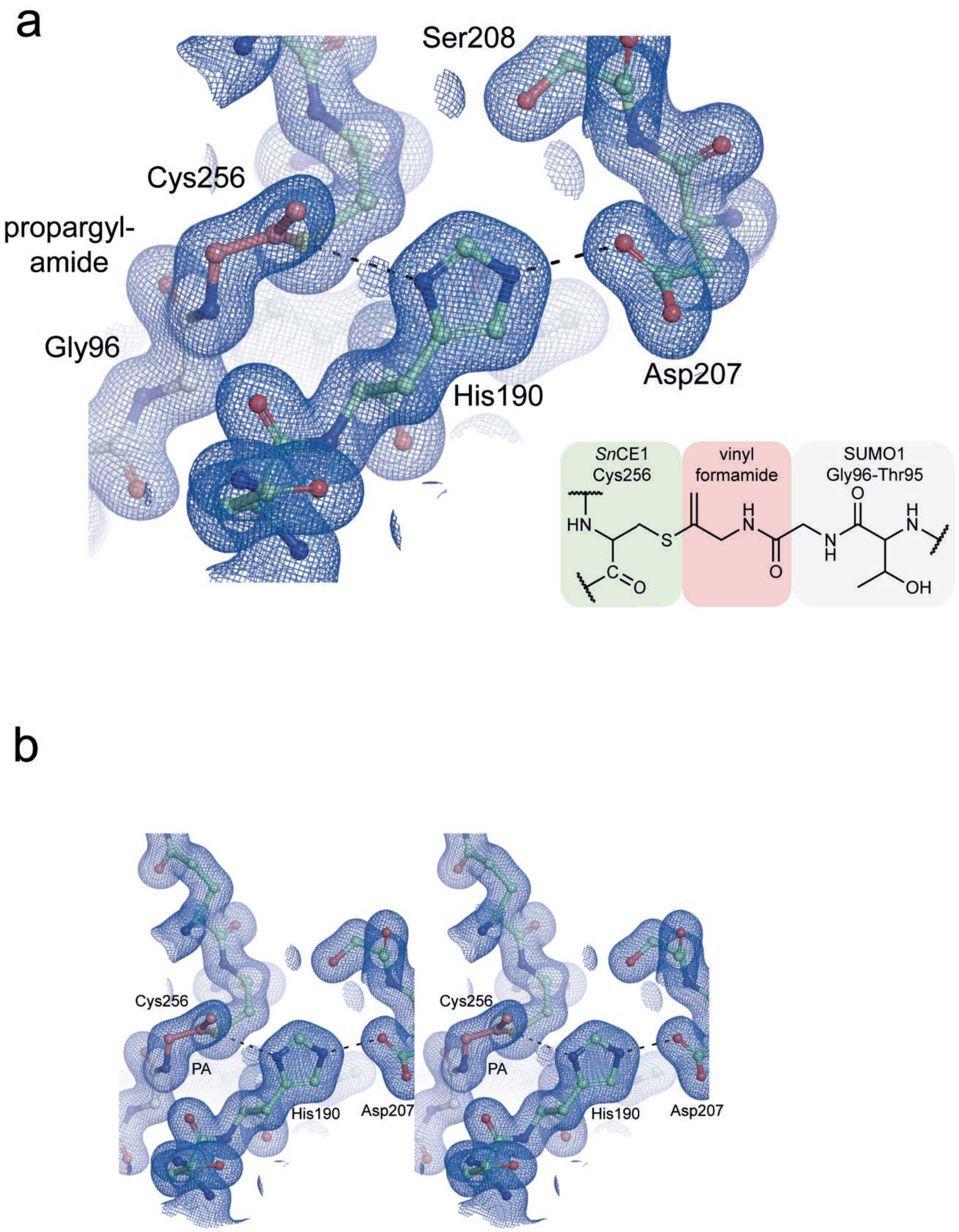

Supplementary Figure 9: Representative electron density for the structure SnCE1-SUMO1-PA solved in this study.

Shown is a closeup of the active site including the catalytic triad residues, i.e. the nucleophile Cys256 modified with propargylamide (PA) forming an amide bond to the C-terminal G96 of the SUMO1, the base His190 and the acid Asp207. The SUMO1 is coloured in grey, SnCE1 is coloured in green and the propargylamide moiety in red (PDB: 9QTG, [https://doi.org/10.2210/pdb9QTG/pdb]). The figures were prepared with PyMOL version 2.3.4[4]

a. Electron density $2F_o$-F_c map is contoured at 1σ and shown in blue. The scheme shows the active site architecture with the Cys256 linked by a vinyl formamide derived by reaction of the propargylamide bound to the C-terminal Gly96 of SUMO1 with the Cys256 thiol group forming a vinyl thioether.

b. Stereo figure of the active site in wall-eyed-viewing mode. Shown is the $2F_o$-F_c electron density map contoured at 1σ.

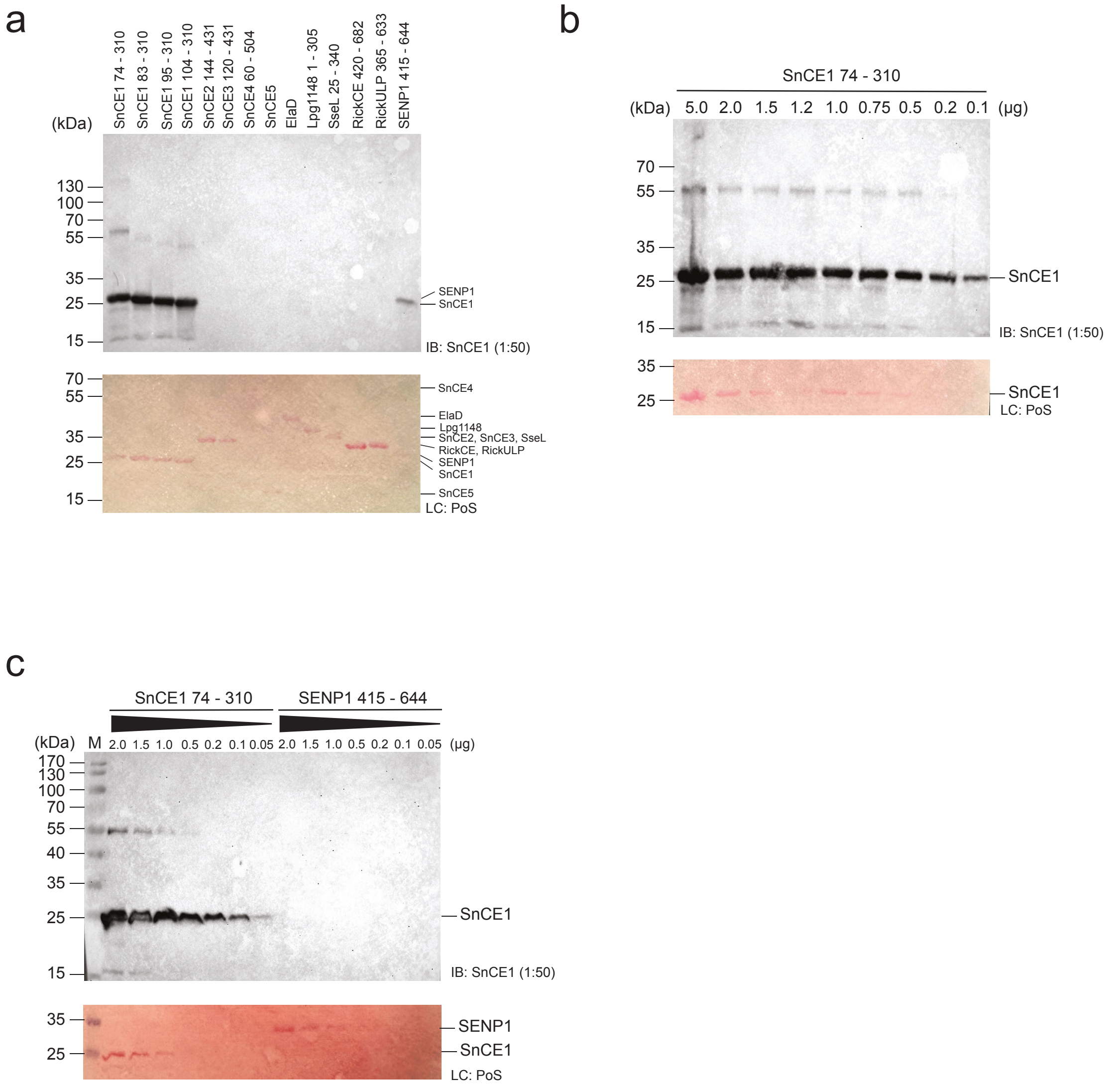

Supplementary Figure 10: Validation of antibody raised against recombinant SnCE1$_{104\text{-}310}$ protein regarding selectivity and sensitivity.

a. Selectivity of the SnCE1 antibody: Validation of the selectivity of the antibody raised against recombinantly expressed and purified *Simkania negevensis* SnCE1$_{74\text{-}310}$, SnCE1$_{83\text{-}310}$, SnCE1$_{95-310}$, SnCE1$_{104\text{-}310}$, *S. negevensis* SnCE2$_{144\text{-}431}$, *S. negevensis* SnCE3$_{120\text{-}431}$, *S. negevensis* SnCE$_{460\text{-}504}$, *S. negevensis* SnCE5, *Escherichia coli* ElaD, *Legionella pneumophila* Lpg1148$_{1\text{-}305}$, *Salmonella enterica* Thyphimurium. SseL$_{25\text{-}340}$, *Rickettsia bellii* RickCE$_{420\text{-}682}$, *Rickettsia* sp. RickULP$_{356\text{-}633}$ and *Homo sapiens* SENP1$_{415\text{-}644}$. The validation shows selectivity towards the *S. negevensis* SnCE1 constructs while the other *S. negevensis* proteins were not recognized. The N-terminal region of SnCE1 N-terminally from Gly104 is not recognized by the antibody. The antibody shows cross-reactivity with human SENP1 requiring pre-clearing for studies in human cells. Immunoblotting was done with the anti-SnCE1 antibody (IB: SnCE1), loading control was done by Ponceau S red staining of the membrane (LC: PoS). Dilution of the SnCE1 antibody was 1:50.

b. Sensitivity of the SnCE1 antibody. The antibody detects less than 100 ng of SnCE1 protein. Immunoblotting was done with the anti-SnCE1 antibody (IB: SnCE1), loading control was done by Ponceau S red staining of the membrane (LC: PoS). Dilution of the SnCE1 antibody was 1:50.

c. Pre-clearing of the SnCE1 antibody. To prevent cross-reactivity of the SnCE1 antibody with human SENP1 in cellular studies, a pre-clearing step was done by incubation of the antibody with recombinantly expressed human SENP1 protein. This enables to increase the specificity of the SnCE1 antibody. The dilution row shows sensitivity of the SnCE1 antibody of detecting less than 50 ng SnCE1 protein. Immunoblotting was done with the anti-SnCE1 antibody (IB: SnCE1), loading control was done by Ponceau S red staining of the membrane (LC: PoS). Dilution of the SnCE1 antibody was 1:50.

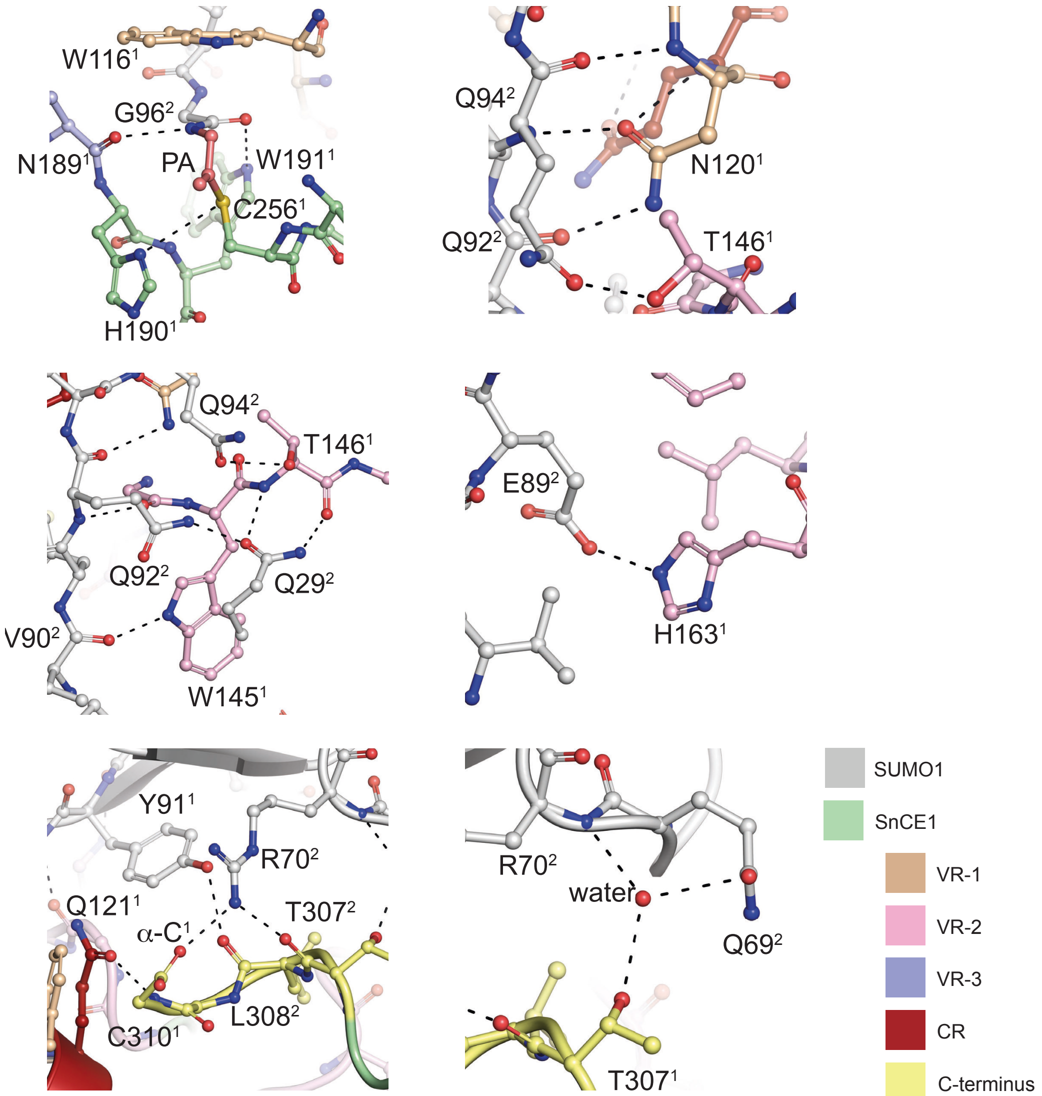

Supplementary Fig. 11: Closeup views of variable regions 1-3 (VR-1-3) of SnCE1 contributing to SUMO1 binding at S1.

Shown are closeups of the interactions of SUMO1 with residues of VR-1 (orange), VR-2 (magenta) and VR-3 (blue). Apart from these VRs, SnCE1 C-terminal residues (yellow) including the α-carboxylate contribute to SUMO1 binding. Apart from hydrophobic interactions contributing to the binding affinity most interactions of Ub to ChlaDUB1 are main-chain interactions particularly involving C-terminal residues of Ub. One of the rare electrostatic interactions is formed between positively-charged R72 of the C-terminal 72-RLRGG-76-motif in Ub inserted into a negatively-charged cavity formed by Asp167, Asp169 and Glu395 of ChlaDuB1[7]. SnCE1 specificity towards SUMO1 is created by formation of several side-chain interactions of SUMO1 with SnCE1 such as side-chain of Q94$^{\#2}$ with hydroxyl side-chain of Thr146^{1} in VR-2, Gln29$^{\#2}$ with main-chain of Thr146^{1}, side-chain of Tyr91$^{\#2}$ with main-chain of Leu308$^{\#1}$ in the SnCE1 C-terminal region and side-chain of Glu89$^{\#2}$ with the imidazole side-chain of H163$^{\#1}$ (superscript #1: SnCE1; superscript #2: SUMO1). The indole side-chain of Trp145$^{\#1}$ in VR-2 forms a hydrogen bond to main-chain carbonyl of Val90$^{\#2}$. Gly143$^{\#1}$ in VR-2 is in hydrogen bond distance to the main-chain of Gln92$^{\#2}$. Moreover, SUMO1 Arg70$^{\#2}$ forms a side-chain interaction with the α-carboxylate of SnCE1 and with the main-chain of Thr307$^{\#1}$ at the SnCE1 C-terminus. The side-chain hydroxyl of Thr307$^{\#1}$ furthermore interacts with main-chain amide of Arg70$^{\#2}$ and side-chain of Gln69$^{\#2}$ of SUMO1 which is mediated by a precisely coordinated bridging water molecule. Additionally, the main-chain carbonyl of Asn189$^{\#1}$ in VR-3 directly binds the amide of the propargylamide connecting the C-terminus of SUMO1 and the Cys256$^{\#1}$ nucleophile. Trp116$^{\#1}$ of VR-1 forms a stacking interaction with the SUMO1 C-terminal residues and gatekeeper Trp191$^{\#1}$ makes a hydrogen bond with its indole side-chain and the main-chain carbonyl of the C-terminal Gly96$^{\#2}$ in SUMO1 sterically restricting access for nucleophilic attack of Cys256$^{\#1}$. The side-chain of SnCE1 Asn120$^{\#1}$ in VR-1 creates hydrogen bonds to the main-chain amides of Gln92$^{\#2}$ and Gln94$^{\#2.}$ in the SUMO1 C-terminus.

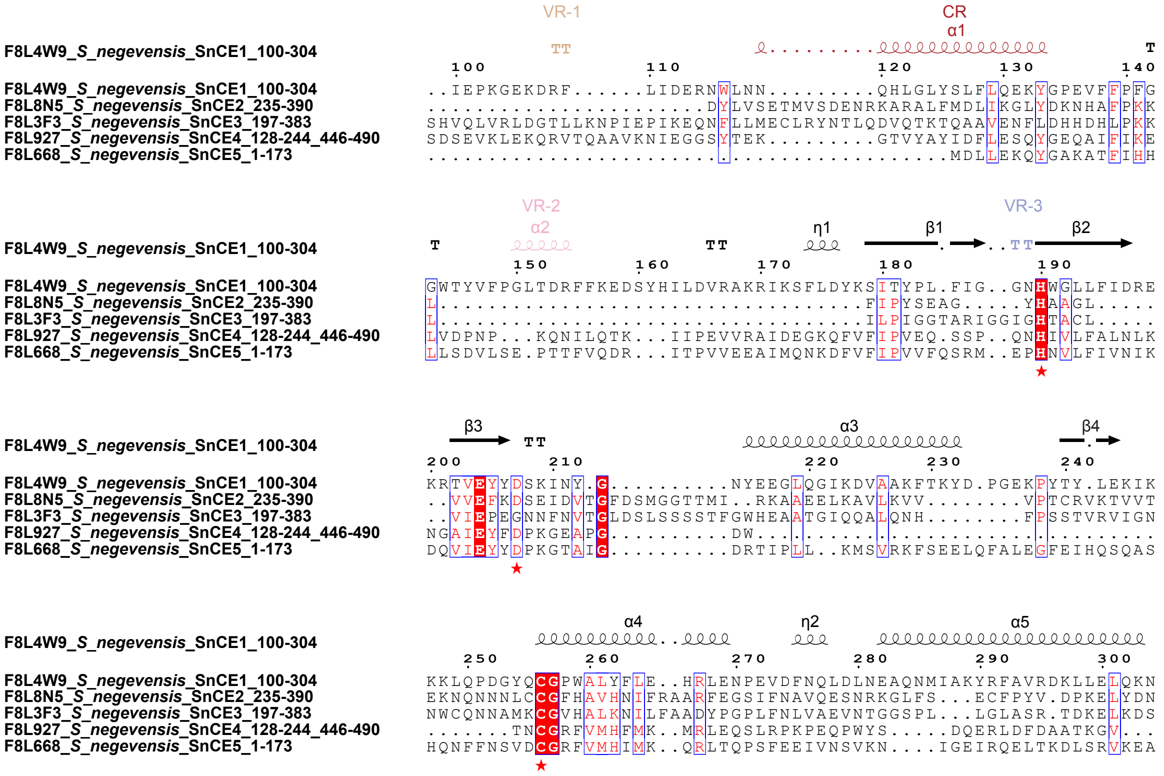

Supplementary Figure 12: Sequence alignment for the catalytic domains of *S. negevensis* CE-clan protease-related proteins SnCE1-5. The alignment was conducted to uncover conserved regions in *S. negevensis* CE-clan protease-related proteins. The following sequences were used in the alignment: F8L4W9: *S. negevensis* SnCE1, F8L8N5: *S. negevensis* SnCE2; F8L3F3: *S. negevensis* SnCE3; F8L927: *S. negevensis* SnCE4; F8L668: *S. negevensis* SnCE5. Red asterisks show the conserved residues of the catalytic triad, i.e. His190, Asp207, and Cys256 in SnCE1. In SnCE3 the catalytic triad Asp is replaced by Gly. The secondary structure elements are given on top for the SnCE1 structure determined here. Indicated are the variable regions 1-3 (VR1-3) and the constant region (CR). The sequence alignments were conducted with ClustalW and ESPript 3.0 was used to render sequence similarities and information on secondary structure elements derived from the experimental structure of SnCE1 wildtype determined here (PDB: 9QTE) [https://doi.org/10.2210/pdb9QTE/pdb])[6].

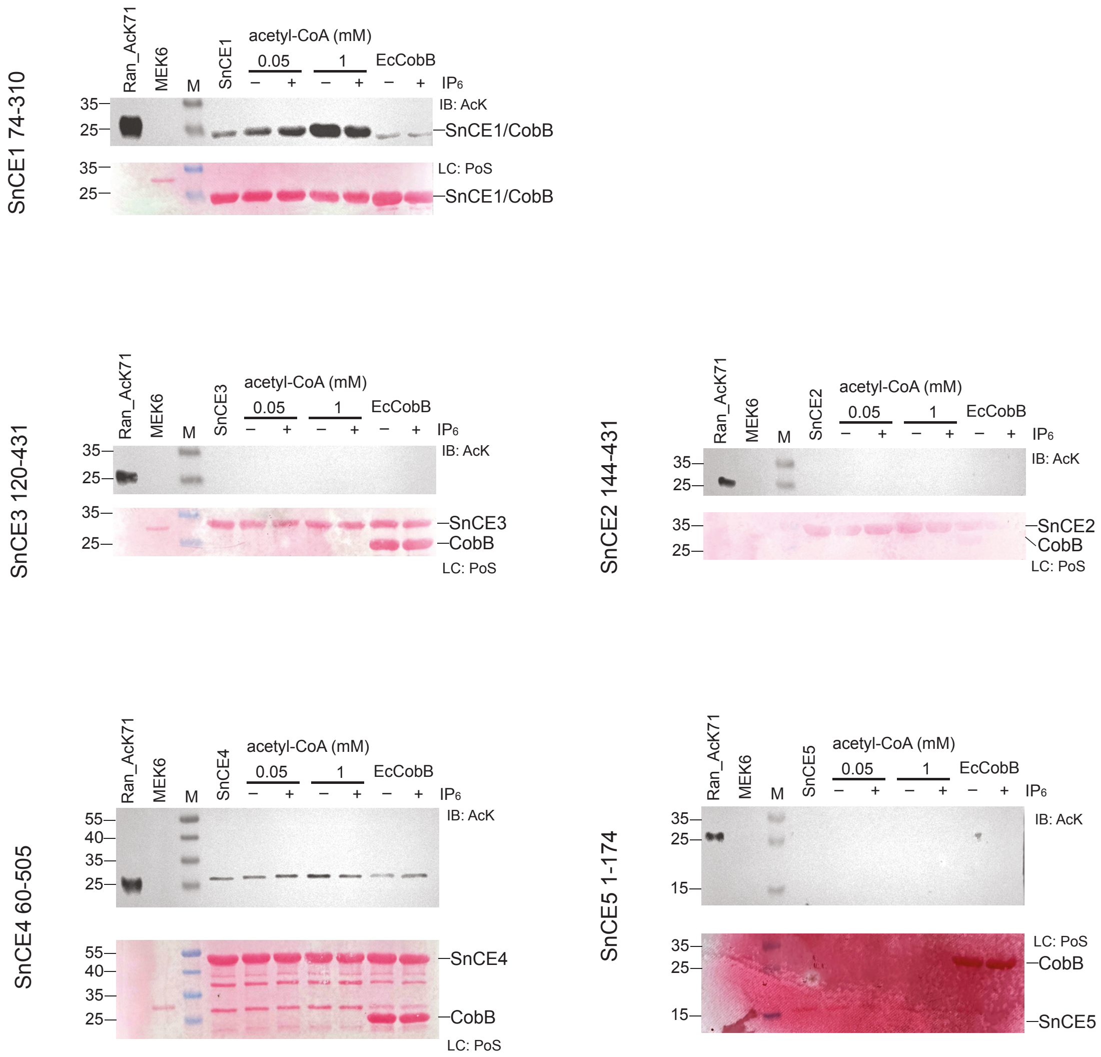

Supplementary Fig. 13: Analysis of the acetylation state of *S. negevensis* SnCE1-SnCE5. From the five CE-clan protease-related DUBs/ULPs, i.e. SnCE1-SnCE5, in *S. negevensis* only SnCE1 is lysine acetylated. Addition of acetyl-CoA increases the acetylation signal for SnCE1. *E. coli* CobB, *Ec*CobB, is not capable to deacetylate SnCE1. Presence of the activator inositol hexakisphosphate (IP6) does not enhance acetyltransferase activity as described for YopJ-family acetyltransferases, i.e. YopJ, AvrA, PopP2[8-10]. Immunoblotting was done with the anti-acetyl-lysine antibody (IB: AcK), loading control was done by Ponceau S red staining of the membrane (LC: PoS). Source data are provided as Source Data file.

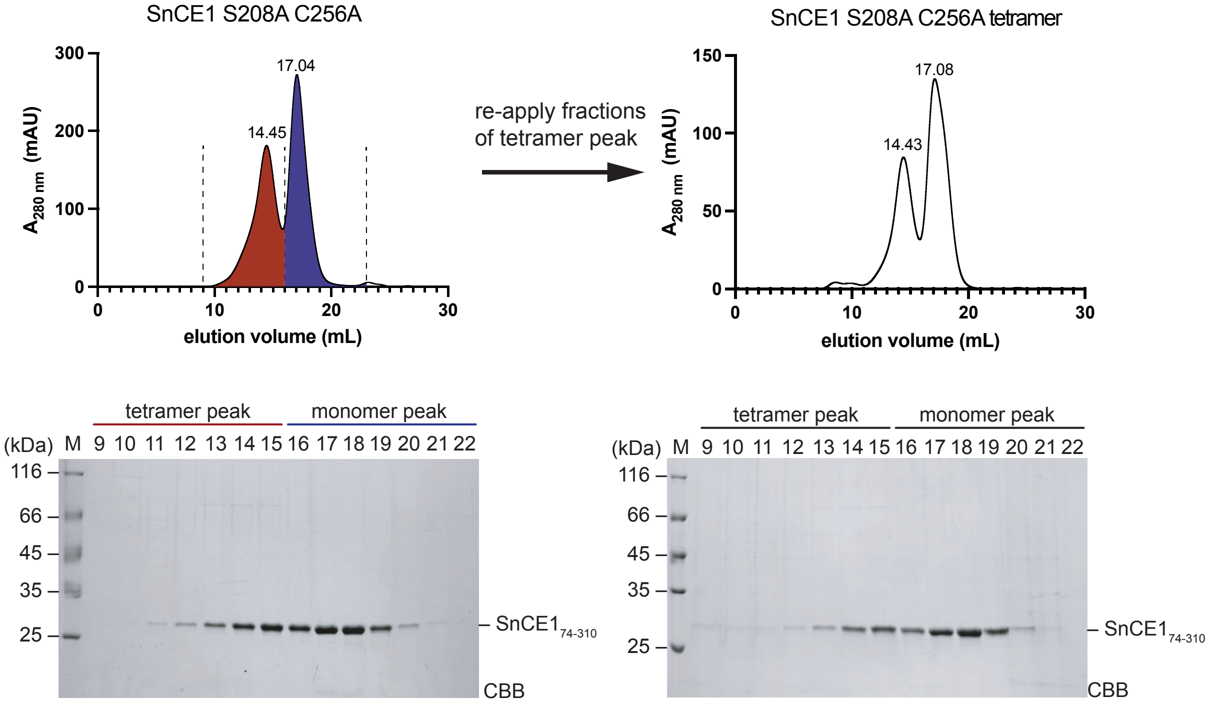

Supplementary Figure 14: The tetramer formed by non-acetylated SnCE1 S208 C256A exists in an equilibrium with the monomer.

To investigate if non-acetylated SnCE1 forms a stable tetramer of if this tetramer exists in an equilibrium with a monomer, we performed analysical size-exclusion chromatography (SEC) experiments. The tetramer fractions of an analytical SEC run were pooled, concentrated and analysed again by analytical SEC. This shows that the SnCE1 S208A C256A tetramer splits again into a tetramer and a monomer showing that the tetramer is in a dynamic equilibrium with a monomer. This suggests the tetramer being of moderate affinity in solution, however, it might be more stable upon dimensionality reduction if bound to a membrane in cells. 0.1 to 0.2 mg of protein was analysed on a Superdex 200 Increase 10/300 GL column. Below the SEC elution profiles showing the absorption at 280 nm ($A_{280\ nm}$) in mAU (milli absorbance units) the SDS-PAGE gels were shown analysing fractions of the observed absorption peaks. SDS-PAGE gels of the fractions of the SEC runs and of the deSUMOylase assay were stained using Coomassie brilliant blue (CBB). The experiment was performed once (*n*=1). Source data are provided as Source Data file.

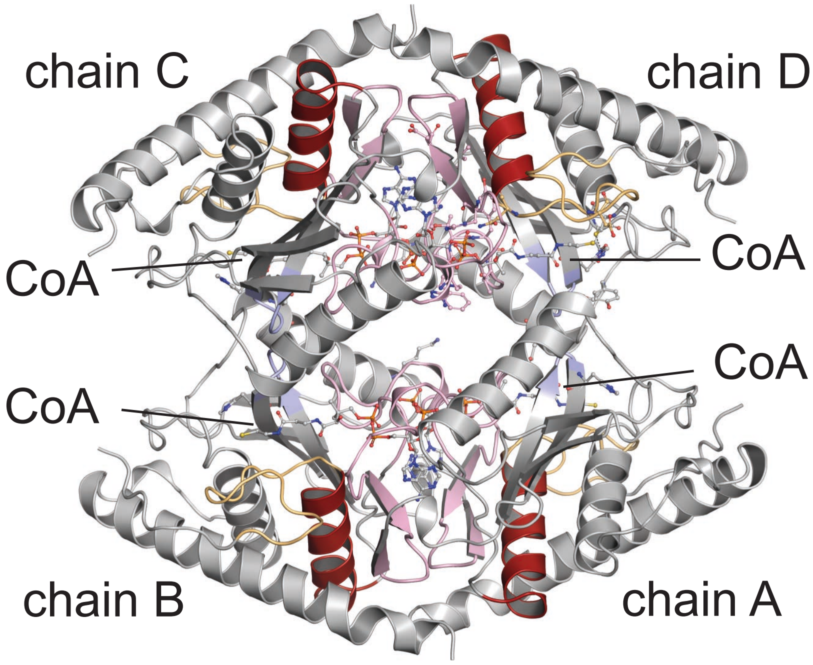

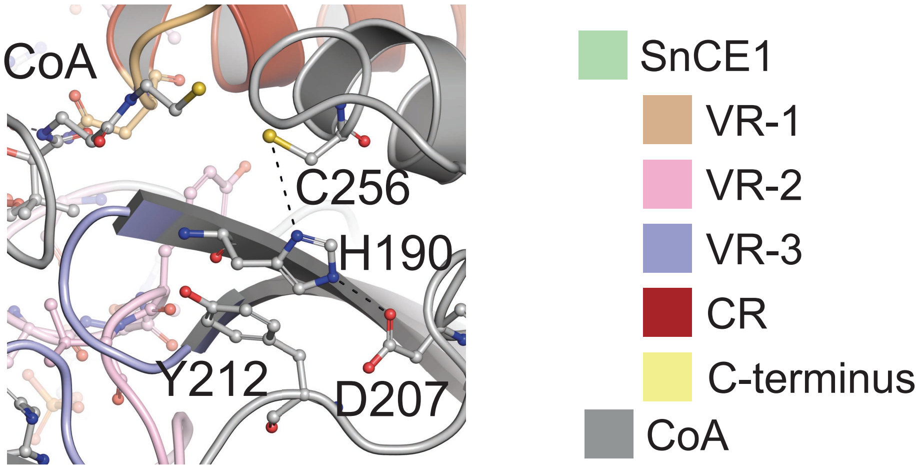

Supplementary Fig. 15: AlphaFold3 structure prediction of a SnCE1•CoA tetramer. The prediction has pTM+ipTM value of 0.38+0.48 suggesting this prediction is not representing the true tetramer[1-3]. In contrast to the experimental structure, the AlphaFold3 model, the CoA is bound by two neighboring chains of the tetramer. The CoA molecule is not directly interacting with Tyr212 and some residues sterically clash with the CoA molecule, i.e. the side chain of Asn119, and the main chain carbonyl oxygen of L164 of the neighboring chain and the exocyclic amino group of the CoA adenine base[1-3].

a

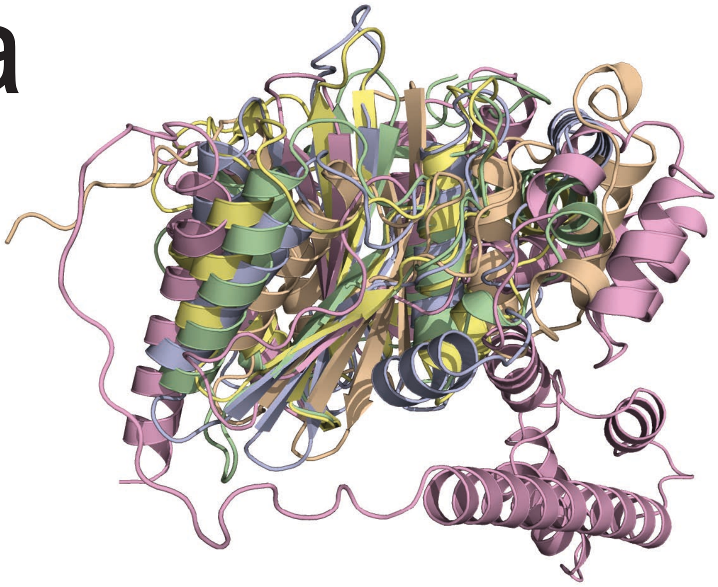

protein		AF3 confidence score (pTM)	ranking score	r.m.s.d. [Å]
SnCE2		0.61	0.65	5.0744
SnCE3		0.81	0.84	2.8467
SnCE4		0.32	0.44	2.6536
SnCE5		0.85	0.85	2.0960

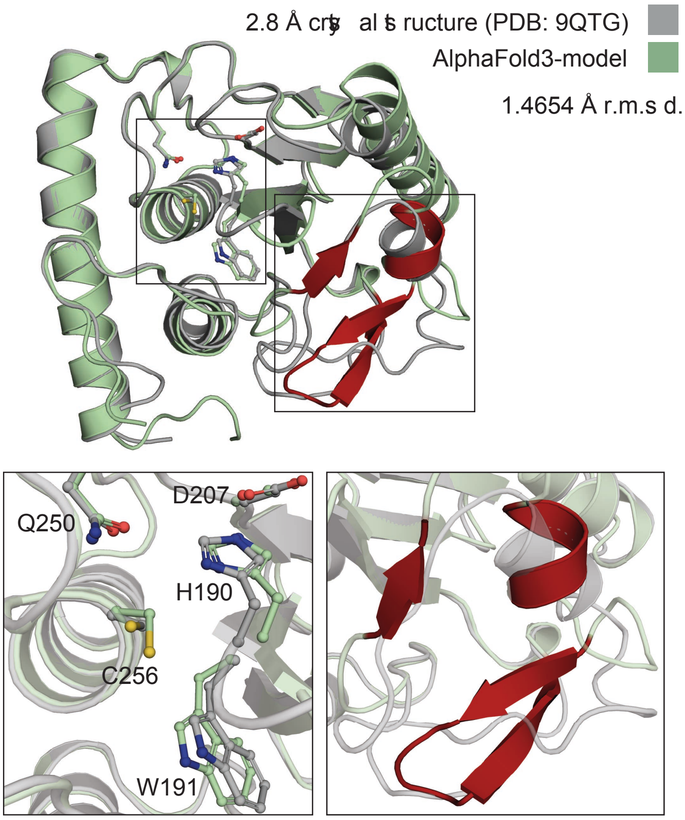

b

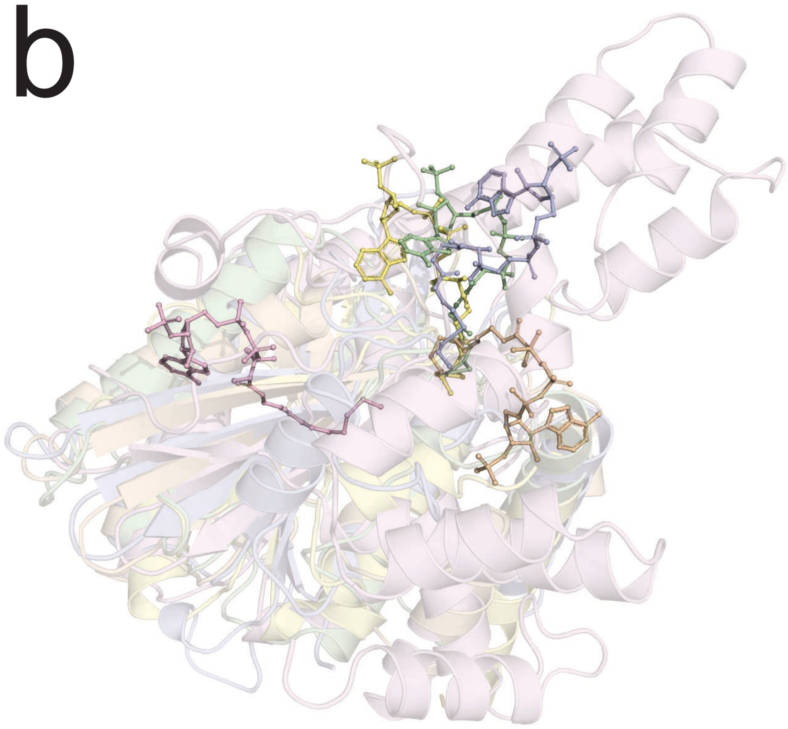

protein complex		AF3 confidence score (ipTM + pTM)	ranking score	r.m.s.d. [Å]
SnCE1*CoA		0.85 + 0.67	0.97	–
SnCE2*CoA		0.79 + 0.61	0.79	5.7491
SnCE3*CoA		0.80 + 0.55	0.79	2.5023
SnCE4*CoA		0.48 + 0.38	0.62	3.3502
SnCE5*CoA		0.77 + 0.78	0.78	2.2371

SnCE1	SnCE2	SnCE3	SnCE4	SnCE5
W116	T197	T410	Y152	–
N189	Y281	G271	N219	P61
H190	H282	H272	H220	H62
K209	M303	L293	K239	K81
Y212	T306	F301	A241*	A84*
G213	T307	T298	P242*	I85*
Q250	Q338	S292	–	Q121
G253	N341	M334	G445	S126
Y254	C343	K335	T448	V127
Q255	L342	V338	N449	D128

*amino acids correspond to their position in the sequence alignment of SnCE1-5 (Suppl. Fig. 12)

c

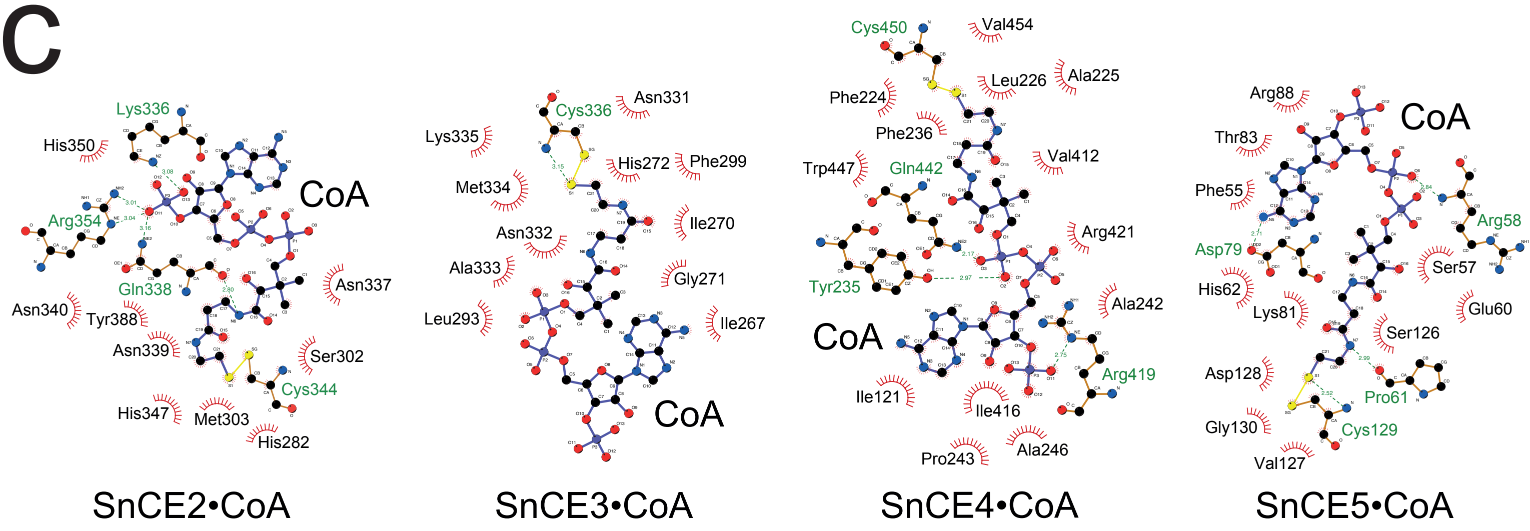

Supplementary Fig. 16: AlphaFold3 structure predictions of SnCE1, SnCE2, SnCE3, SnCE4 and SnCE5 in their apo forms and in complexes with CoA.

a.,b. The models obtained for the apo-proteins (a) and the CoA-bound forms (b) were superposed using secondary-structure matching (SSM) in COOT (v0.9.6) resulting in the r.m.s.d. values shown in the tables which indicate the structural similarity to the reference structure, i.e. the experimental SnCE1 wildtype structure or the AlphaFold3 model of SnCE1•CoA, respectively[11]. The quality indicators of the AlphaFold3 predictions, pTM-values (pTM: predicted template modelling score; assesses the accuracy of the global structure) for the apo-proteins, and pTM+ipTM-scores (ipTM: interface-predicted template modelling score; measures the accuracy of the predicted relative positions of the subunits of a complex) for the complexes with CoA, were listed[1-3,12]. To enable a comparison of the predicted CoA-binding sites in SnCE2-5 with SnCE1 the table in b shows the residues of SnCE1-5 involved in binding of CoA. For residues marked with red asterisks the structural superposition did reveal absence of a side chain. In these cases, the residues of the alignment were shown.

c. The experimental data show that besides SnCE1 none of the other CE-clan protease-related virulence factors of *S. negevensis* have an acetyltransferase (AcT) activity. The suggested interactions of CoA and the respective CE-clan protease were determined using the AlphaFold3 structural models and LigPlot[13,14]. Several residues involved in CoA-binding in SnCE1 are not conserved in SnCE2-5 as shown also in the table in b. This includes an aromatic side chain, i.e. Tyr212 in SnCE1 being essential for AcT activity, is only present in SnCE1[1-3]. The AlphaFold3 models were available as Supplementary Data.

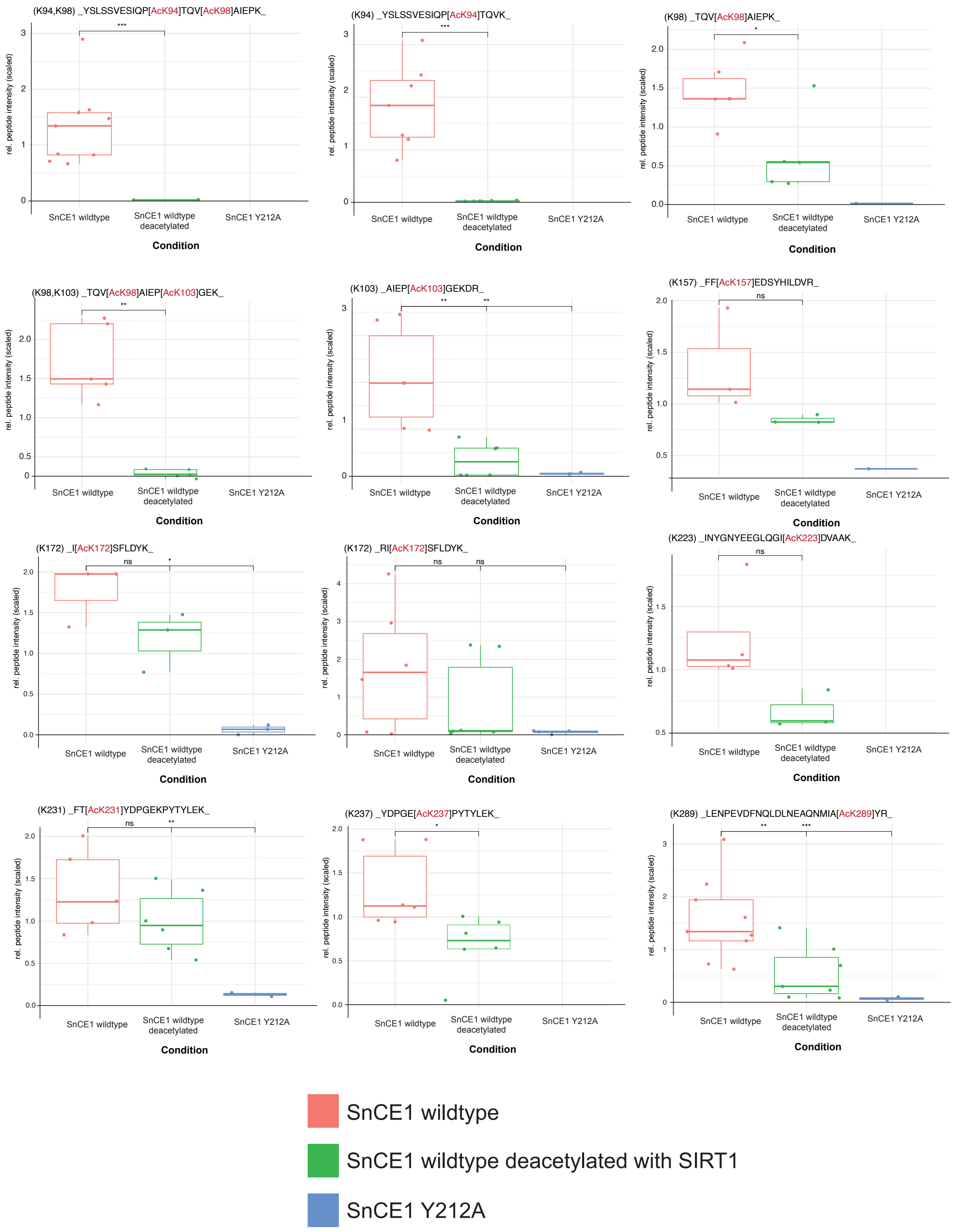

Supplementary Fig. 17: Lysine acetylation sites identified in SnCE1 by mass spectrometry. Recombinant SnCE1 wildtype protein was analysed by tryptic digest and LC-MS/MS to identify the acetylation sites. Besides, SnCE1 wildtype was deacetylated by human SIRT1 to discover which sites are accessible to enzymatic deacetylation. AcK94, AcK98, AcK103, AcK237 and AcK189 are deacetylated by SIRT1, AcK157, AcK172, AcK223 and AcK231 are not. Non-acetylated SnCE1 Y212A served as control. Shown are whisker plots showing a box indicating the upper and lower quartiles and the median values. The maximum and minimal values are shown. Statistical significance between the different samples was determined using unpaired, two-sided Student's t-tests with p-values < 0.05 considered significant.

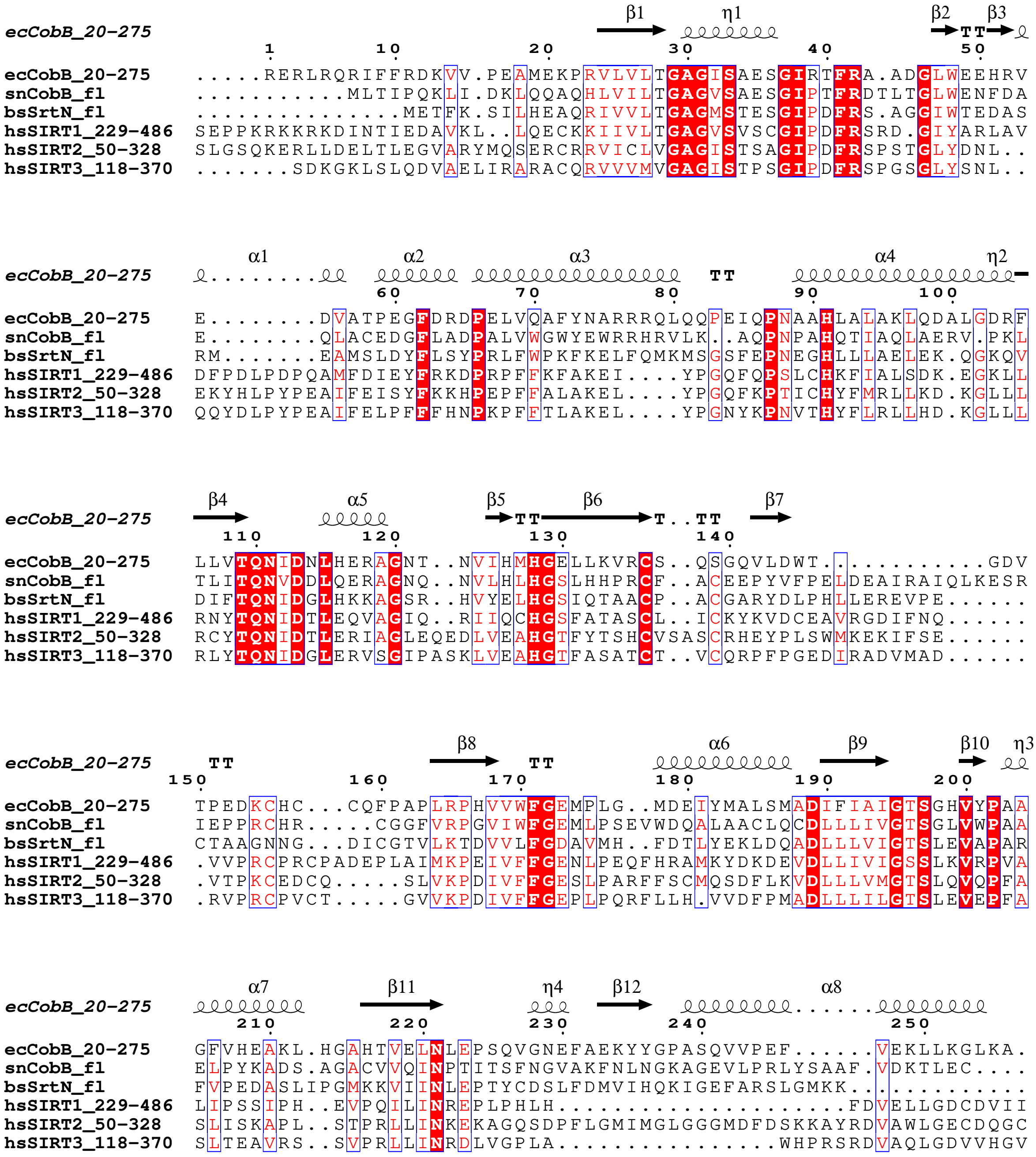

Supplementary Fig. 18: *Simkania negevensis* encodes for a single sirtuin deacetylase CobB (SnCobB).

Sequence alignment of bacterial and human sirtuins, i.e. human SIRT1, SIRT2, SIRT3, *B. subtilis* SrtN, *E. coli* CobB and *S. negevensis* CobB. The alignment covers the active site uncovering conserved residues. The following sequences were used in the alignment: P0CG47: *Homo sapiens* Ubiquitin, hsUb; Q96EB6: *Homo sapiens* SIRT1, hsSIRT1; Q8IXJ6: *Homo sapiens* SIRT2, hsSIRT2; Q9NTG7: *Homo sapiens* SIRT3, hsSIRT3; A0A6M4JHV4: *Bacillus subtilis* SrtN: bsSrtN; P75960: *Escherichia coli* CobB, ecCobB; A0A942ELM6: *Simkania* sp. CobB, snCobB. The secondary structure elements are given on top for the AlphaFold3 model of SnCobB•NAD^+ from the SnCE1(pTM+iPTM: 0.91+0.95). The sequence alignments were conducted with ClustalW and ESPript 3.0 was used to render sequence similarities and information on secondary structure elements derived from the AlphaFold3 model for SnCobB.

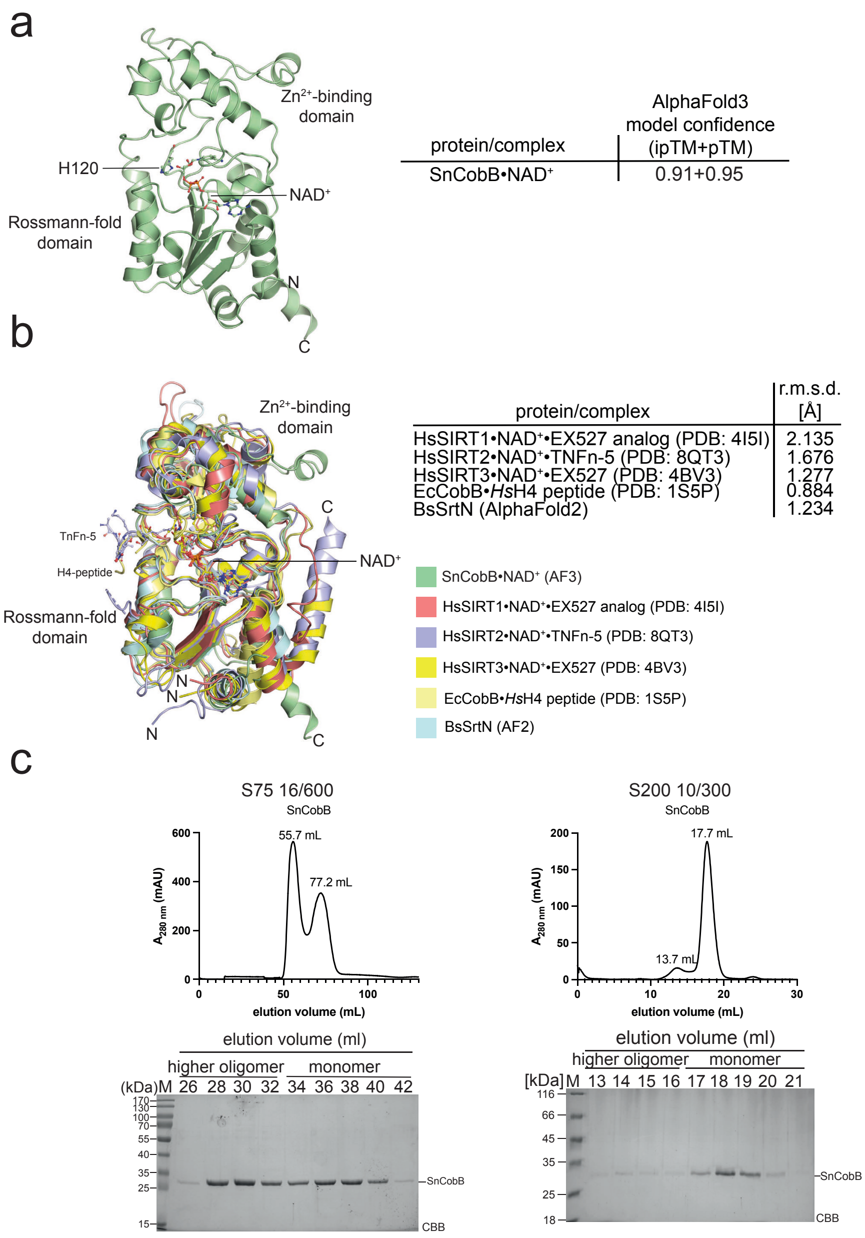

Supplementary Fig. 19: AlphaFold3 structure prediction for *Simkania negevensis* CobB (SnCobB).

a. AlphaFold3 model of SnCobB•NAD+ from the SnCE1(pTM+ipTM: 0.91+0.95). The model was created with the AlphaFold3 server (https://alphafoldserver.com/) and shows a typical domain organization with a Rossmann-fold domain and a Zn^{2+}-binding domain with the substrate and NAD+-binding site in the interface[1-3].

b. Superposition of the AlphaFold3 model of SnCobB•NAD+ with selected sirtuins as indicated. The highest structural similarity of SnCobB•NAD+ is observed towards the bacterial sirtuins CobB from *E. coli* (*Ec*CobB) (r.m.s.d.: 0.884 Å) and SrtN from *B. subtilis* (*Bs*SrtN) (r.m.s.d.: 1.234 Å). Shown are the following structures: *Hs*SIRT1•NAD+•EX527 (PDB: 4I5I [https://doi.org/10.2210/pdb4I5I/pdb]), *Hs*SIRT2•NAD+•TNFn-5 (PDB: 8QT3 [https://doi.org/10.2210/pdb8QT3/pdb]), *Hs*SIRT3•NAD+•EX527 (PDB: 4BV3 [https://doi.org/10.2210/pdb4BV3/pdb]), *Ec*CobB•HsH4 peptide (PDB: 1S5P [https://doi.org/10.2210/pdb1S5P/pdb]), AlphaFold2 of *Bs*SrtN.

c. SnCobB elutes as higher oligomer and monomer from SEC. The preparative SEC (S75 16/600) suggests CobB forms a higher oligomer and a monomer. This is confirmed on a calibrated analytical S200 10/300 column. The elution volumes and calculated molecular weights (calc. MW) were used in comparison to expected molecular weights (exp. MW) to calculate the apparent oligomeric state (Supplementary Fig. 2). A_{280} is the absorption at 280 nm. mAU: milli absorbance units. SDS-PAGEs were stained with Coomassie brilliant blue (CBB). Source data are provided as Source Data file.

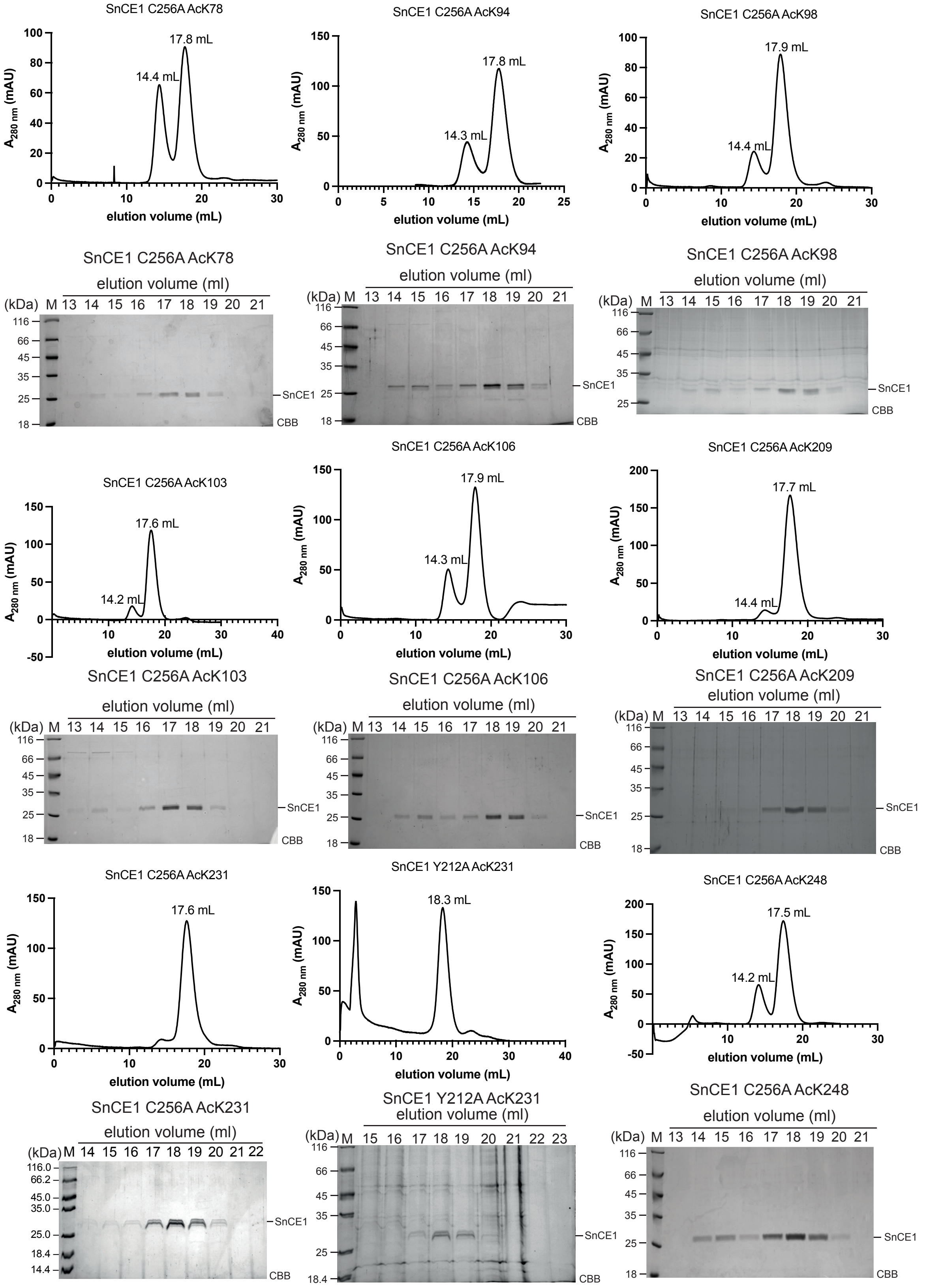

Supplementary Fig. 20: Analytical size-exclusion chromatography (SEC) of site-specifically lysine acetylated SnCE1 C256A proteins prepared by GCEC. Apart from SnCE1 C256A AcK231, all other proteins elute in a monomer-tetramer equilibrium. SnCE1 C256A AcK231 elutes as monomer. The elution volumes and calculated molecular weights (calc. MW) were used in comparison to expected molecular weights (exp. MW) to calculate the apparent oligomeric state of the proteins. The calculated molecular weights were obtained based on the elution volume using a calibration curve shown in Supplementary Fig. 2. A_{280} is the absorption at 280 nm. mAU: milli absorbance units. The SDS-PAGE gels were stained with Coomassie brilliant blue (CBB). Source data are provided as Source Data file.

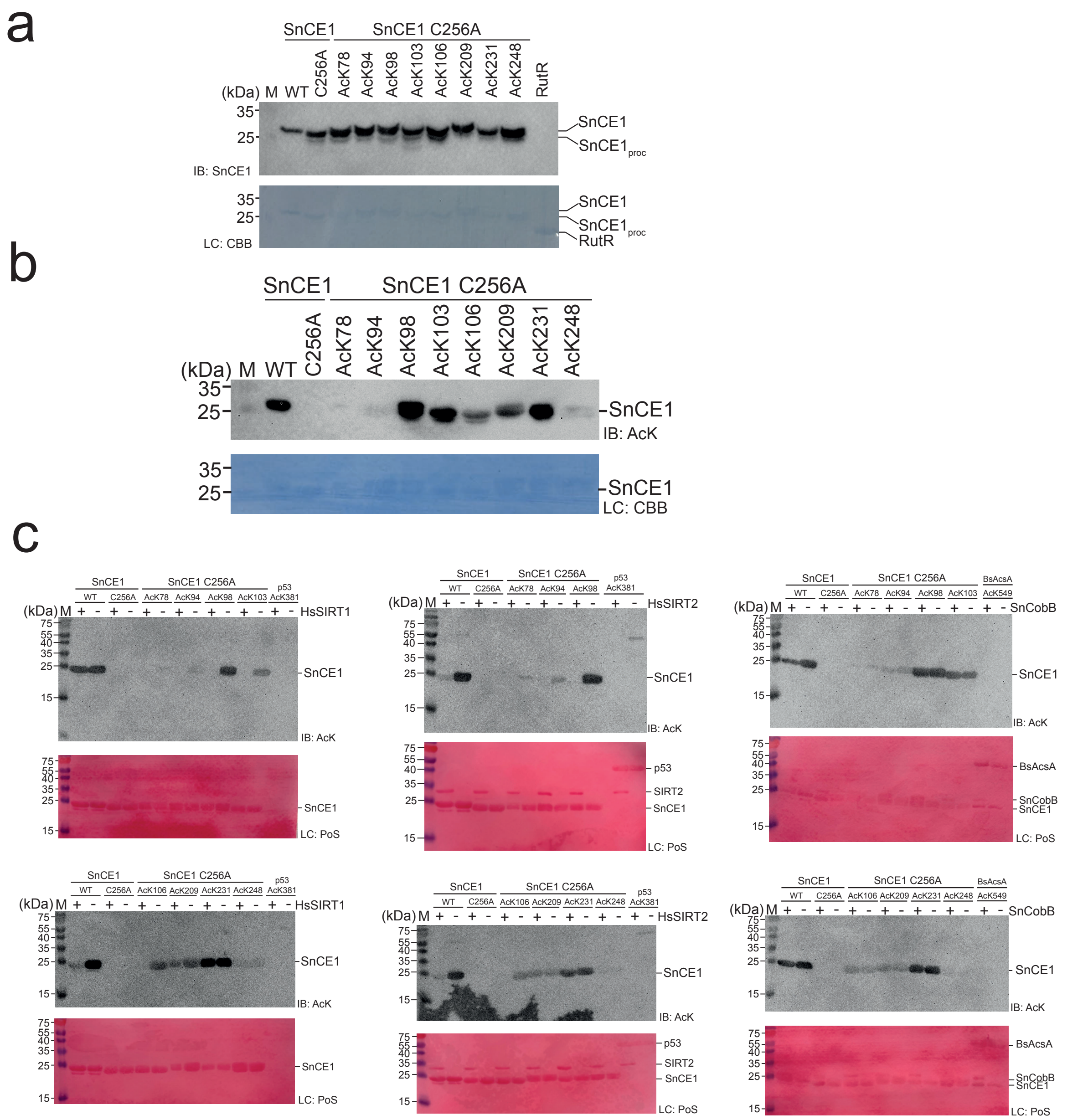

Supplementary Figure 21: Site-specific incorporation of acetyl lysine into SnCE1 using the genetic code expansion concept (GCEC).

a Site-specifically lysine acetylated SnCE1 C256A proteins prepared by GCEC were analysed by immunoblotting using a SnCE1 antibody (IB: SnCE1). Shown are SnCE1 C256A AcK78, SnCE1 C256A AcK94, SnCE1 C256A AcK98, SnCE1 C256A AcK103, SnCE1 C256A AcK106, SnCE1 C256A AcK209, SnCE1 C256A AcK231, SnCE1 C256A AcK248. Coomassie brilliant blue (CBB) staining was done as loading control (LC: CBB). Source data are provided as Source Data file.

b Immunoblotting of site-specifically lysine acetylated SnCE1 C256A proteins prepared by GCEC, expression in *E. coli* and purification. Shown are SnCE1 C256A AcK78, SnCE1 C256A AcK94, SnCE1 C256A AcK98, SnCE1 C256A AcK103, SnCE1 C256A AcK106, SnCE1 C256A AcK209, SnCE1 C256A AcK231, SnCE1 C256A AcK248. Staining of the immunoblot was done using an anti-acetyl lysine antibody (IB: AcK). The staining with the anti-AcK antibody shows some sites are only weakly detected by the antibody. Coomassie brilliant blue (CBB) staining was done as loading control (LC: CBB). Source data are provided as Source Data file.

c Deacetylation of SnCE1 by human SIRT1, SIRT2 and by *S. negevensis* SnCobB. SnCE1 and mutants thereof were deacetylated with SIRT1 and the acetylation state was assessed by immunoblotting with anti-acetyl-lysine antibody (IB. AcK). The acetylated proteins SnCE1 C256A AcK209, SnCE1 C256A AcK231 and SnCE1 C256A AcK248 were not deacetylated by any deacetylase tested supporting acetylation at K231 being irreversible. Loading control was done with Ponceau S red staining of the membrane (LC: PoS). Source data are provided as Source Data file.

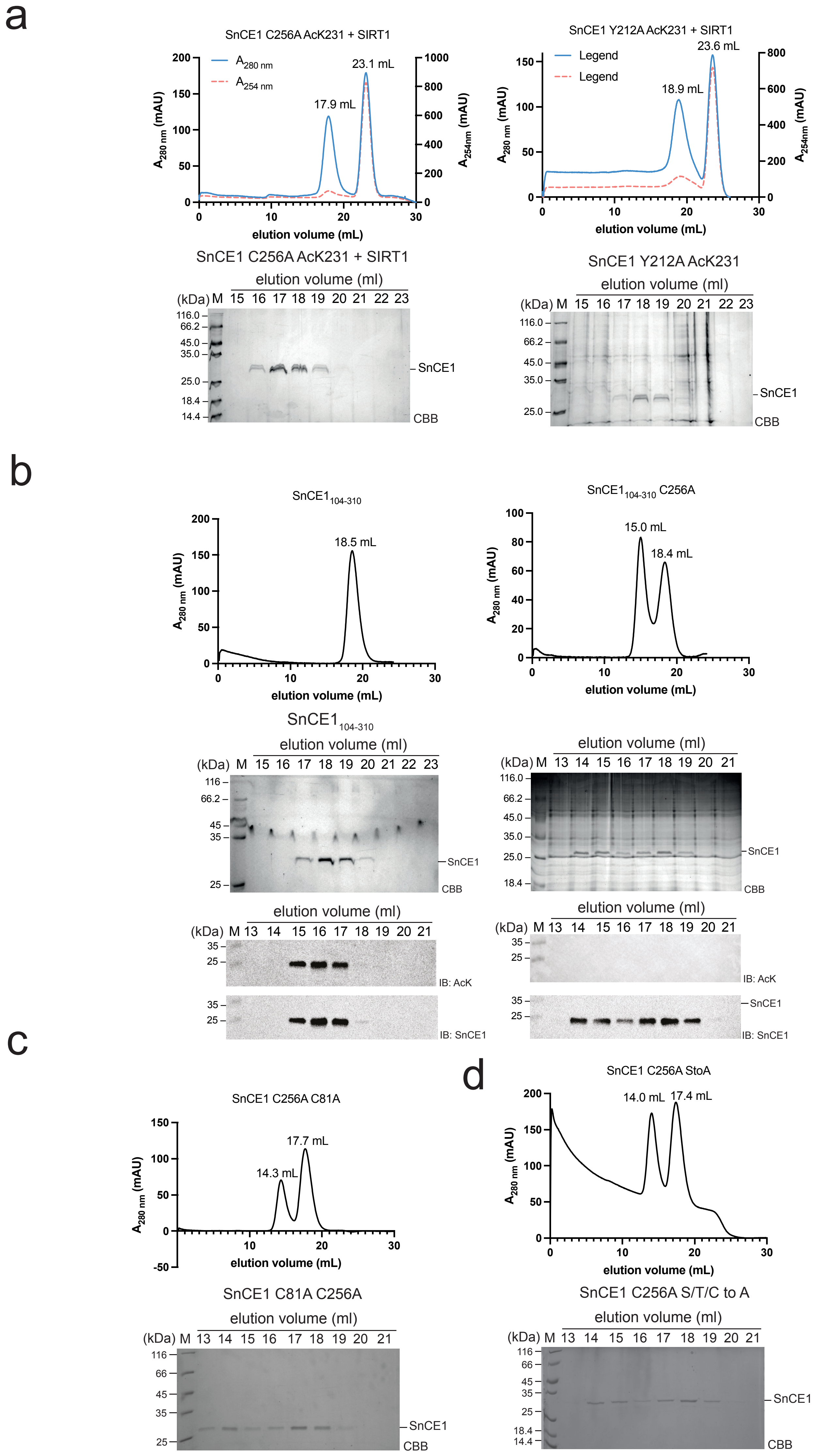
a
SnCE1 C256A AcK231 + SIRT1
A280 nm
A254 nm
17.9 mL
23.1 mL
A280 nm (mAU)
A254nm (mAU)
elution volume (mL)
SnCE1 Y212A AcK231 + SIRT1
Legend
Legend
18.9 mL
23.6 mL
SnCE1 C256A AcK231 + SIRT1
elution volume (ml)
(kDa) M 15 16 17 18 19 20 21 22 23
116.0
66.2
45.0
35.0
25.0
18.4
14.4
SnCE1
CBB
SnCE1 Y212A AcK231
elution volume (ml)
(kDa) M 15 16 17 18 19 20 21 22 23
SnCE1
CBB
b
SnCE1104-310
18.5 mL
SnCE1104-310 C256A
15.0 mL
18.4 mL
elution volume (mL)
SnCE1104-310
elution volume (ml)
(kDa) M 15 16 17 18 19 20 21 22 23
SnCE1
CBB
elution volume (ml)
(kDa) M 13 14 15 16 17 18 19 20 21
SnCE1
CBB
IB: AcK
IB: SnCE1
c
SnCE1 C256A C81A
14.3 mL
17.7 mL
d
SnCE1 C256A StoA
14.0 mL
17.4 mL
SnCE1 C81A C256A
elution volume (ml)
(kDa) M 13 14 15 16 17 18 19 20 21
SnCE1
CBB
SnCE1 C256A S/T/C to A
elution volume (ml)
(kDa) M 13 14 15 16 17 18 19 20 21
SnCE1
CBB

Supplementary Figure 22: Analytical SEC on a S200 10/300 column of deacetylated SnCE1 C256A AcK231 and SnCE1 Y212A AcK231, SnCE1 C256A C81A and SnCE1$_{104\text{-}310}$ and SnCE1$_{104\text{-}310}$ C256A.

a. The treatment of SnCE1 C256A AcK231 and SnCE1 Y212A AcK231 with human SIRT1 in presence of NAD^+ (peak at 23.1 mL; absorbance $A_{254\ nm}$) does not affect its oligomeric state, i.e. both proteins elute as pure monomers. A_{280} is the absorption at 280 nm. mAU: milli absorbance units. Fractions were analysed by SDS-PAGE and gels were stained with Coomassie brilliant blue (CBB). The experiment was performed in two independent technical replicates (n=2). Source data are provided as Source Data file.

b. Analytical size-exclusion chromatography (SEC) of SnCE1$_{104\text{-}310}$ shows that it elutes as monomer and SnCE1$_{104\text{-}310}$ C256A as tetramer. The SDS-PAGE gel shows no truncation due to proteolytic processing suggesting in SnCE1$_{74\text{-}310}$ a truncation from the N-terminus. The elution volumes and calculated molecular weights (calc. MW) were used in comparison to expected molecular weights (exp. MW) to calculate the apparent oligomeric state (Supplementary Fig. 2b). A_{280} is the absorption at 280 nm. mAU: milli absorbance units. SDS-PAGE gels were stained with Coomassie brilliant blue (CBB). Source data are provided as Source Data file.

c. Analytical SEC of SnCE1 C256A/C81A shows it elutes in a tetramer-monomer equilibrium. The elution volumes and calculated molecular weights (calc. MW) were used in comparison to expected molecular weights (exp. MW) to calculate the apparent oligomeric state (Supplementary Fig. 2b). A_{280} is the absorption at 280 nm. mAU: milli absorbance units. The SDS-PAGE gel was stained with Coomassie brilliant blue (CBB). Source data are provided as Source Data file.

d. Analytical SEC of SnCE1 multiple S to A mutant shows it elutes in a tetramer-monomer equilibrium. The elution volumes and calculated molecular weights (calc. MW) were used in comparison to expected molecular weights (exp. MW) to calculate the apparent oligomeric state (Supplementary Fig. 2b). A_{280} is the absorption at 280 nm. mAU: milli absorbance units. The SDS-PAGE gel was stained with Coomassie brilliant blue (CBB). Source data are provided as Source Data file.

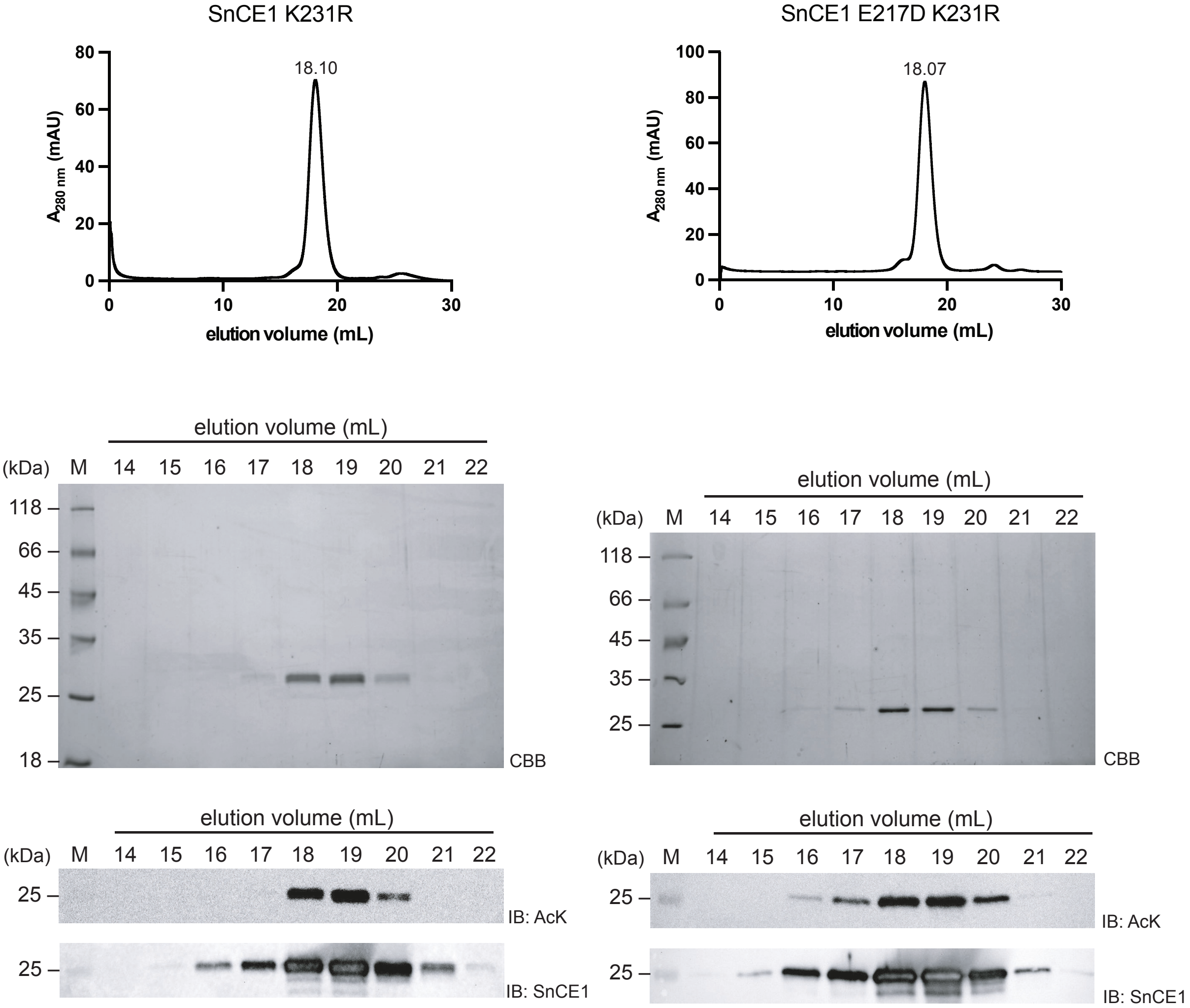

Supplementary Figure 23: SnCE1 K231R and SnCE1 E217D K231R form monomers.
To investigate if tetramer-formation of SnCE1 interferes with its deSUMOylase activity as suggested by the structural data, we prepared the mutants SnCE1 K231R and SnCE1 E217D K231R. These mutants contain the active site nucleophile C256. Analytical size-exclusion chromatography (SEC) on a Superdex 200 Increase 10/300 GL column showed that both mutants elute as pure monomers suggesting that this salt bridge between R231 and D217/E217 is not properly formed for tetramer formation. The absorption at 280 nm ($A_{280\ nm}$) was recorded in mAU (milli absorbance units). SDS-PAGE gels of the fractions of the SEC runs were stained using Coomassie brilliant blue (CBB). The samples from the elutions were analysed by immunoblotting using an anti-acetyl lysine antibody (IB: AcK). The staining with the anti-AcK antibody shows that both mutants were lysine acetylated. Staining with an anti-SnCE1 antibody was done as loading control (IB: SnCE1). 0.1 to 0.2 mg of protein was analysed. The experiment was performed once (*n*=1). Source data are provided as Source Data file.

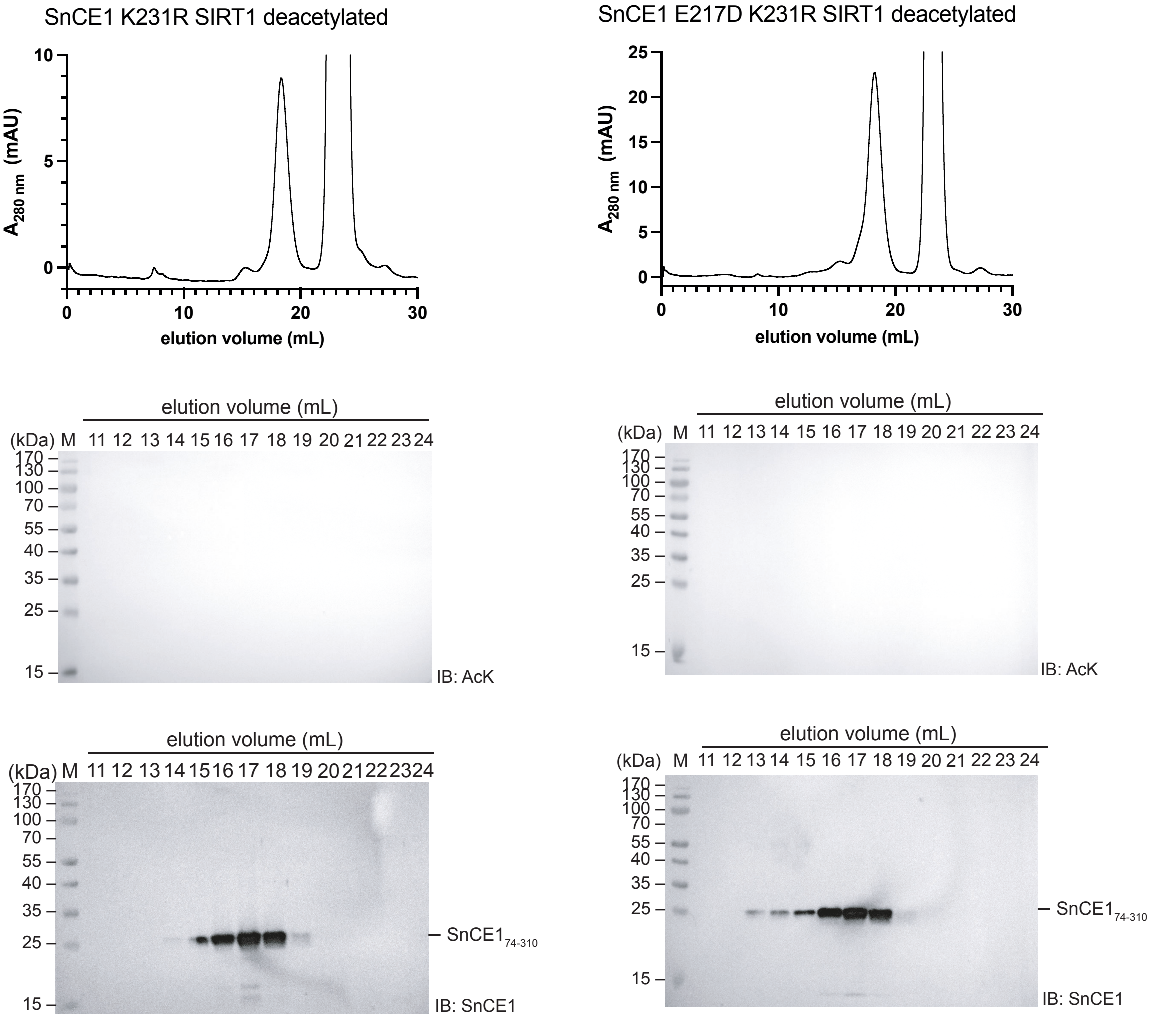

Supplementary Figure 24: Deacetylated SnCE1 K231R and SnCE1 E217D K231R form monomers. To investigate if tetramer-formation of SnCE1 K231R and/or SnCE1 K231R E217D might be prohibited by presence of other lysine acetylation sites in SnCE1, we deacetylated both proteins with SIRT1 and analysed its oligomeric state by analytical size-exclusion chromatography (SEC). Also, upon deacetylation both proteins do not form a tetramer suggesting that the mutants do not allow to reconstitute the correct state of SnCE1 capable to form the tetramer. SDS-PAGE gels of the fractions of the SEC runs were stained using Coomassie brilliant blue (CBB). The samples from the elutions were analysed by immunoblotting using an anti-acetyl lysine antibody (IB: AcK). The staining with the anti-AcK antibody shows that both mutants were lysine acetylated. Staining with an anti-SnCE1 antibody was done as loading control (IB: SnCE1). The $A_{280\ nm}$ peak at 22-25 mL does not contain protein and corresponds to the eluted NAD^+ used as so-substrate for SIRT1-catalysed deacetylation. 0.1 to 0.2 mg of protein was analysed on a Superdex 200 Increase 10/300 GL column. Below the SEC elution profiles showing the absorption at 280 nm ($A_{280\ nm}$) in mAU (milli absorbance units) the SDS-PAGE gels were shown analysing fractions of the observed absorption peaks. The experiment was performed once (*n*=1). Source data are provided as Source Data file.

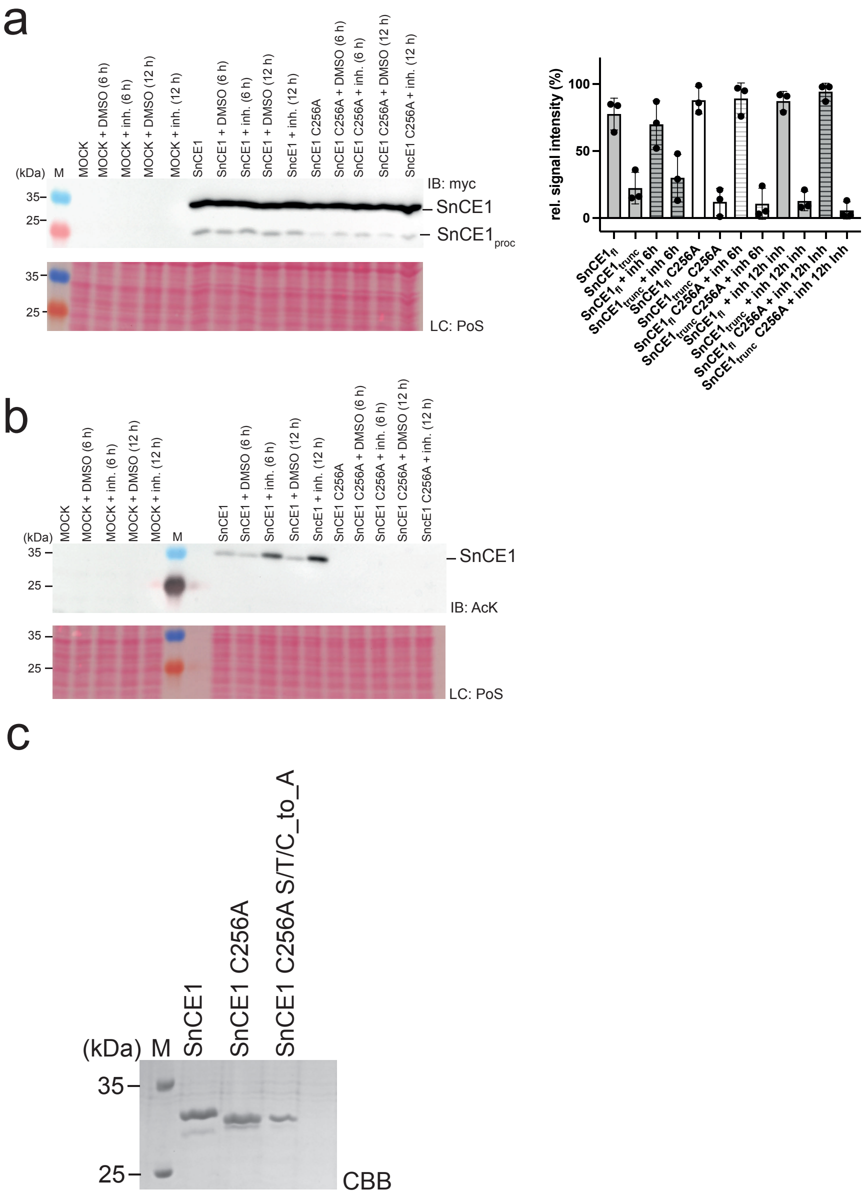

Supplementary Fig. 25: SnCE1 full length is proteolytically processed at the N-terminus in eukaryotic HEK-293 cells and the mutant SnCE1 C256A S/T/CtoA is proteolytically processed.

- **a.** SnCE1 full length ($SnCE1_{f.l.}$) and $SnCE1_{f.l.}$ C256A were transiently expressed in HEK-293 cells as C-terminal myc-His_6 fusion protein. Immunoblotting using an anti-myc-antibody shows the signals representing $SnCE1_{f.l.}$ and the proteolytically processed form were stained suggesting processing occurs from the N-terminus (IB: myc). Ponceau S red staining was done as loading control (LC: PoS). Experiments were performed in three biological replicates and bars depict mean±SD. Statistical significance was tested using unpaired, two-sided t-tests. All comparisons did not show statistical significance. Source data are provided as Source Data file
- **b.** SnCE1 full length ($SnCE1_{f.l.}$) and $SnCE1_{f.l.}$ C256A were transiently expressed in HEK-293 cells as C-terminal myc-His_6 fusion protein as described in a. Immunoblotting using an anti-acetyl-lysine antibody (IB: AcK) shows the acetylation of $SnCE1_{f.l.}$.Treatment with lysine deacetylase inhibitor cocktail, i.e. TSA and SAHA were used to inhibit classical HDACs and nicotinamide (NA) to inhibit sirtuins for 6 h or 12 h as indicated. DMSO was used as control. Ponceau S red staining was done as loading control (LC: PoS). Experiments were performed in three biological replicates and bars depict mean±SD. Source data are provided as Source Data file.
- **c.** The mutant SnCE1 C256A S/T/CtoA, in which several Ser/Thr/Cys residues in the sequence range of Ser74-Ser90, were mutated to Ala, i.e. S74A/T76A/S77A/C81A/ S84A/S86/S87A/S90A, was recombinantly expressed and purified. The protein shows proteolytic processing as SnCE1 C256A indicating the mutations did not affect proteolytic processing. For comparison the non-processed SnCE1 wildtype was loaded on the SDS-PAGE gel. The gel was stained with Coomassie brilliant blue (CBB).

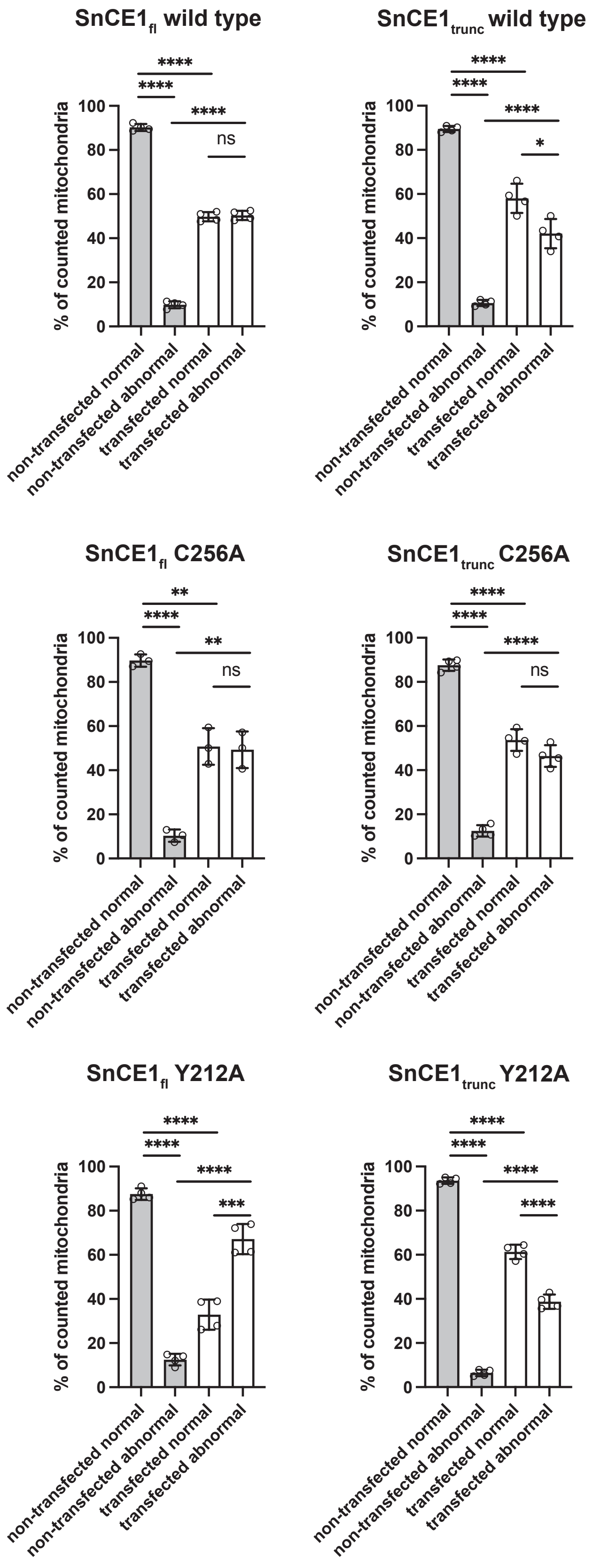

Supplementary Fig. 26: Quantification of the mitochondrial fragmentation observed upon expression of SnCE1 in HEK-293T cells. The fluorescence images shown in Fig. 8c were quantified and the number of non-fragmented and fragmented number was expressed as % of all mitochondria. Experiments were performed in two biological replicates each consisting of two technical replicates (*n*=4). Bars depict mean±SD of determined miller units. Statistical significance (*: $p<0.05$; **: $p \leq 0.01$; ***: $p \leq 0.001$; ****: $p \leq 0.0001$; ns: not significant) was tested using unpaired, two-sided t-tests. Source Data are provided as Source Data file.

a

SnCE1

Predicted localizations: Cytoplasm, Endoplasmic reticulum

Predicted signals:

Localization	Cytoplasm	Nucleus	Extracellular	Cell membrane	Mitochondrion	Plastid	Endoplasmic reticulum	Lysosome/Vacuole	Golgi apparatus	Peroxisome
Probability	0.5856	0.4577	0.1875	0.2335	0.4139	0.0721	0.6866	0.1530	0.3577	0.0384

Probability thresholds for the subcellular localizations. A localization is predicted if its probability is above the threshold shown below:

Localization	Cytoplasm	Nucleus	Extracellular	Cell membrane	Mitochondrion	Plastid	Endoplasmic reticulum	Lysosome/Vacuole	Golgi apparatus	Peroxisome
Threshold	0.4761	0.5014	0.6173	0.5646	0.6220	0.6395	0.6090	0.5848	0.6494	0.7364

b

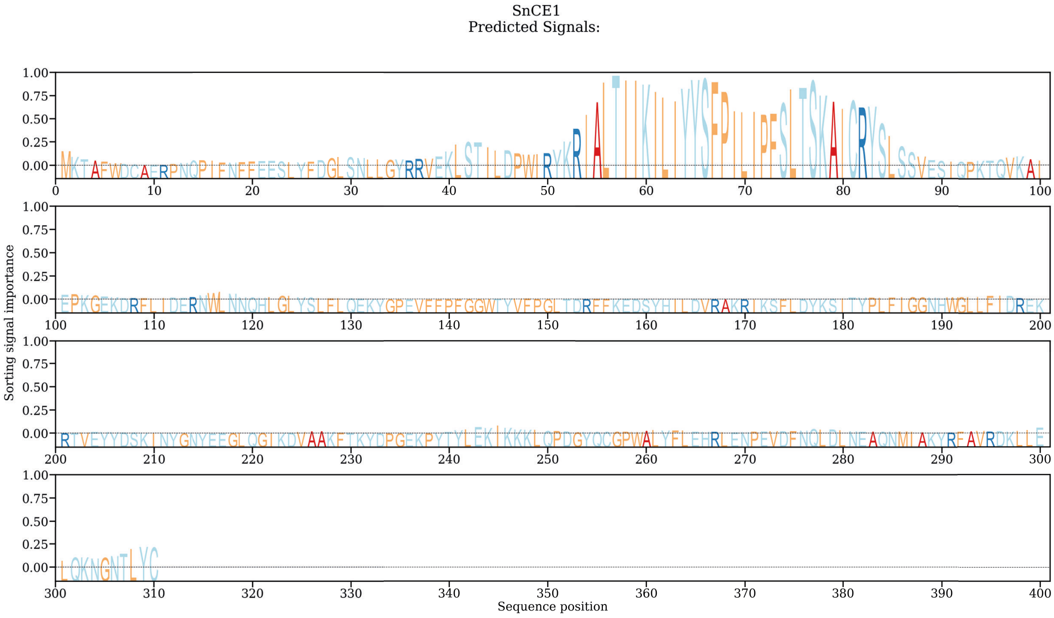

Supplementary Fig. 27: Analyses of SnCE1 regarding its subcellular localization by DeepLoc (https://services.healthtech.dtu.dk/services/DeepLoc-2.0/)[15].

a. The sequence of full length SnCE1 was analysed by DeepLoc. This analysis reveals SnCE1 localizing with high probability to the ER and to the cytosol, whereas localization to the mitochondria is not predicted with high probability. The table at the bottom shows the thresholds indicating a localization to the compartments.

b. Localization to the ER is derived from the presence of a predicted ER localization signa in the amino acid region covering residues 55 to 83 with sorting signal importance scores >50.

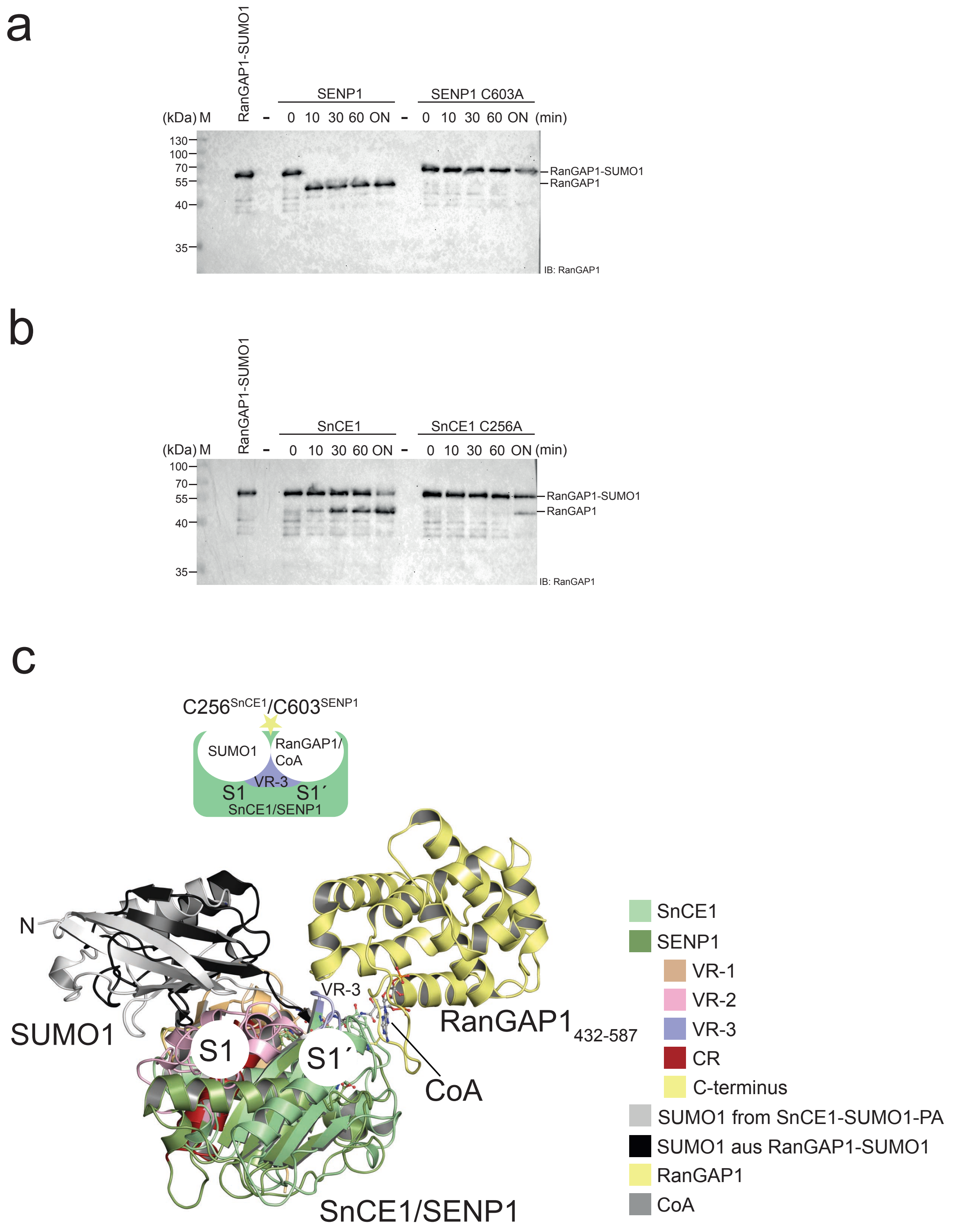

Supplementary Fig. 28: SnCE1 is capable to deSUMOylate RanGAP1 K524-SUMO1.

a. RanGAP1 was mono-SUMO1ylated *in vitro* at K524 and used as substrate for SnCE1 and SnCE1 C256A. The time course experiment shows only SnCE1 wildtype is capable to deSUMOylate RanGAP1 K524-SUMO1.

b. RanGAP1 K524-SUMO1 is deSUMOylated by the deSUMOylase SENP1 but not by catalytically inactive SENP1 C603A.

c. Superposition of the structure of SnCE1-SUMO1-PA (PDB: 9QTG, [https://doi.org/10.2210/pdb9QTG/pdb]) and RanGAP K524-SUMO1•SENP1 (PDB: 2IY0 [https://doi.org/10.2210/pdb2IY0/pdb]).

Supplementary Table 1: Data collection and refinement statistics (molecular replacement) for the structures of *Simkania negevensis* CE1 (SnCE1) wildtype, SnCE1 C256A and SnCE1-SUMO1-PA.

	SnCE1 wildtype[1*] (PDB: 9QTE) [https://doi.org/10.2210/pdb9QTE/pdb])	SnCE1 C256A[2*] (PDB: 9QTF) [https://doi.org/10.2210/pdb9QTF/pdb]	SnCE1-SUMO1-PA (PDB: 9QTG) [https://doi.org/10.2210/pdb9QTG/pdb]
**Data collection**			
Space group	I4	$P12_11$	$P2_12_12_1$
Cell dimensions			
a, *b*, *c* (Å)	105.645, 105.645, 122.086	78.626, 151.173, 80.777	34.967, 56.164, 148.651
α, β, γ (°)	90, 90, 90	90, 105.07, 90	90, 90, 90
Resolution (Å)	47.27 (2.82)[3*]	48.55 (2.20)	49.55 (1.55)
R_{merge}	0.228 (0.941)	0.108 (1.715)	0.117 (1.397)
$I / \sigma I$	8.9 (2.0)	9.7 (0.6)	11.9 (1.7)
Completeness (%)	98.4 (89.1)	96.0 (44.3)	100 (100)
Redundancy	12.9 (7.5)	6.9 (3.5)	12.6 (11.4)
**Refinement**			
Resolution (Å)	2.82	2.20	1.55
No. reflections	205043	608615	551740
R_{work} / R_{free}	0.247 / 0.269	0.155 / 0.191	0.153/0.216
No. atoms			
Protein	5057	13537	2360 (1728 SnCE1, 632 hsSUMO1)
Ligand/ion	14 (MLI)	68 (TRS, EDO)/3	12 (AYE, BME)/1
Water	10	881	268
B-factors			
Protein (main chain/side chain)	63.12 (61.42/64.64)	53.6 (50.7/56.18)	31.05 (28.46/33.38)
Ligand/ion	65.79	63.33/64.54	37.94/25.81
Water	33.34	50.63	37.16
R.m.s. deviations			
Bond lengths (Å)	0.0088	0.0141	0.0166
Bond angles (°)	1.57	1.90	1.92

[1*]/[2*]: for each structure one crystal was used.

[3*]: values in parentheses are for highest-resolution shell.

Supplementary Table 2: Prediction of SUMO-interaction motifs (SIMs) and SUMOylation sites in SnCE1. The online tool GPS-SUMO 2.0 (https://sumo.biocuckoo.cn/index.php) was used to predict potential SIMs and SUMOylation sites in SnCE1. Red: SIMs and lysines predicted tob e SUMOylated.

ID	Position	Peptide	Score	Cut-off	Type
SnCE1	209	TVEYYDSKINYGNYE	0.8946	0.82	SUMOylation
SnCE1	298-302	RFAVRDKLLELQKNGNTLY	0.8608	0.85	SUMO interaction
SnCE1	228	GIKDVAAKFTKYDPG	0.8429	0.82	SUMOylation
SnCE1	231	DVAAKFTKYDPGEKP	0.8327	0.82	SUMOylation
SnCE1	178	IKSFLDYKSITYPLF	0.8233	0.82	SUMOylation

Supplementary Table 3: Primers for site-directed mutagenesis of SnCE1 and Gibson assembly of truncated SnCE1 variants.

Primer	Sequence 5` -> 3`
**Gibson assembly**	
Gib_pOPIN-S_SnCE1_83-310_f	CGCGAACAGATCGGTGGTTATTCACTATCTTCTGTTGAAAG
Gib_pOPIN-S_SnCE1_1-310_r	GGTGTTTAAATGGTCTAGAAAGCTTTAACAATACAAAGTATTTCCATTT TTTTG
Gib_pGEX_SnCE1_fl_r	GTCAGTCACGATGCGGCCGCTCGAGTTAACAATACAGGGTATTGC
Gib_pGEX_SnCE1_95-310_f	TCTGTTCCAGGGGCCCCTGGGATCCACCCAGGTTAAAGCCATTG
Gib_pGEX_SnCE1_104-310_f	TCTGTTCCAGGGGCCCCTGGGATCCGGTGAAAAAGATCGTTTTC
**Site-directed mutagenesis**	
QC_pOPIN-S_SENP1_C603A_f	GCAGATGAATGGAAGTGACGCTGGGATGTTTGCCTGCAAATATG
QC_pOPIN-S_SENP1_C603A_r	CATATTTGCAGGCAAACATCCCAGCGTCACTTCCATTCATCTGC
QC_pGEX_SnCE1_Y146A_f	CGGAAAAACAGCGGTCCAACCG
QC_pGEX_SnCE1_Y146A_r	CGGTTGGACCGCTGTTTTTCCG
QC_pGEX_SnCE1_H190A_f	GTTTATTGGTGGCAATGCTTGGGGCCTG
QC_pGEX_SnCE1_H190A_r	CAGGCCCCAAGCATTGCCACCAATAAAC
QC_pGEX_SnCE1_W191A_f	CGCTGTTTATTGGTGGCAATCATGCGGGCCTGCTGTT
QC_pGEX_SnCE1_W191A_r	AACAGCAGGCCCGCATGATTGCCACCAATAAACAGCG
QC_pGEX_SnCE1_S208A_f	CGCACCGTTGAATATTATGATGCTAAAATTAACTACG
QC_pGEX_SnCE1_S208A_r	CTTCGTAGTTACCGTAGTTAATTTTAGCATCATAATATTCAACG
QC_pGEX_SnCE1_K209Q_f	GTTGAATATTATGATAGTCAAATTAACTACGGTAACTAC
QC_pGEX_SnCE1_K209Q_r	GTAGTTACCGTAGTTAATTTGACTATCATAATATTCAAC
QC_pGEX_SnCE1_Y212A_f	GTAAAATTAACGCCGGTAACTACG
QC_pGEX_SnCE1_Y212A_r	GTTACCGGCGTTAATTTTACTATC
QC_pGEX_SnCE1_K247R_f	CTGGAAAAAATTAAGAGAAAGCTGCAGCC
QC_pGEX_SnCE1_K247R_r	GGCTGCAGCTTTCTCTTAATTTTTTCCAG
QC_pGEX_SnCE1_C256A_f	GATGGTTATCAGGCCGGTCCGTGGGC
QC_pGEX_SnCE1_C256A_r	GCCCACGGACCGGCCTGATAACCATC
QC_pcDNA_SnCE1_Y212A_f	GCAAGATCAATGCCGGCAACTACGAG
QC_pcDNA_SnCE1_Y212A_r	CTCGTAGTTGCCGGCATTGATCTTGC
QC_pcDNA_SnCE1_C256A_f	CGGCTACCAGGCTGGCCCTTG
QC_pcDNA_SnCE1_C256A_r	CAAGGGCCAGCCTGGTAGCCG

Supplementary Table 4: Bacterial and mammalian expression vectors used in this study.

Vector	Insert	Origin/Source/Manufacturer
**Bacterial expression vectors**		
pGEX6P.1	SnCE1 (74 – 310)	BioCat GmbH, Heidelberg
pGEX6P.1	SnCE1 (74 – 310) Y146A	this study
pGEX6P.1	SnCE1 (74 – 310) H190A	this study
pGEX6P.1	SnCE1 (74 – 310) H190A C256A	this study
pGEX6P.1	SnCE1 (74 – 310) W191A	this study
pGEX6P.1	SnCE1 (74 – 310) S208A	this study
pGEX6P.1	SnCE1 (74 – 310) S208A C256A	this study
pGEX6P.1	SnCE1 (74 – 310) K209Q	this study
pGEX6P.1	SnCE1 (74 – 310) Y212A	this study
pGEX6P.1	SnCE1 (74 – 310) K247R	this study
pGEX6P.1	SnCE1 (74 – 310) C256A	this study
pGEX6P.1	SnCE1 (95 – 310)	this study
pGEX6P.1	SnCE1 (95 – 310) C256A	this study
pGEX6P.1	SnCE1 (104 – 310)	this study
pGEX6P.1	SnCE1 (104 – 310) C256A	this study
pOPIN-S	SnCE1 (83 – 310)	this study
pOPIN-S	SnCE2 (144 – 431)	Reference[16]
pOPIN-S	SnCE3 (120 – 431)	Reference[16]
pOPIN-S	SnCE4 (60 – 504)	Reference[16]
pOPIN-S	SnCE5 (1 – 174)	Reference[16]
pOPIN-S	*H. sapiens* SENP1 (415 – 644)	Reference[16]
pET28a(+)	*H. sapiens* SENP6 (627 – 1112)	from Kay Hofmann, University of Cologne
pET28a(+)	*S. cerevisiae* Ulp1 (403 – 621)	From Uwe Bornscheuer, University of Greifswald
pRSF-Duet1	GST-SnCE1 (74 – 310) C81A C256A	BioCat GmbH, Heidelberg
pRSF-Duet1	GST-SnCE1 (74 – 310) AcK78 C256A	BioCat GmbH, Heidelberg
pRSF-Duet1	GST-SnCE1 (74 – 310) AcK94 C256A	BioCat GmbH, Heidelberg
pRSF-Duet1	GST-SnCE1 (74 – 310) AcK98 C256A	BioCat GmbH, Heidelberg
pRSF-Duet1	GST-SnCE1 (74 – 310) AcK103 C256A	BioCat GmbH, Heidelberg
pRSF-Duet1	GST-SnCE1 (74 – 310) AcK106 C256A	BioCat GmbH, Heidelberg
pRSF-Duet1	GST-SnCE1 (74 – 310) AcK209 C256A	BioCat GmbH, Heidelberg
pRSF-Duet1	GST-SnCE1 (74 – 310) AcK231 Y212A	BioCat GmbH, Heidelberg
pRSF-Duet1	GST-SnCE1 (74 – 310) AcK231 C256A	BioCat GmbH, Heidelberg
pRSF-Duet1	GST-SnCE1 (74 – 310) AcK248 C256A	BioCat GmbH, Heidelberg
pRSF-Duet1	GST-SnCE1 (74-310) C256A S/T/C_to_A	BioCat GmbH, Heidelberg
pRSF-Duet1	SnCobB (1 – 262)	BioCat GmbH, Heidelberg
pTXB1	ubiquitin (1 – 75)	Reference:[16]
pTXB1	3xFLAG-SUMO1 (1 – 96)	Reference:[16]
pTXB1	SUMO1 (20 – 96)	BioCat GmbH, Heidelberg
pTXB1	SUMO3 (1 – 91)	Reference:[16]
**Mammalian expression**		
pcDNA3.1(+)	SnCE1 (1 – 310)	BioCat GmbH, Heidelberg
pcDNA3.1(+)	SnCE1 (74 – 310)	BioCat GmbH, Heidelberg
pcDNA3.1(+)	SnCE1 (1 – 310) K78Q C256A	BioCat GmbH, Heidelberg
pcDNA3.1(+)	SnCE1 (1 – 310) C81A C256A	BioCat GmbH, Heidelberg
pcDNA3.1(+)	SnCE1 (1 – 310) S208A	BioCat GmbH, Heidelberg
pcDNA3.1(+)	SnCE1 (1 – 310) S208A C256A	BioCat GmbH, Heidelberg
pcDNA3.1(+)	SnCE1 (1 – 310) Y212A	this study
pcDNA3.1(+)	SnCE1 (74 – 310) Y212A	this study
pcDNA3.1(+)	SnCE1 (1 – 310) C256A	BioCat GmbH, Heidelberg
pcDNA3.1(+)	SnCE1 (74 – 310) C256A	this study
pcDNA3.1(+)	SnCE1 (74-310) C256A S/T/C_to_A	BioCat GmbH, Heidelberg

Supplementary Table 5: Antibodies used in this study.

Manufacturer/Source	Cat no.	Antigen	Clonality	Dilution	Host
**Primary antibodies**					
abcam, Cambridge UK	ab21623	acetyl-L-lysine	polyclonal	1:5000<br>1:10000	rabbit
provided by Prof. Frauke Melchior, Dr. Annette Flotho, Heidelberg University	non-commercial	RanGAP1	polyclonal	1:1000	goat
Davids Biotechnologie, Regensburg Germany	non-commercial	SnCE1	polyclonal	1:50<br>1:100	rabbit
Thermo Fisher	MA5-37627	SUMO2	monoclonal	1:2000	mouse
Santa Cruz	sc-23954	Calnexin	monoclonal	1:100	mouse
Proteintech	16686-1-AP	CKAP4	polyclonal	1:1000	rabbit
Abcam	ab48139	Mitofilin/Mic60	polyclonal	1:1000	rabbit
GeneTex	GTX102150	Rieske FeS	polyclonal	1:500	rabbit
Santa Cruz	sc-5274	Tubulin beta	monoclonal	1:1000	mouse
GeneTex	GTX18184	His	monoclonal	1:1000	mouse
Gramsch laboratories	CM-100	Myc	polyclonal	1:5000	rabbit
Elabscience	E-AB-20007	Myc-Tag	Monoclonal	1:1000	mouse
**Secondary antibodies (HRP-coupled)**					
abcam, Cambridge UK	ab97110	goat IgG H&L	polyclonal	1:1000	donkey
abcam, Cambridge UK	ab6728	mouse IgG H&L	polyclonal	1:2000	rabbit
abcam, Cambridge UK	ab6721	rabbit IgG H&L	polyclonal	1:5000<br>1:10000	goat
Thermo Fisher Scientific	A16066	mouse IgG H&L - HRP	polyclonal	1:3000	goat
Jackson Immunoresearch	111-035-144	rabbit IgG H&L - HRP	polyclonal	1:3000	goat
Thermo Fisher Scientific	A-11001	anti-mouse-IgG H&L-Alexa488	polyclonal	1:100	goat
Thermo Fisher Scientific	A-21422	anti-mouse-IgG H&L-Alexa555	polyclonal	1:100	goat
Jackson Immunoresearch	115-225-146	Anti-mouse-IgG H&L-Cy2	polyclonal	1:100	goat
Cell Signaling	7076S	mouse IgG	polyclonal	1:15.000	horse
Cell Signaling	7074S	rabbit IgG	polyclonal	1:15.000	goat
LI-COR bioscience	925-68072	IRDye 680RD mouse IgG	polyclonal	1:10.000	goat
LI-COR bioscience	925-32213	IRDye 800CW rabbit IgG	polyclonal	1:5.000	goat

Supplementary Table 6: Reversed phase liquid chromatography (RPLC).

TRAP COLUMN	75 µm inner diameter, packed with 3 µm C18 particles (Acclaim PepMap100, Thermo Scientific)
ANALYTICAL COLUMN	Accucore 150-C18, (Thermo Fisher Scientific)<br>25 cm x 75 µm, 2,6 µm C18 particles, 150 Å pore size
BUFFER SYSTEM	binary buffer system consisting of 0.1% acetic acid in HPLC-grade water (buffer A) and 100% ACN in 0.1% acetic acid (buffer B)
FLOW RATE	300 nl/min
GRADIENT	0 min 2% B →<br>2 min 5% B →<br>18 min 25% B →<br>20 min 40% B →<br>22 min 90% B →<br>25 min 90% B →<br>26 min 2% B →<br>29 min 2% B →<br>30 min 2% B
GRADIENT DURATION	30 min
COLUMN OVEN TEMPERATURE	40°C

Supplementary Table 7: Mass spectrometry.

INSTRUMENT	Q Exactive Plus mass spectrometer (Thermo Scientific)
ELECTROSPRAY	Nanospray Flex Ion Source
OPERATION MODE	Data dependent acquisition
METHOD DURATION	30 min
**FULL MS**	
MS SCAN RESOLUTION	70000
AGC TARGET	3e6
MAXIMUM ION INJECTION TIME FOR THE MS SCAN	120
SCAN RANGE	333 to 1650 m/z
POLARITY	positive
SPECTRA DATA TYPE	profile
INTENSITY THRESHOLD	8300
CHARGE STATE	include 2-6
DYNAMIC EXCLUSION	30 s
**DD-MS2**	
NUMBER OF DEPENDENT SCANS	10
RESOLUTION	17,500
MAXIMUM ION INJECTION TIME MODE	120
DATA TYPE	centroid
DYNAMIC EXCLUSION	30 s
MICROSCANS	1
ISOLATION WINDOW	13 m/z overlap
FIRST MASS	100
DISSOCIATION MODE	higher energy collisional dissociation (HCD)
MINIMUM AGC TARGET	1000
APEX TRIGGER	1 to 40 s
HCD NORMALIZED COLLISION ENERGY	27.5

References:

1. Abramson, J. et al. Accurate structure prediction of biomolecular interactions with AlphaFold 3. *Nature* **630**, 493-500 (2024).
2. Jumper, J. & Hassabis, D. Protein structure predictions to atomic accuracy with AlphaFold. *Nat Methods* **19**, 11-12 (2022).
3. Jumper, J. et al. Highly accurate protein structure prediction with AlphaFold. *Nature* **596**, 583-589 (2021).
4. The PyMOL Molecular Graphics System, Version 3.0, Schrödinger, LLC.
5. Jurrus, E. et al. Improvements to the APBS biomolecular solvation software suite. *Protein Sci* **27**, 112-128 (2018).
6. Gouet, P., Robert, X. & Courcelle, E. ESPript/ENDscript: Extracting and rendering sequence and 3D information from atomic structures of proteins. *Nucleic Acids Res* **31**, 3320-3 (2003).
7. Pruneda, J.N. et al. A Chlamydia effector combining deubiquitination and acetylation activities induces Golgi fragmentation. *Nat Microbiol* **3**, 1377-1384 (2018).
8. Mittal, R., Peak-Chew, S.Y., Sade, R.S., Vallis, Y. & McMahon, H.T. The acetyltransferase activity of the bacterial toxin YopJ of Yersinia is activated by eukaryotic host cell inositol hexakisphosphate. *J Biol Chem* **285**, 19927-34 (2010).
9. Labriola, J.M., Zhou, Y. & Nagar, B. Structural Analysis of the Bacterial Effector AvrA Identifies a Critical Helix Involved in Substrate Recognition. *Biochemistry* **57**, 4985-4996 (2018).
10. Zhang, Z.M. et al. Mechanism of host substrate acetylation by a YopJ family effector. *Nat Plants* **3**, 17115 (2017).
11. Emsley, P., Lohkamp, B., Scott, W.G. & Cowtan, K. Features and development of Coot. *Acta Crystallogr D Biol Crystallogr* **66**, 486-501 (2010).
12. Zhang, Y. & Skolnick, J. Scoring function for automated assessment of protein structure template quality. *Proteins* **57**, 702-10 (2004).
13. Laskowski, R.A. & Swindells, M.B. LigPlot+: multiple ligand-protein interaction diagrams for drug discovery. *J Chem Inf Model* **51**, 2778-86 (2011).
14. Wallace, A.C., Laskowski, R.A. & Thornton, J.M. LIGPLOT: a program to generate schematic diagrams of protein-ligand interactions. *Protein Eng* **8**, 127-34 (1995).
15. Thumuluri, V., Almagro Armenteros, J.J., Johansen, A.R., Nielsen, H. & Winther, O. DeepLoc 2.0: multi-label subcellular localization prediction using protein language models. *Nucleic Acids Res* **50**, W228-W234 (2022).
16. Boll, V. et al. Functional and structural diversity in deubiquitinases of the Chlamydia-like bacterium Simkania negevensis. *Nat Commun* **14**, 7335 (2023).
