# Supplementary material for: Reprogramming of bacterial virulence by lysine acetylation
Source: Nat Commun. 2026 Apr 27;17:3859. doi: 10.1038/s41467-026-72244-8 (PMC13125535; doi:10.1038/s41467-026-72244-8)
Supplement: Supplementary file 2 — Description of Additional Supplementary Files [file 41467_2026_72244_MOESM2_ESM.pdf]

Description of Additional Supplementary Files

Supplementary Data 1: AlphaFold3 structure predictions of the catalytic CE-clan protease domains of SnCE1, SnCE2, SnCE3, SnCE4, SnCE5 from *Simkania negevensis* (catalytic domains) in the apo-state and in complex with Coenzyme A (CoA). The predictions were made using AlphaFold3 v3.0.0 (https://github.com/google-deepmind/alphafold3/releases/tag/v3.0.0).

Supplementary Data 2: Proteins identified by mass spectrometry in recombinantly expressed SnCE1 wild type, SnCE1 Y212A and SnCE1 wild type deacetylated with human SIRT1. The data confirms that no protease is present originating from production of the recombinant SnCE1 protein in *Escherichia coli*.

Supplementary Data 3: Spectra obtained for intact mass determination of the recombinantly expressed and purified proteins produced in this study. The proteins were analyzed on an Orbitrap Eclipse mass spectrometer coupled to an UltiMate 3000 nHPLC (both Thermo Scientific). Detection was done using an Orbitrap or ion trap detector. In cases the expected molecular weights differ by +76 Da, +152 Da or +228 Da covalent adducts were formed with b-mercaptoethanol. A single acetylation of a lysine side chain results in a mass shift of +42 Da, multiples thereof several acetylations. The numbering of samples as shown in Supplementary Data 4.

Supplementary Data 4: Determination of the intact masses of the recombinantly expressed and purified proteins produced in this study. The obtained masses were correlated with modified protein species. In cases the expected molecular weights differ by +76 Da, +152 Da or +228 Da covalent adducts were formed with b-mercaptoethanol. A single acetylation of a lysine side chain results in a mass shift of +42 Da, several acetylations by multiples of +42 Da.
