# Supplementary material for: Reprogramming of bacterial virulence by lysine acetylation
Source: Nat Commun. 2026 Apr 27;17:3859. doi: 10.1038/s41467-026-72244-8 (PMC13125535; doi:10.1038/s41467-026-72244-8)
Supplement: Supplementary file 5 — Supplementary Data 3 [file 41467_2026_72244_MOESM5_ESM.zip › Supplementary_Data_3/7_SnCE1_74-310_Y212A_4713_07_4173_SUMUP_RE_01152026_154805.pdf]

BioPharma Finder Report

Created: 15/01/2026 15:51:58

Sample Information	
Raw File Name	D:\Data\4713\4713_07.raw
Instrument Method	C:\Xcalibur\methods\UltiMate\NoFAIMS_Intact_Protein\Direct_Injection_MS1_IT_7K_RF60_35min.meth
Vial	RA7
Injection Volume (µL)	1
Sample Weight	0
Sample Volume (µL)	0
ISTD Amount	0
Dil Factor	1

Chromatogram Parameters	
Use Restricted Time	True
Time Limits	15.000 - 24.984 minutes
Scan Range	558 - 930
m/z Range	600 - 2000
Chromatogram Trace Type	TIC
Sensitivity	High
Rel. Intensity Threshold (%)	5

Chromatogram

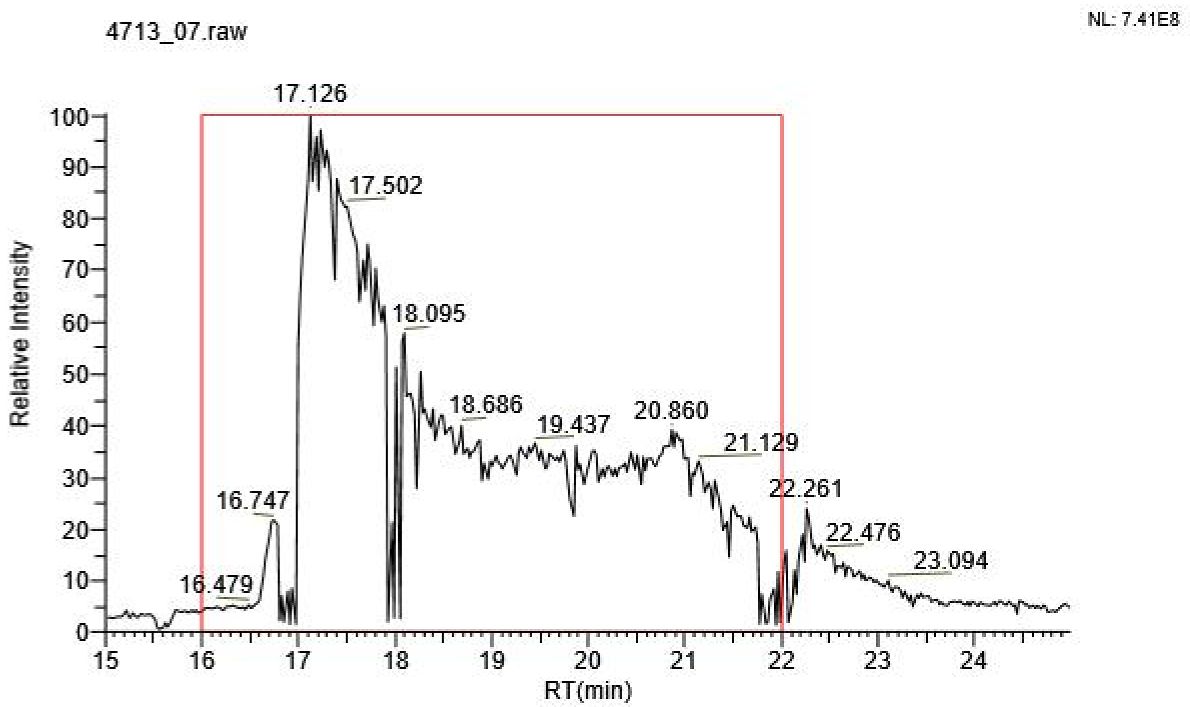

Copyright© 2011 - 2017 Thermo Fisher Scientific

Main Parameters (ReSpect™)	
Deconvolution Results Filter	
Output Mass Range	22500 - 35000
Deconvoluted Spectra Display Mode	Isotopic Profile (new)
Charge State Distribution	
Deconvolution Mass Tolerance	30 ppm
Choice of Peak Model	
Choice of Peak Model	Intact Protein
Resolution at 400 m/z	
Raw File Specific	2000
Generate XIC for Each Component	
Calculate XIC	True

Advanced Parameters (ReSpect™)	
Charge State Distribution	
Model Mass Range	8000 - 70000
Charge State Range	7 - 100
Minimum Adjacent Charges (low & high model mass)	4 - 4
Noise Parameters	
Rel. Abundance Threshold (%)	0
Deconvolution Quality	
Quality Score Threshold	0
Choice of Peak Model	
Target Mass	28000 Da
Peak Model Parameters	
Number of Peak Models	1
Left/Right Peak Shape	2:2
Peak Filter Parameters	
Peak Detection Minimum Significance Measure	1 Standard Deviations
Peak Detection Quality Measure	95%
Specialized Parameters	
Peak Model Width Factor	1
Intensity Threshold Scale	0.01
Deconvolution Parameters	
Noise Compensation	True
Charge Carrier	H
Negative Charge	False

Source Spectra Parameters	
Source Spectra Method	Average Over Selected Retention Time
RT Range	16.000 - 22.000 minutes

Copyright© 2011 - 2017 Thermo Fisher Scientific

Source Spectrum

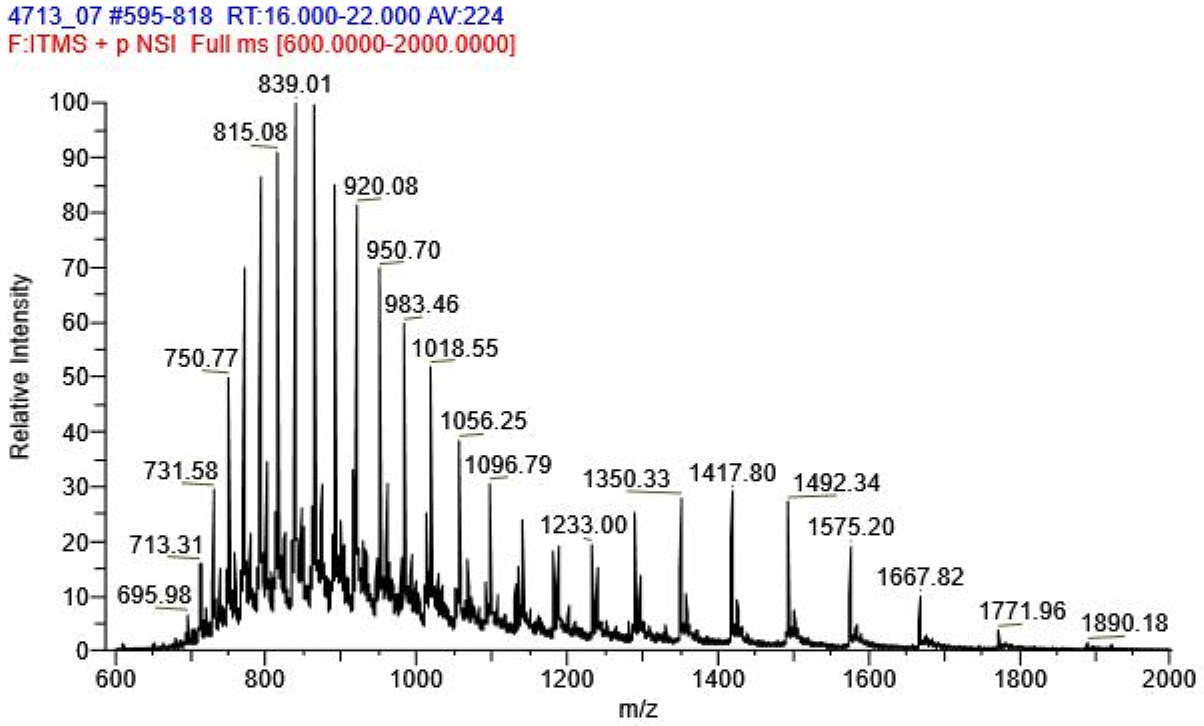

Copyright© 2011 - 2017 Thermo Fisher Scientific

01/15/2026 03:52:00

Deconvoluted Spectrum

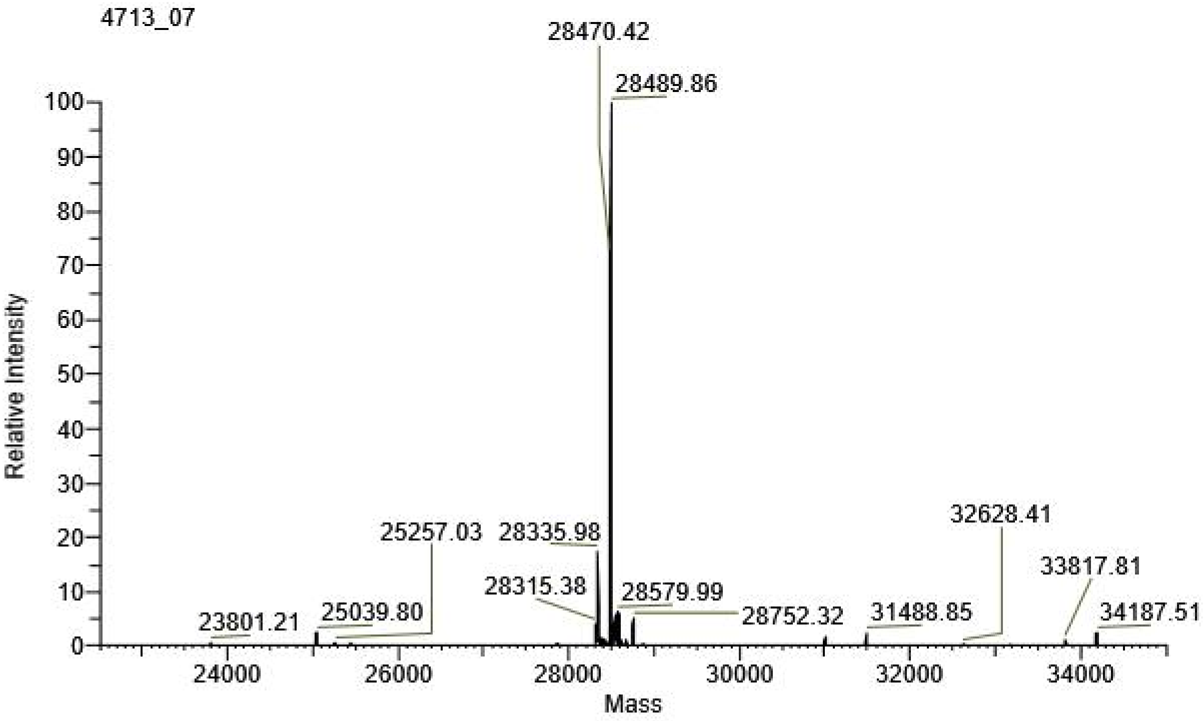

Copyright© 2011 - 2017 Thermo Fisher Scientific

01/15/2026 03:52:00

ReSpect Masses Table													
Row Number	Average Mass	Intensity	Relative Abundance	Fractional Abundance	Score	Number of Charge States	Charge State Distribution	Mass Std Dev	PPM Std Dev	Delta Mass	Start Time (min)	Stop Time (min)	Apex RT
1	28489.86	30312020.00	100.00	59.28	120.78	28	15 - 42	0.48	16.86	0.00	16.000	22.000	17.130
2	28335.98	5249046.00	17.32	10.27	56.66	12	15 - 26	0.54	19.18	-153.88	16.000	22.000	17.230
3	28579.99	1834744.38	6.05	3.59	50.67	9	28 - 36	3.30	115.58	90.13	16.000	22.000	17.130
4	28539.13	1542404.75	5.09	3.02	31.18	6	28 - 33	2.43	85.16	49.27	16.000	22.000	17.180
5	28550.27	1389568.50	4.58	2.72	21.94	4	35 - 38	1.48	51.95	60.42	16.000	22.000	17.230
6	28752.32	1286106.25	4.24	2.52	25.66	5	33 - 37	2.40	83.38	262.46	16.000	22.000	17.230
7	28315.38	1192783.50	3.94	2.33	46.02	11	15 - 25	0.93	32.98	-174.47	16.000	22.000	17.230
8	34187.51	705930.50	2.33	1.38	19.96	4	41 - 44	2.60	76.16	5697.65	16.000	22.000	17.130
9	31488.85	695664.81	2.30	1.36	20.97	4	41 - 44	2.55	81.10	2998.99	16.000	22.000	17.070
10	25039.80	693828.88	2.29	1.36	20.07	4	25 - 28	2.58	103.21	-3450.06	16.000	22.000	17.450
11	28348.46	619358.94	2.04	1.21	21.46	4	28 - 31	2.06	72.68	-141.40	16.000	22.000	17.020
12	28470.42	505544.72	1.67	0.99	52.96	11	15 - 25	1.30	45.72	-19.44	16.000	22.000	17.130
13	31005.82	480723.31	1.59	0.94	17.92	4	39 - 42	1.94	62.45	2515.96	16.000	22.000	17.180
14	28373.75	424977.03	1.40	0.83	38.38	7	19 - 25	1.35	47.43	-116.11	16.000	22.000	17.500
15	28411.00	370877.50	1.22	0.73	99.77	18	16 - 33	3.31	116.50	-78.86	16.000	22.000	17.310
16	28756.10	335339.13	1.11	0.66	84.68	15	16 - 30	1.87	64.90	266.24	16.000	22.000	18.690
17	28667.43	332149.34	1.10	0.65	27.93	5	27 - 31	1.68	58.54	177.58	16.000	22.000	17.130
18	33817.81	321747.38	1.06	0.63	27.03	5	31 - 35	1.44	42.52	5327.96	16.000	22.000	17.180
19	28532.91	320498.38	1.06	0.63	19.40	4	25 - 28	2.71	94.85	43.05	16.000	22.000	17.180
20	28622.05	305683.72	1.01	0.60	26.00	6	29 - 34	2.38	83.20	132.19	16.000	22.000	17.130
21	28602.01	298816.78	0.99	0.58	48.34	9	15 - 23	1.40	48.92	112.16	16.000	22.000	17.500
22	28430.30	273065.56	0.90	0.53	42.34	8	15 - 22	1.65	58.03	-59.56	16.000	22.000	17.260
23	28526.92	272581.09	0.90	0.53	20.21	4	22 - 25	3.39	118.77	37.06	16.000	22.000	17.130
24	28515.70	237065.69	0.78	0.46	17.68	7	15 - 21	1.95	68.52	25.84	16.000	22.000	17.420
25	28450.06	175025.80	0.58	0.34	14.95	4	28 - 31	1.56	54.75	-39.79	16.000	22.000	17.130
26	23801.21	156613.64	0.52	0.31	17.89	5	18 - 22	1.88	79.13	-4688.65	16.000	22.000	17.180
27	25257.03	147573.50	0.49	0.29	21.86	5	32 - 36	1.62	64.17	-3232.83	16.000	22.000	17.260
28	25438.28	146998.88	0.48	0.29	23.50	5	20 - 24	2.24	88.08	-3051.58	16.000	22.000	17.500
29	27861.31	118248.21	0.39	0.23	19.69	4	21 - 24	2.14	76.82	-628.55	16.000	22.000	17.370
30	28582.67	80513.48	0.27	0.16	26.40	5	16 - 20	2.01	70.18	92.81	16.000	22.000	17.180
31	28869.68	72313.16	0.24	0.14	23.18	5	17 - 21	2.84	98.53	379.82	16.000	22.000	18.580
32	25035.14	55882.78	0.18	0.11	20.07	4	17 - 20	2.06	82.46	-3454.71	16.000	22.000	17.130
33	32628.41	54510.69	0.18	0.11	14.51	4	21 - 24	2.40	73.59	4138.56	16.000	22.000	17.230
34	33166.98	52118.55	0.17	0.10	13.07	4	23 - 26	1.80	54.24	4677.12	16.000	22.000	17.130
35	28380.40	39533.55	0.13	0.08	7.04	4	15 - 18	2.01	70.92	-109.46	16.000	22.000	17.290
36	28735.37	30472.06	0.10	0.06	11.07	4	17 - 20	1.64	57.01	245.51	16.000	22.000	17.150

Copyright© 2011 - 2017 Thermo Fisher Scientific
