# Supplementary material for: Reprogramming of bacterial virulence by lysine acetylation
Source: Nat Commun. 2026 Apr 27;17:3859. doi: 10.1038/s41467-026-72244-8 (PMC13125535; doi:10.1038/s41467-026-72244-8)
Supplement: Supplementary file 5 — Supplementary Data 3 [file 41467_2026_72244_MOESM5_ESM.zip › Supplementary_Data_3/6_SnCE1_74-310_K209Q_4713_06_4173_SUMUP_RE_01152026_154804.pdf]

BioPharma Finder Report

Created: 15/01/2026 15:51:23

Sample Information	
Raw File Name	D:\Data\4713\4713_06.raw
Instrument Method	C:\Xcalibur\methods\UltiMate\NoFAIMS_Intact_Protein\Direct_Injection_MS1_IT_7K_RF60_35min.meth
Vial	RA6
Injection Volume (µL)	1
Sample Weight	0
Sample Volume (µL)	0
ISTD Amount	0
Dil Factor	1

Chromatogram Parameters	
Use Restricted Time	True
Time Limits	15.000 - 24.984 minutes
Scan Range	558 - 930
m/z Range	600 - 2000
Chromatogram Trace Type	TIC
Sensitivity	High
Rel. Intensity Threshold (%)	5

Chromatogram

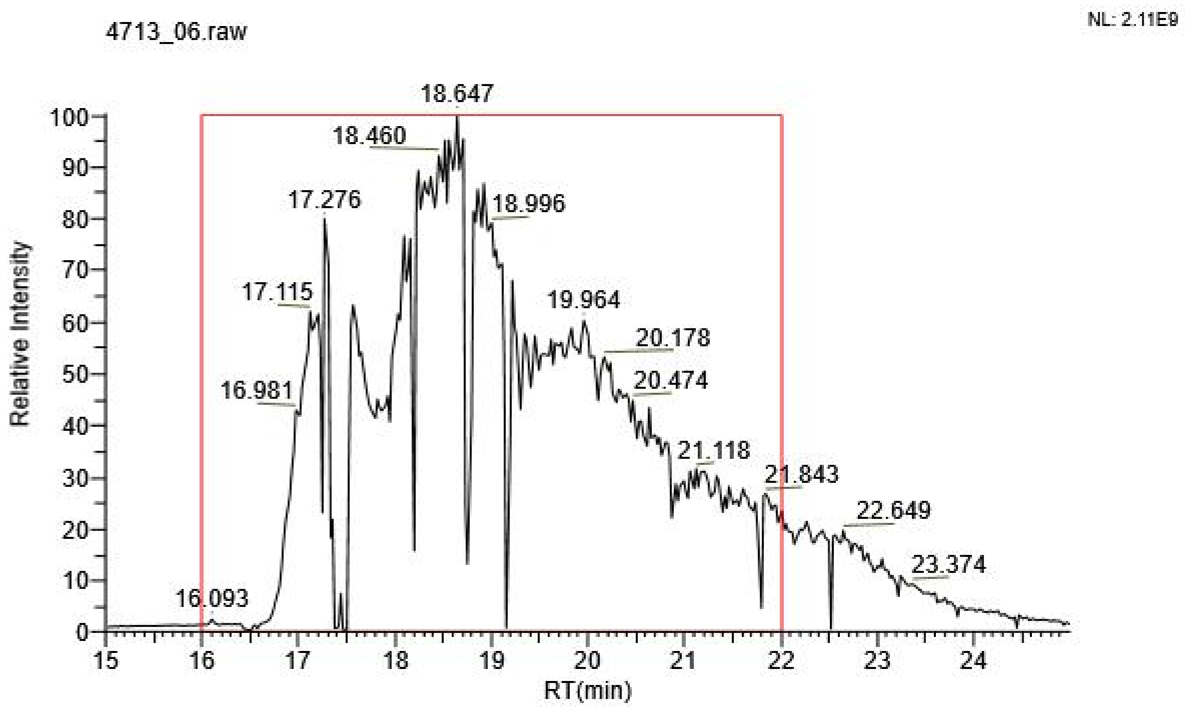

Copyright© 2011 - 2017 Thermo Fisher Scientific

01/15/2026 03:51:25

Main Parameters (ReSpect™)	
Deconvolution Results Filter	
Output Mass Range	22500 - 35000
Deconvoluted Spectra Display Mode	Isotopic Profile (new)
Charge State Distribution	
Deconvolution Mass Tolerance	30 ppm
Choice of Peak Model	
Choice of Peak Model	Intact Protein
Resolution at 400 m/z	
Raw File Specific	2000
Generate XIC for Each Component	
Calculate XIC	True

Advanced Parameters (ReSpect™)	
Charge State Distribution	
Model Mass Range	8000 - 70000
Charge State Range	7 - 100
Minimum Adjacent Charges (low & high model mass)	4 - 4
Noise Parameters	
Rel. Abundance Threshold (%)	0
Deconvolution Quality	
Quality Score Threshold	0
Choice of Peak Model	
Target Mass	28000 Da
Peak Model Parameters	
Number of Peak Models	1
Left/Right Peak Shape	2:2
Peak Filter Parameters	
Peak Detection Minimum Significance Measure	1 Standard Deviations
Peak Detection Quality Measure	95%
Specialized Parameters	
Peak Model Width Factor	1
Intensity Threshold Scale	0.01
Deconvolution Parameters	
Noise Compensation	True
Charge Carrier	H
Negative Charge	False

Source Spectra Parameters	
Source Spectra Method	Average Over Selected Retention Time
RT Range	16.000 - 22.000 minutes

Copyright© 2011 - 2017 Thermo Fisher Scientific

Source Spectrum

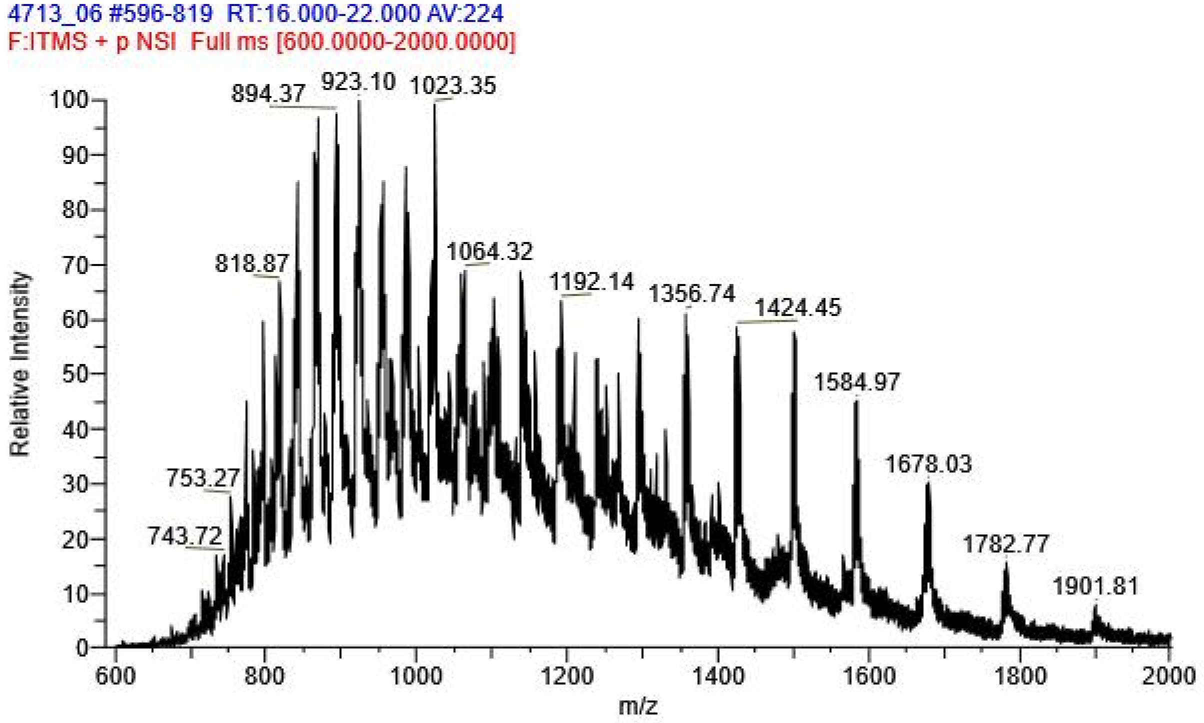

Copyright© 2011 - 2017 Thermo Fisher Scientific

Deconvoluted Spectrum

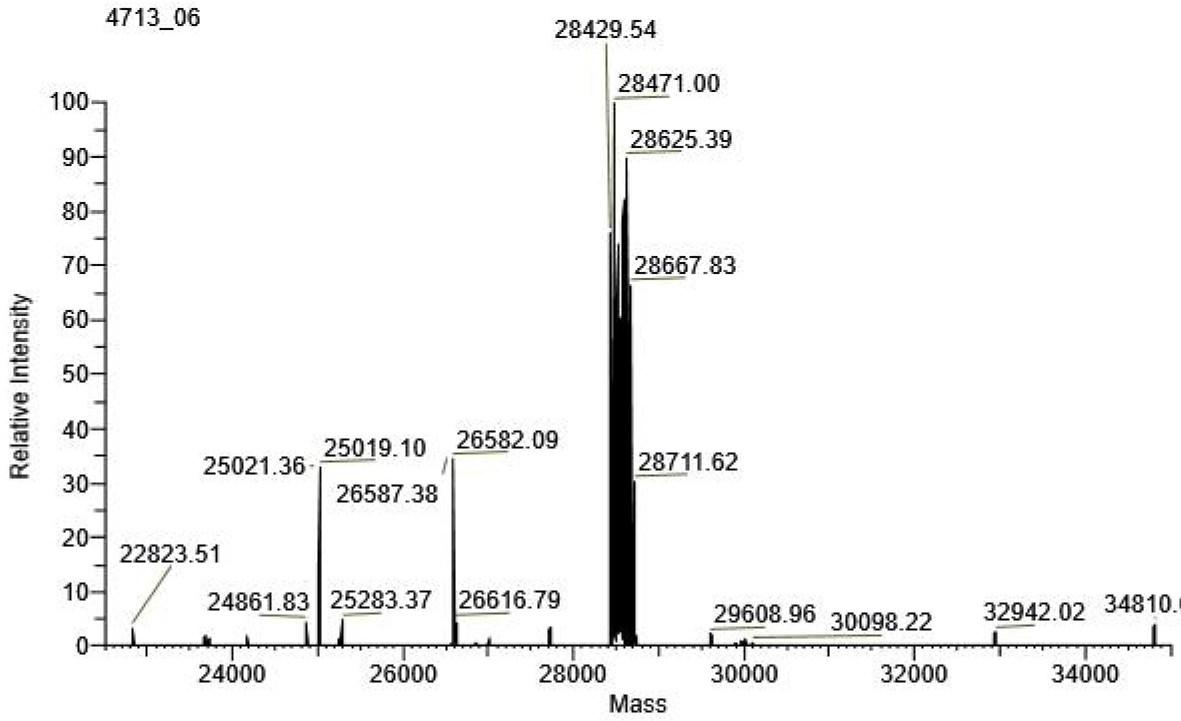

Copyright© 2011 - 2017 Thermo Fisher Scientific

ReSpect Masses Table													
Row Number	Average Mass	Intensity	Relative Abundance	Fractional Abundance	Score	Number of Charge States	Charge State Distribution	Mass Std Dev	PPM Std Dev	Delta Mass	Start Time (min)	Stop Time (min)	Apex RT
1	28471.00	21250424.00	100.00	13.82	114.12	22	15 - 36	2.07	72.86	0.00	16.000	22.000	17.280
2	28625.39	19061230.00	89.70	12.39	98.34	20	19 - 38	1.79	62.47	154.39	16.000	22.000	17.280
3	28429.54	16125466.00	75.88	10.48	120.10	23	15 - 37	2.20	77.26	-41.45	16.000	22.000	17.280
4	28667.83	13609658.00	64.04	8.85	77.25	14	23 - 36	1.36	47.28	196.84	16.000	22.000	17.280
5	28584.62	10837456.00	51.00	7.05	46.15	8	25 - 32	1.47	51.26	113.63	16.000	22.000	17.280
6	28553.80	9155273.00	43.08	5.95	80.50	15	15 - 29	1.43	50.22	82.81	16.000	22.000	17.280
7	28511.55	8288734.00	39.01	5.39	56.67	11	15 - 25	0.97	33.94	40.55	16.000	22.000	17.570
8	28514.22	8150280.50	38.35	5.30	40.65	7	29 - 35	2.04	71.70	43.22	16.000	22.000	17.280
9	28581.69	7302073.50	34.36	4.75	32.69	7	33 - 39	1.03	36.20	110.69	16.000	22.000	17.280
10	28711.62	6414270.50	30.18	4.17	52.91	11	24 - 34	3.26	113.38	240.62	16.000	22.000	17.280
11	26582.09	6135488.00	28.87	3.99	56.25	13	16 - 28	1.93	72.42	-1888.90	16.000	22.000	18.940
12	25019.10	5729675.50	26.96	3.73	56.00	13	14 - 26	2.15	85.75	-3451.90	16.000	22.000	18.920
13	28550.28	4509064.00	21.22	2.93	26.90	5	29 - 33	0.65	22.82	79.28	16.000	22.000	17.280
14	26587.38	2301086.00	10.83	1.50	39.76	8	30 - 37	1.35	50.86	-1883.62	16.000	22.000	18.510
15	25021.36	1463280.50	6.89	0.95	23.24	5	29 - 33	1.74	69.63	-3449.63	16.000	22.000	18.650
16	28595.37	1251077.38	5.89	0.81	26.98	6	15 - 20	1.42	49.48	124.37	16.000	22.000	17.650
17	25283.37	1016749.88	4.78	0.66	23.24	6	16 - 21	1.99	78.68	-3187.63	16.000	22.000	19.000
18	26616.79	969048.69	4.56	0.63	22.46	4	22 - 25	2.69	101.11	-1854.21	16.000	22.000	18.650
19	28534.60	912647.25	4.29	0.59	29.85	7	15 - 21	1.52	53.26	63.60	16.000	22.000	17.570
20	24861.83	903636.94	4.25	0.59	17.37	4	17 - 20	2.48	99.63	-3609.17	16.000	22.000	18.240
21	34810.61	816374.38	3.84	0.53	16.49	4	34 - 37	2.85	81.86	6339.61	16.000	22.000	18.570
22	27722.49	702910.50	3.31	0.46	16.16	4	25 - 28	2.42	87.14	-748.50	16.000	22.000	18.460
23	22823.51	666199.81	3.13	0.43	16.73	4	17 - 20	2.28	100.10	-5647.49	16.000	22.000	18.940
24	28575.70	634599.81	2.99	0.41	20.71	5	15 - 19	2.24	78.40	104.70	16.000	22.000	18.700
25	28664.62	556365.06	2.62	0.36	20.92	4	19 - 22	1.68	58.64	193.62	16.000	22.000	18.240
26	32942.02	521123.13	2.45	0.34	23.62	5	22 - 26	2.70	81.90	4471.02	16.000	22.000	18.920
27	28491.60	490968.91	2.31	0.32	22.30	6	15 - 20	1.17	41.15	20.60	16.000	22.000	17.280
28	29608.96	478436.00	2.25	0.31	18.53	4	26 - 29	1.57	52.92	1137.97	16.000	22.000	18.240
29	23690.08	386290.47	1.82	0.25	10.62	4	22 - 25	2.88	121.74	-4780.91	16.000	22.000	17.280
30	24165.68	371916.06	1.75	0.24	25.07	6	16 - 21	1.69	69.77	-4305.31	16.000	22.000	18.700
31	28736.79	370716.66	1.74	0.24	20.80	4	18 - 21	1.95	67.95	265.79	16.000	22.000	19.350
32	26599.18	303044.72	1.43	0.20	19.29	4	16 - 19	1.51	56.76	-1871.82	16.000	22.000	18.920
33	23669.10	296023.16	1.39	0.19	17.75	4	14 - 17	2.64	111.66	-4801.90	16.000	22.000	18.940
34	27008.99	293683.31	1.38	0.19	21.54	4	16 - 19	2.56	94.88	-1462.01	16.000	22.000	17.570
35	23724.17	273584.22	1.29	0.18	15.14	4	15 - 18	2.25	94.99	-4746.83	16.000	22.000	18.650
36	30010.03	260877.41	1.23	0.17	11.66	4	19 - 22	2.34	77.84	1539.03	16.000	22.000	17.280
37	25250.03	256495.34	1.21	0.17	23.52	5	13 - 17	2.56	101.22	-3220.96	16.000	22.000	18.920
38	28448.31	252458.27	1.19	0.16	19.01	4	16 - 19	0.70	24.75	-22.69	16.000	22.000	17.280
39	29967.15	178432.70	0.84	0.12	15.44	4	20 - 23	1.60	53.32	1496.16	16.000	22.000	17.280
40	26849.28	108475.95	0.51	0.07	15.62	4	17 - 20	1.76	65.61	-1621.71	16.000	22.000	18.650
41	30098.22	100293.77	0.47	0.07	14.42	4	18 - 21	3.01	99.99	1627.22	16.000	22.000	17.550
42	29894.33	92110.55	0.43	0.06	11.36	4	20 - 23	2.47	82.58	1423.33	16.000	22.000	17.280

Copyright© 2011 - 2017 Thermo Fisher Scientific
