# Supplementary material for: Reprogramming of bacterial virulence by lysine acetylation
Source: Nat Commun. 2026 Apr 27;17:3859. doi: 10.1038/s41467-026-72244-8 (PMC13125535; doi:10.1038/s41467-026-72244-8)
Supplement: Supplementary file 5 — Supplementary Data 3 [file 41467_2026_72244_MOESM5_ESM.zip › Supplementary_Data_3/8_SnCE1_74-310_Y212A_AcK231_4713_08_4173_SUMUP_RE_01152026_154806.pdf]

# BioPharma Finder Report

Created: 15/01/2026 15:52:31

| Sample Information | |
|---|---|
| Raw File Name | D:\Data\4713\4713_08.raw |
| Instrument Method | C:\Xcalibur\methods\UltiMate\NoFAIMS_Intact_Protein\Direct_Injection_MS1_IT_7K_RF60_35min.meth |
| Vial | RA8 |
| Injection Volume (µL) | 1 |
| Sample Weight | 0 |
| Sample Volume (µL) | 0 |
| ISTD Amount | 0 |
| Dil Factor | 1 |

| Chromatogram Parameters | |
|---|---|
| Use Restricted Time | True |
| Time Limits | 15.000 - 24.984 minutes |
| Scan Range | 558 - 930 |
| m/z Range | 600 - 2000 |
| Chromatogram Trace Type | TIC |
| Sensitivity | High |
| Rel. Intensity Threshold (%) | 5 |

### Chromatogram

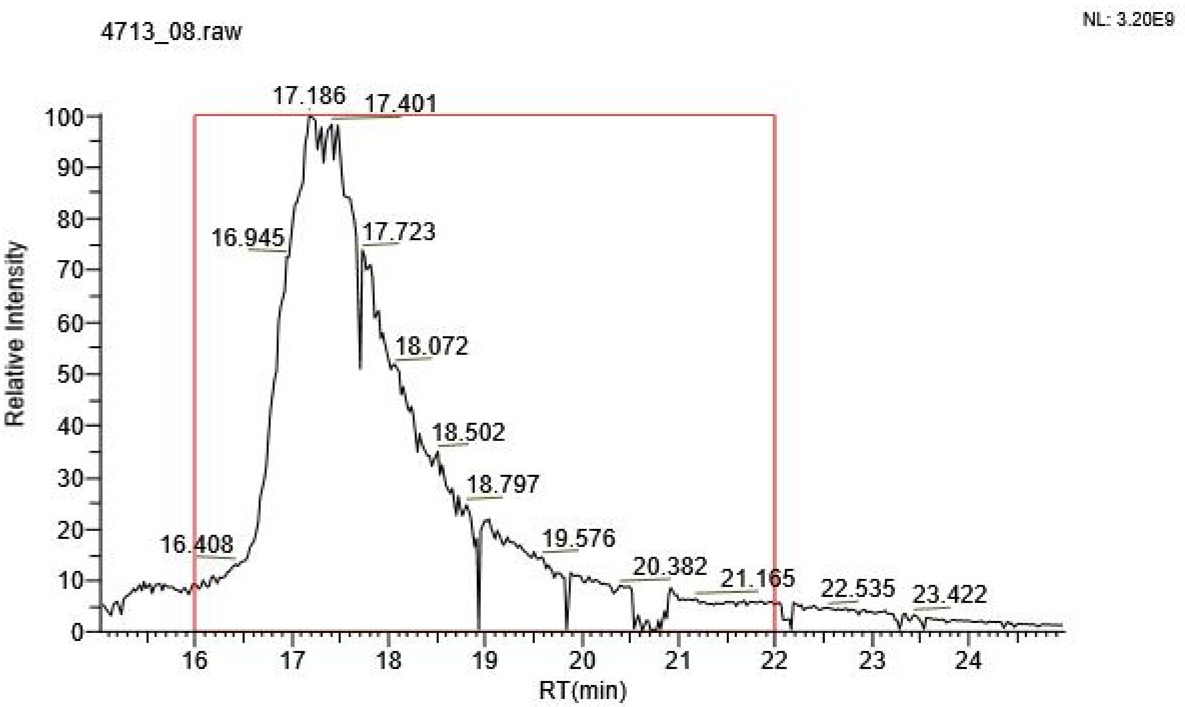



Copyright© 2011 - 2017 Thermo Fisher Scientific

| Main Parameters ( ReSpect™ ) | |
|---|---|
| Deconvolution Results Filter | |
| Output Mass Range | 22500 - 35000 |
| Deconvoluted Spectra Display Mode | Isotopic Profile (new) |
| Charge State Distribution | |
| Deconvolution Mass Tolerance | 30 ppm |
| Choice of Peak Model | |
| Choice of Peak Model | Intact Protein |
| Resolution at 400 m/z | |
| Raw File Specific | 2000 |
| Generate XIC for Each Component | |
| Calculate XIC | True |
| **Advanced Parameters ( ReSpect™)** | |
| Charge State Distribution | |
| Model Mass Range | 8000 - 70000 |
| Charge State Range | 7 - 100 |
| Minimum Adjacent Charges (low & high model mass) | 4 - 4 |
| Noise Parameters | |
| Rel. Abundance Threshold (%) | 0 |
| Deconvolution Quality | |
| Quality Score Threshold | 0 |
| Choice of Peak Model | |
| Target Mass | 28000 Da |
| Peak Model Parameters | |
| Number of Peak Models | 1 |
| Left/Right Peak Shape | 2:2 |
| Peak Filter Parameters | |
| Peak Detection Minimum Significance Measure | 1 Standard Deviations |
| Peak Detection Quality Measure | 95% |
| Specialized Parameters | |
| Peak Model Width Factor | 1 |
| Intensity Threshold Scale | 0.01 |
| Deconvolution Parameters | |
| Noise Compensation | True |
| Charge Carrier | H |
| Negative Charge | False |
| **Source Spectra Parameters** | |
| Source Spectra Method | Average Over Selected Retention Time |
| RT Range | 16.000 - 22.000 minutes |



Copyright© 2011 - 2017 Thermo Fisher Scientific

Source Spectrum

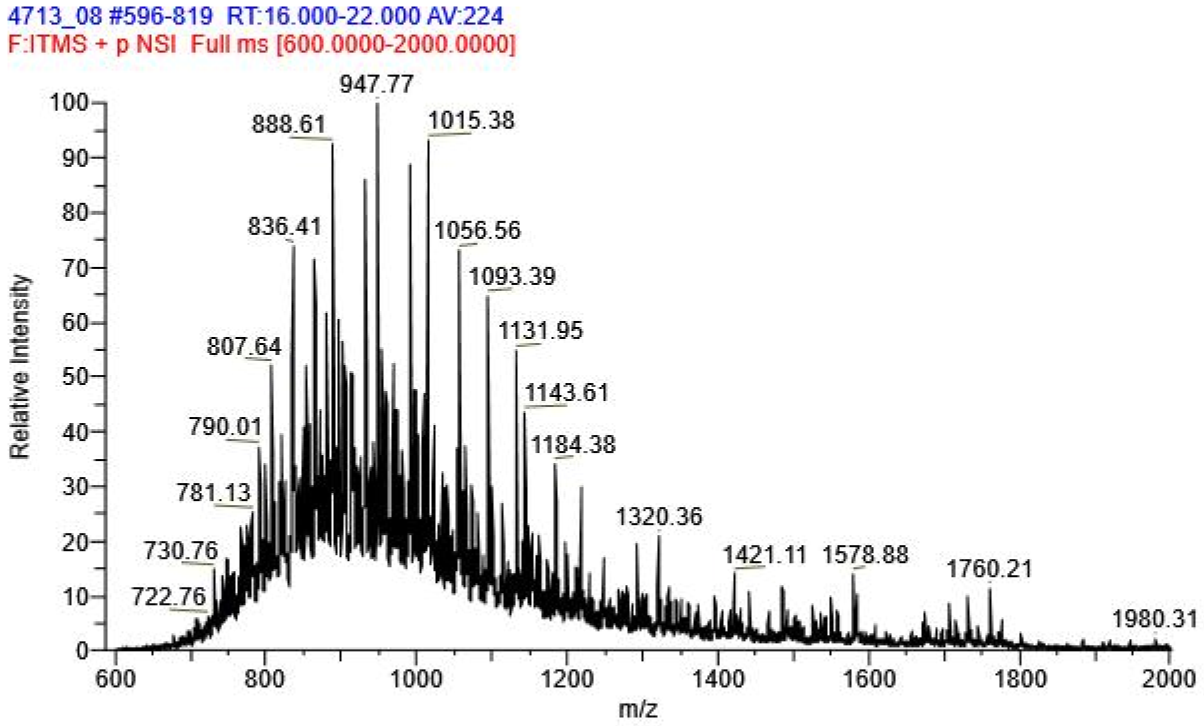




Copyright© 2011 - 2017 Thermo Fisher Scientific

01/15/2026 03:52:34

Deconvoluted Spectrum

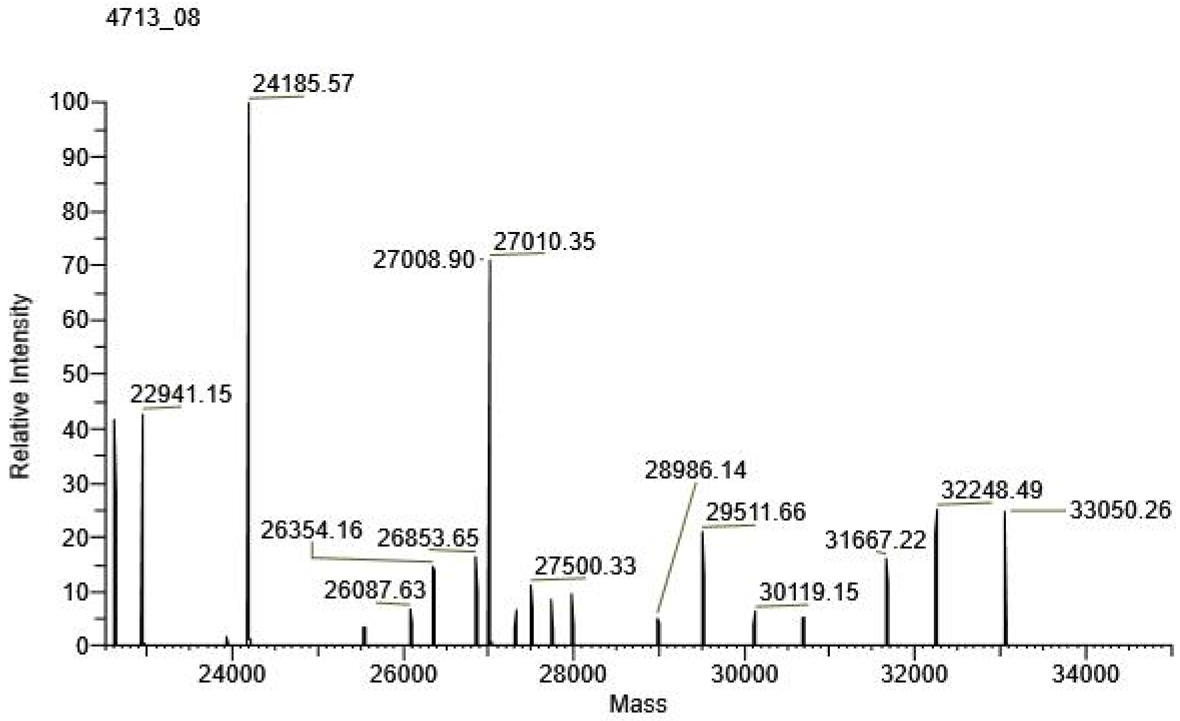



Copyright© 2011 - 2017 Thermo Fisher Scientific
01/15/2026 03:52:34

| ReSpect Masses Table | | | | | | | | | | | | | |
|---|---|---|---|---|---|---|---|---|---|---|---|---|---|
| Row Number | Average Mass | Intensity | Relative Abundance | Fractional Abundance | Score | Number of Charge States | Charge State Distribution | Mass Std Dev | PPM Std Dev | Delta Mass | Start Time (min) | Stop Time (min) | Apex RT |
| 1 | 24185.57 | 7876414.50 | 100.00 | 21.47 | 34.88 | 7 | 19 - 25 | 1.26 | 52.08 | 0.00 | 16.000 | 22.000 | 17.720 |
| 2 | 27010.35 | 5086966.50 | 64.58 | 13.87 | 23.54 | 4 | 28 - 31 | 1.00 | 36.92 | 2824.78 | 16.000 | 22.000 | 17.460 |
| 3 | 22941.15 | 3349989.50 | 42.53 | 9.13 | 16.17 | 4 | 21 - 24 | 1.59 | 69.40 | -1244.42 | 16.000 | 22.000 | 17.240 |
| 4 | 22612.91 | 3270978.00 | 41.53 | 8.92 | 21.42 | 4 | 24 - 27 | 1.92 | 85.11 | -1572.67 | 16.000 | 22.000 | 17.130 |
| 5 | 22624.09 | 2026613.13 | 25.73 | 5.52 | 15.21 | 4 | 29 - 32 | 2.26 | 99.78 | -1561.48 | 16.000 | 22.000 | 17.030 |
| 6 | 32248.49 | 1972378.63 | 25.04 | 5.38 | 21.16 | 4 | 31 - 34 | 1.60 | 49.62 | 8062.92 | 16.000 | 22.000 | 17.480 |
| 7 | 33050.26 | 1943441.50 | 24.67 | 5.30 | 19.50 | 4 | 31 - 34 | 2.46 | 74.49 | 8864.69 | 16.000 | 22.000 | 17.210 |
| 8 | 29511.66 | 1646949.88 | 20.91 | 4.49 | 16.21 | 4 | 29 - 32 | 1.98 | 67.00 | 5326.08 | 16.000 | 22.000 | 17.350 |
| 9 | 26853.65 | 1286400.25 | 16.33 | 3.51 | 27.13 | 5 | 18 - 22 | 1.70 | 63.13 | 2668.08 | 16.000 | 22.000 | 17.370 |
| 10 | 31667.22 | 1261647.25 | 16.02 | 3.44 | 25.31 | 5 | 20 - 24 | 1.47 | 46.37 | 7481.65 | 16.000 | 22.000 | 17.640 |
| 11 | 26354.16 | 1141496.63 | 14.49 | 3.11 | 27.59 | 6 | 19 - 24 | 0.90 | 34.07 | 2168.58 | 16.000 | 22.000 | 17.480 |
| 12 | 27500.33 | 872926.38 | 11.08 | 2.38 | 20.39 | 4 | 21 - 24 | 2.91 | 105.74 | 3314.76 | 16.000 | 22.000 | 17.130 |
| 13 | 27972.02 | 749320.50 | 9.51 | 2.04 | 19.60 | 4 | 23 - 26 | 2.20 | 78.64 | 3786.45 | 16.000 | 22.000 | 17.480 |
| 14 | 27737.08 | 666703.81 | 8.46 | 1.82 | 20.87 | 4 | 22 - 25 | 1.63 | 58.84 | 3551.51 | 16.000 | 22.000 | 17.240 |
| 15 | 26087.63 | 528168.75 | 6.71 | 1.44 | 20.76 | 4 | 17 - 20 | 2.25 | 86.26 | 1902.06 | 16.000 | 22.000 | 17.400 |
| 16 | 27008.90 | 516173.53 | 6.55 | 1.41 | 16.73 | 4 | 20 - 23 | 1.94 | 71.86 | 2823.33 | 16.000 | 22.000 | 17.210 |
| 17 | 27317.74 | 514621.06 | 6.53 | 1.40 | 20.77 | 4 | 19 - 22 | 1.55 | 56.80 | 3132.17 | 16.000 | 22.000 | 17.110 |
| 18 | 30119.15 | 500826.63 | 6.36 | 1.37 | 21.64 | 5 | 16 - 20 | 1.90 | 63.10 | 5933.58 | 16.000 | 22.000 | 17.560 |
| 19 | 30691.04 | 408929.34 | 5.19 | 1.11 | 23.38 | 5 | 22 - 26 | 1.57 | 51.02 | 6505.47 | 16.000 | 22.000 | 17.210 |
| 20 | 28986.14 | 390871.66 | 4.96 | 1.07 | 20.70 | 4 | 19 - 22 | 2.46 | 85.03 | 4800.57 | 16.000 | 22.000 | 17.190 |
| 21 | 27991.22 | 282018.09 | 3.58 | 0.77 | 20.09 | 4 | 18 - 21 | 2.18 | 77.97 | 3805.65 | 16.000 | 22.000 | 17.210 |
| 22 | 25541.28 | 262586.34 | 3.33 | 0.72 | 17.51 | 4 | 14 - 17 | 1.83 | 71.52 | 1355.71 | 16.000 | 22.000 | 17.370 |
| 23 | 23923.74 | 124868.29 | 1.59 | 0.34 | 21.81 | 5 | 16 - 20 | 1.64 | 68.66 | -261.83 | 16.000 | 22.000 | 17.480 |


Copyright© 2011 - 2017 Thermo Fisher Scientific
