# Supplementary material for: Reprogramming of bacterial virulence by lysine acetylation
Source: Nat Commun. 2026 Apr 27;17:3859. doi: 10.1038/s41467-026-72244-8 (PMC13125535; doi:10.1038/s41467-026-72244-8)
Supplement: Supplementary file 5 — Supplementary Data 3 [file 41467_2026_72244_MOESM5_ESM.zip › Supplementary_Data_3/4_SnCE1_74-310_W191A_4713_04_4173_SUMUP_RE_01152026_154802.pdf]

BioPharma Finder Report

Created: 15/01/2026 15:50:10

Sample Information	
Raw File Name	D:\Data\4713\4713_04.raw
Instrument Method	C:\Xcalibur\methods\UltiMate\NoFAIMS_Intact_Protein\Direct_Injection_MS1_IT_7K_RF60_35min.meth
Vial	RA4
Injection Volume (µL)	1
Sample Weight	0
Sample Volume (µL)	0
ISTD Amount	0
Dil Factor	1

Chromatogram Parameters	
Use Restricted Time	True
Time Limits	15.000 - 24.984 minutes
Scan Range	558 - 930
m/z Range	600 - 2000
Chromatogram Trace Type	TIC
Sensitivity	High
Rel. Intensity Threshold (%)	5

Chromatogram

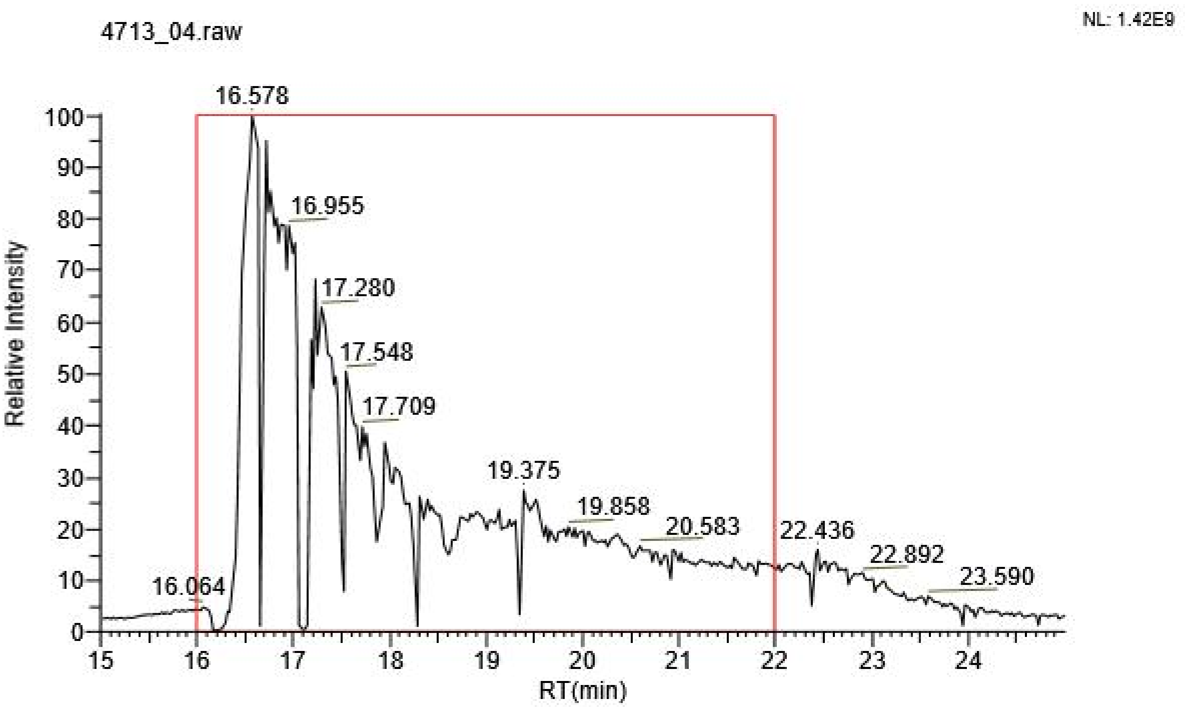

Copyright© 2011 - 2017 Thermo Fisher Scientific

Main Parameters (ReSpect™)	
Deconvolution Results Filter	
Output Mass Range	22500 - 35000
Deconvoluted Spectra Display Mode	Isotopic Profile (new)
Charge State Distribution	
Deconvolution Mass Tolerance	30 ppm
Choice of Peak Model	
Choice of Peak Model	Intact Protein
Resolution at 400 m/z	
Raw File Specific	2000
Generate XIC for Each Component	
Calculate XIC	True

Advanced Parameters (ReSpect™)	
Charge State Distribution	
Model Mass Range	8000 - 70000
Charge State Range	7 - 100
Minimum Adjacent Charges (low & high model mass)	4 - 4
Noise Parameters	
Rel. Abundance Threshold (%)	0
Deconvolution Quality	
Quality Score Threshold	0
Choice of Peak Model	
Target Mass	28000 Da
Peak Model Parameters	
Number of Peak Models	1
Left/Right Peak Shape	2:2
Peak Filter Parameters	
Peak Detection Minimum Significance Measure	1 Standard Deviations
Peak Detection Quality Measure	95%
Specialized Parameters	
Peak Model Width Factor	1
Intensity Threshold Scale	0.01
Deconvolution Parameters	
Noise Compensation	True
Charge Carrier	H
Negative Charge	False

Source Spectra Parameters	
Source Spectra Method	Average Over Selected Retention Time
RT Range	16.000 - 22.000 minutes

Copyright© 2011 - 2017 Thermo Fisher Scientific

Source Spectrum

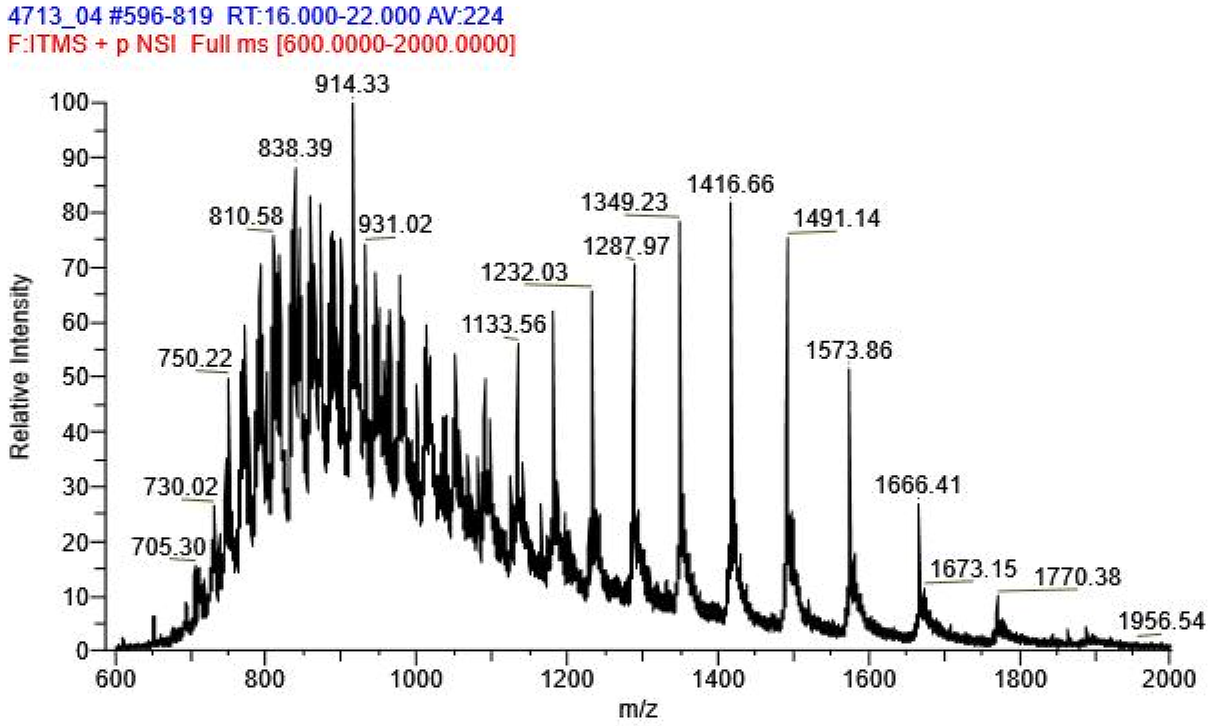

Copyright© 2011 - 2017 Thermo Fisher Scientific

01/15/2026 03:50:14

Deconvoluted Spectrum

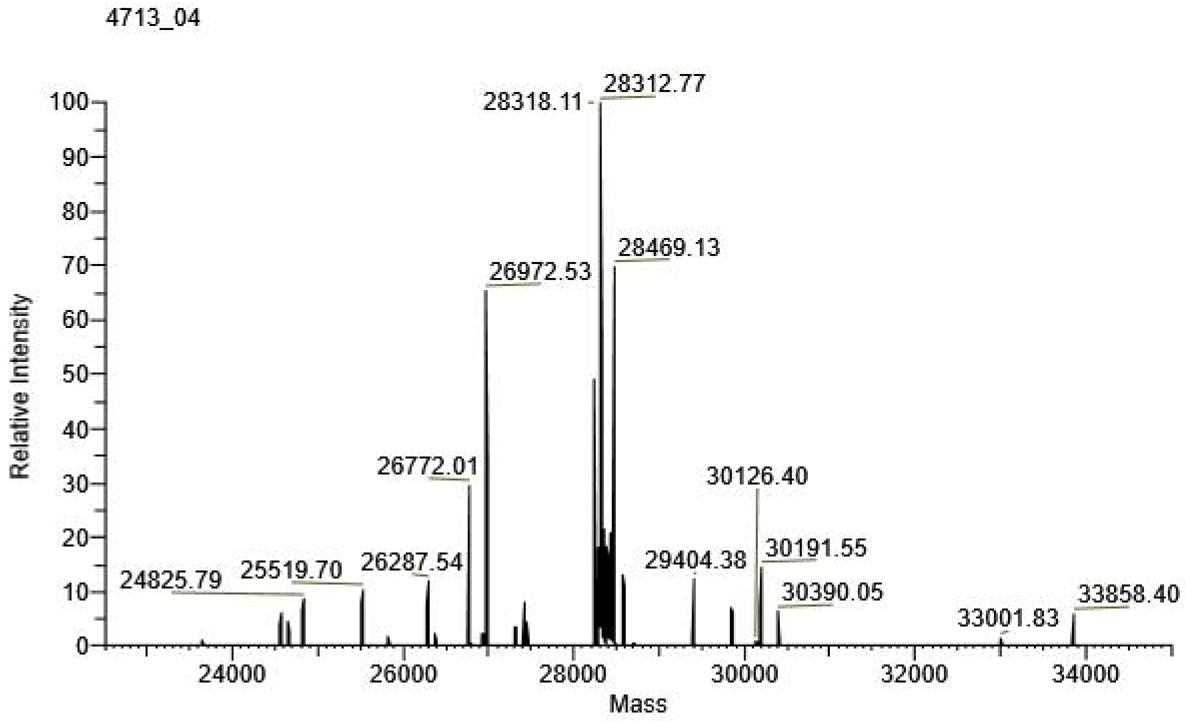

Copyright© 2011 - 2017 Thermo Fisher Scientific

ReSpect Masses Table													
Row Number	Average Mass	Intensity	Relative Abundance	Fractional Abundance	Score	Number of Charge States	Charge State Distribution	Mass Std Dev	PPM Std Dev	Delta Mass	Start Time (min)	Stop Time (min)	Apex RT
1	28312.77	7118643.50	100.00	16.96	59.89	13	15 - 27	0.76	26.78	0.00	16.000	22.000	16.820
2	26972.53	5030143.50	70.66	11.98	65.62	14	23 - 36	1.08	40.07	-1340.24	16.000	22.000	16.950
3	28469.13	3922989.00	55.11	9.35	63.39	13	27 - 39	2.21	77.70	156.36	16.000	22.000	16.580
4	28237.70	3778702.50	53.08	9.00	108.38	23	15 - 37	1.86	65.84	-75.07	16.000	22.000	16.500
5	26772.01	2270634.75	31.90	5.41	49.84	10	24 - 33	2.01	75.01	-1540.76	16.000	22.000	16.580
6	28427.62	1596228.25	22.42	3.80	49.95	11	15 - 25	0.97	34.10	114.85	16.000	22.000	17.280
7	28469.62	1468274.25	20.63	3.50	50.97	11	15 - 25	0.97	34.03	156.85	16.000	22.000	16.580
8	28291.74	1391641.13	19.55	3.32	42.00	10	15 - 24	1.17	41.42	-21.03	16.000	22.000	16.820
9	28318.11	1267963.50	17.81	3.02	24.09	5	29 - 33	1.25	44.08	5.34	16.000	22.000	16.500
10	30191.55	1108358.63	15.57	2.64	23.00	4	35 - 38	1.96	64.78	1878.78	16.000	22.000	16.580
11	28355.11	967095.81	13.59	2.30	25.73	7	15 - 21	1.56	55.02	42.34	16.000	22.000	17.010
12	29404.38	945943.75	13.29	2.25	21.34	4	27 - 30	2.78	94.58	1091.61	16.000	22.000	16.580
13	28350.32	940328.19	13.21	2.24	30.21	7	21 - 27	2.57	90.76	37.55	16.000	22.000	16.580
14	28387.06	919585.13	12.92	2.19	17.82	4	36 - 39	1.83	64.48	74.29	16.000	22.000	16.610
15	26287.54	915527.81	12.86	2.18	19.82	4	24 - 27	1.89	71.91	-2025.23	16.000	22.000	16.500
16	25519.70	785862.75	11.04	1.87	20.17	4	32 - 35	1.75	68.62	-2793.08	16.000	22.000	16.610
17	28579.80	738361.81	10.37	1.76	31.51	6	26 - 31	2.31	80.98	267.03	16.000	22.000	16.710
18	24825.79	664397.38	9.33	1.58	18.49	4	31 - 34	1.79	72.09	-3486.98	16.000	22.000	16.870
19	27417.63	612741.63	8.61	1.46	20.70	4	24 - 27	1.37	49.90	-895.14	16.000	22.000	16.710
20	29849.29	533294.19	7.49	1.27	18.44	4	37 - 40	2.01	67.51	1536.52	16.000	22.000	16.520
21	30390.05	489616.28	6.88	1.17	17.17	4	33 - 36	3.91	128.68	2077.28	16.000	22.000	16.820
22	24563.17	464808.97	6.53	1.11	22.25	5	23 - 27	2.13	86.64	-3749.60	16.000	22.000	16.500
23	33858.40	446985.38	6.28	1.06	17.16	4	37 - 40	2.73	80.70	5545.63	16.000	22.000	16.580
24	28391.38	387231.00	5.44	0.92	18.11	4	22 - 25	2.10	74.03	78.61	16.000	22.000	16.580
25	24650.83	341141.88	4.79	0.81	22.62	4	33 - 36	1.79	72.62	-3661.94	16.000	22.000	16.610
26	27448.89	332453.13	4.67	0.79	23.02	4	24 - 27	1.93	70.37	-863.88	16.000	22.000	16.630
27	28407.78	320844.88	4.51	0.76	23.89	6	15 - 20	2.83	99.53	95.01	16.000	22.000	16.710
28	28337.99	290820.72	4.09	0.69	19.20	5	15 - 19	1.48	52.34	25.22	16.000	22.000	16.710
29	28448.85	273828.94	3.85	0.65	24.32	8	15 - 22	1.65	58.10	136.07	16.000	22.000	16.710
30	28578.36	273312.84	3.84	0.65	22.29	5	17 - 21	1.40	48.97	265.59	16.000	22.000	16.850
31	27312.32	259974.45	3.65	0.62	32.07	7	16 - 22	2.14	78.21	-1000.45	16.000	22.000	16.870
32	28389.45	226815.34	3.19	0.54	19.45	6	15 - 20	1.96	68.98	76.68	16.000	22.000	16.710
33	26372.59	169807.69	2.39	0.40	17.02	4	35 - 38	3.36	127.48	-1940.19	16.000	22.000	16.950
34	26930.63	167585.80	2.35	0.40	19.05	4	16 - 19	1.68	62.55	-1382.14	16.000	22.000	17.010
35	25820.96	124655.29	1.75	0.30	19.29	4	24 - 27	2.39	92.64	-2491.81	16.000	22.000	16.980
36	33001.83	99057.52	1.39	0.24	19.85	4	25 - 28	1.94	58.92	4689.06	16.000	22.000	16.820
37	28375.57	97741.04	1.37	0.23	16.56	4	15 - 18	1.58	55.62	62.80	16.000	22.000	16.710
38	23640.54	76876.50	1.08	0.18	16.79	4	19 - 22	2.55	107.84	-4672.23	16.000	22.000	16.610
39	30126.40	57881.77	0.81	0.14	9.67	4	17 - 20	1.28	42.52	1813.63	16.000	22.000	17.010
40	30164.99	57496.79	0.81	0.14	16.58	4	17 - 20	2.36	78.31	1852.22	16.000	22.000	16.980
41	28703.94	28839.38	0.41	0.07	13.89	4	34 - 37	1.76	61.28	391.16	16.000	22.000	16.870
42	25812.11	14556.73	0.20	0.03	6.33	4	14 - 17	2.00	77.39	-2500.67	16.000	22.000	16.710

Copyright© 2011 - 2017 Thermo Fisher Scientific
