# Supplementary material for: Reprogramming of bacterial virulence by lysine acetylation
Source: Nat Commun. 2026 Apr 27;17:3859. doi: 10.1038/s41467-026-72244-8 (PMC13125535; doi:10.1038/s41467-026-72244-8)
Supplement: Supplementary file 5 — Supplementary Data 3 [file 41467_2026_72244_MOESM5_ESM.zip › Supplementary_Data_3/3_SnCE1_74-310_H190A_4713_03_4173_SUMUP_RE_01152026_154801.pdf]

BioPharma Finder Report

Created: 15/01/2026 15:49:35

Sample Information	
Raw File Name	D:\Data\4713\4713_03.raw
Instrument Method	C:\Xcalibur\methods\UltiMate\NoFAIMS_Intact_Protein\Direct_Injection_MS1_IT_7K_RF60_35min.meth
Vial	RA3
Injection Volume (µL)	1
Sample Weight	0
Sample Volume (µL)	0
ISTD Amount	0
Dil Factor	1

Chromatogram Parameters	
Use Restricted Time	True
Time Limits	15.000 - 24.984 minutes
Scan Range	558 - 930
m/z Range	600 - 2000
Chromatogram Trace Type	TIC
Sensitivity	High
Rel. Intensity Threshold (%)	5

Chromatogram

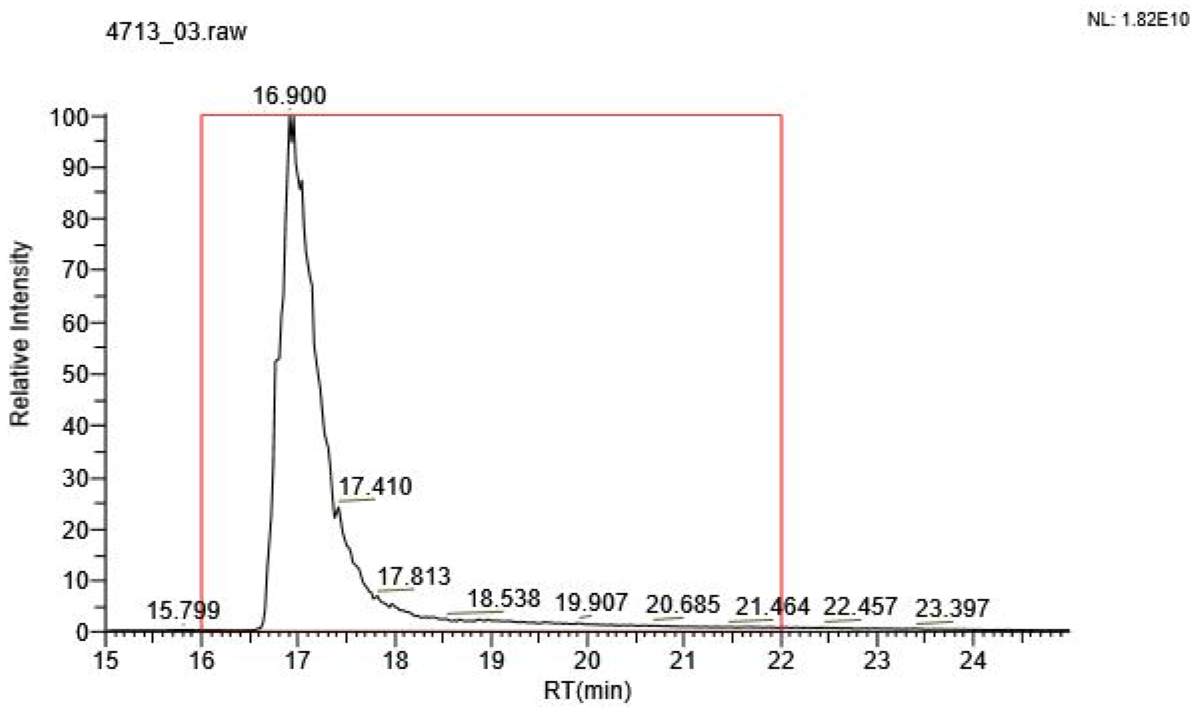

Copyright© 2011 - 2017 Thermo Fisher Scientific

Main Parameters (ReSpect™)	
Deconvolution Results Filter	
Output Mass Range	22500 - 35000
Deconvoluted Spectra Display Mode	Isotopic Profile (new)
Charge State Distribution	
Deconvolution Mass Tolerance	30 ppm
Choice of Peak Model	
Choice of Peak Model	Intact Protein
Resolution at 400 m/z	
Raw File Specific	2000
Generate XIC for Each Component	
Calculate XIC	True
**Advanced Parameters (ReSpect™)**	
Charge State Distribution	
Model Mass Range	8000 - 70000
Charge State Range	7 - 100
Minimum Adjacent Charges (low & high model mass)	4 - 4
Noise Parameters	
Rel. Abundance Threshold (%)	0
Deconvolution Quality	
Quality Score Threshold	0
Choice of Peak Model	
Target Mass	28000 Da
Peak Model Parameters	
Number of Peak Models	1
Left/Right Peak Shape	2:2
Peak Filter Parameters	
Peak Detection Minimum Significance Measure	1 Standard Deviations
Peak Detection Quality Measure	95%
Specialized Parameters	
Peak Model Width Factor	1
Intensity Threshold Scale	0.01
Deconvolution Parameters	
Noise Compensation	True
Charge Carrier	H
Negative Charge	False
**Source Spectra Parameters**	
Source Spectra Method	Average Over Selected Retention Time
RT Range	16.000 - 22.000 minutes

Copyright© 2011 - 2017 Thermo Fisher Scientific

Source Spectrum

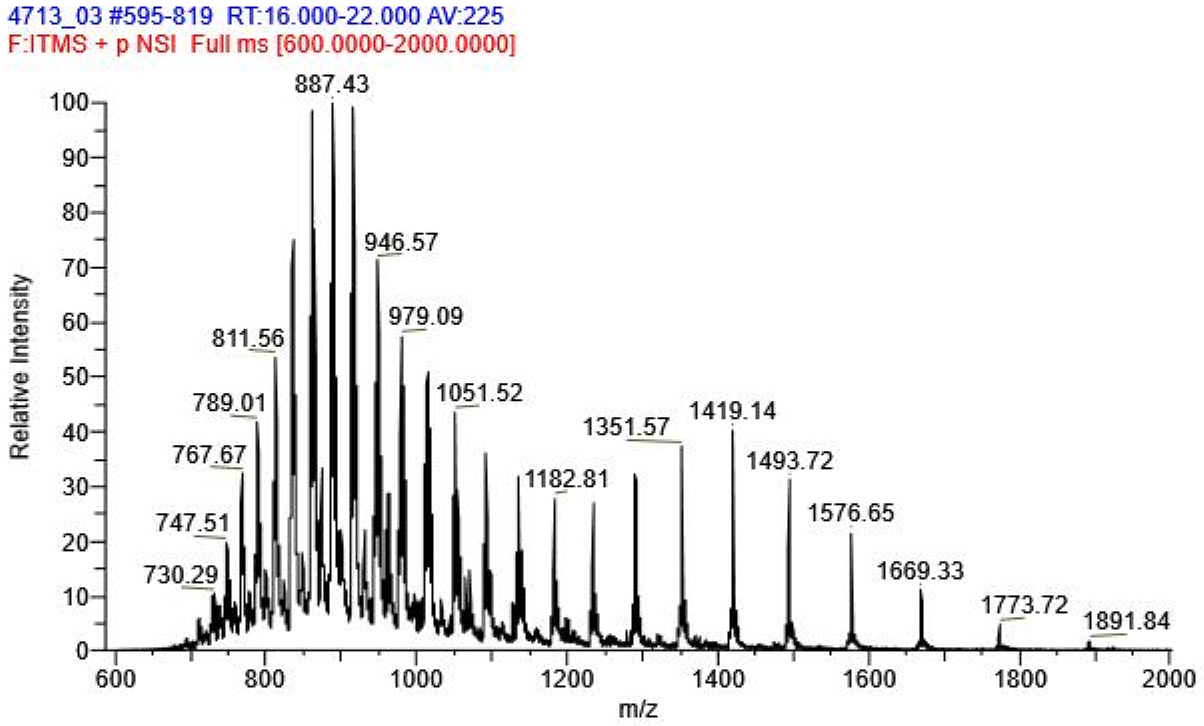

Copyright© 2011 - 2017 Thermo Fisher Scientific

Deconvoluted Spectrum

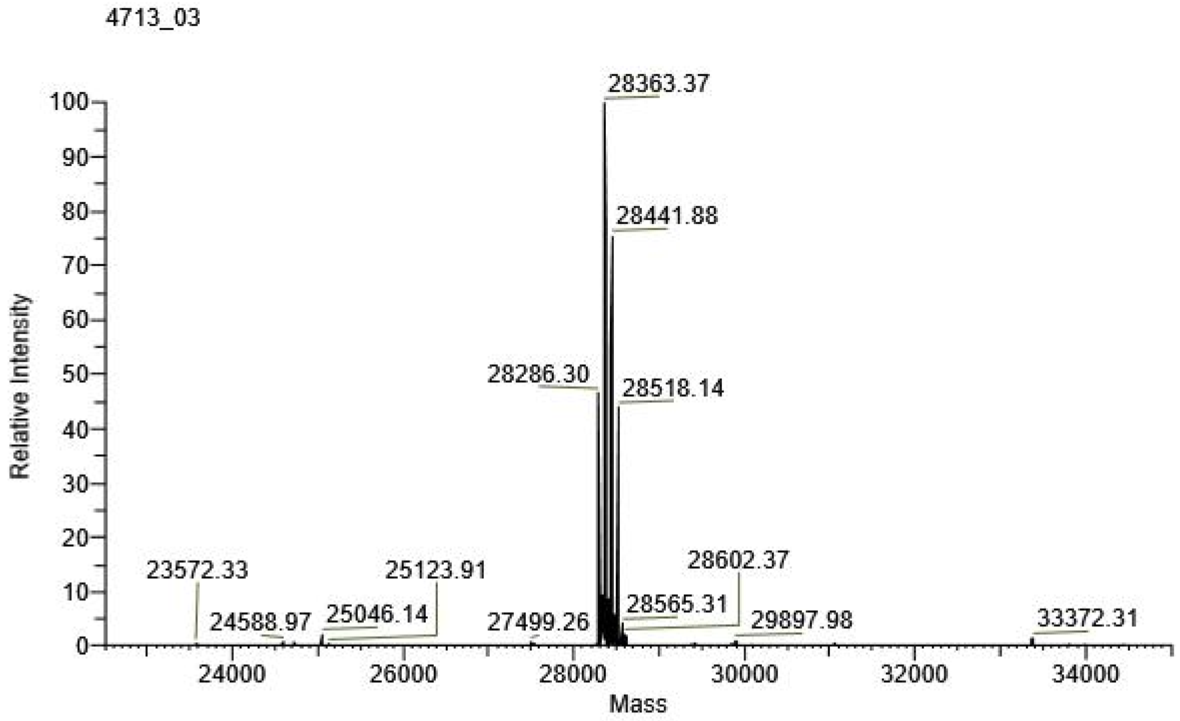

Copyright© 2011 - 2017 Thermo Fisher Scientific

ReSpect Masses Table													
Row Number	Average Mass	Intensity	Relative Abundance	Fractional Abundance	Score	Number of Charge States	Charge State Distribution	Mass Std Dev	PPM Std Dev	Delta Mass	Start Time (min)	Stop Time (min)	Apex RT
1	28363.37	214816432.00	100.00	31.10	111.87	23	15 - 37	1.73	60.88	0.00	16.000	22.000	16.900
2	28441.88	162379248.00	75.59	23.51	122.28	28	15 - 42	1.26	44.27	78.50	16.000	22.000	16.954
3	28286.30	100507448.00	46.79	14.55	84.55	22	15 - 36	1.54	54.30	-77.07	16.000	22.000	16.900
4	28518.14	94674376.00	44.07	13.71	101.43	24	16 - 39	1.56	54.71	154.77	16.000	22.000	16.954
5	28326.39	19128296.00	8.90	2.77	44.31	9	25 - 33	1.95	68.77	-36.99	16.000	22.000	16.900
6	28398.23	17168354.00	7.99	2.49	37.87	15	18 - 32	2.19	77.15	34.85	16.000	22.000	16.900
7	28341.61	13740350.00	6.40	1.99	40.43	10	15 - 24	1.26	44.47	-21.76	16.000	22.000	17.142
8	28480.92	11752127.00	5.47	1.70	29.79	6	26 - 31	1.97	69.10	117.54	16.000	22.000	16.900
9	28565.31	8811250.00	4.10	1.28	30.46	6	29 - 34	1.50	52.38	201.93	16.000	22.000	16.954
10	28315.71	5237733.00	2.44	0.76	35.61	9	16 - 24	1.32	46.53	-47.66	16.000	22.000	17.088
11	28602.37	4124353.25	1.92	0.60	20.88	4	28 - 31	2.91	101.64	238.99	16.000	22.000	16.900
12	28369.96	3851551.75	1.79	0.56	19.91	4	38 - 41	2.77	97.75	6.59	16.000	22.000	16.954
13	25046.14	3750593.25	1.75	0.54	22.12	4	24 - 27	0.81	32.28	-3317.23	16.000	22.000	16.927
14	28421.80	3714929.25	1.73	0.54	47.39	10	15 - 24	1.33	46.84	58.43	16.000	22.000	16.927
15	28471.62	3353138.25	1.56	0.49	24.84	5	21 - 25	2.69	94.35	108.24	16.000	22.000	16.927
16	33372.31	2719117.25	1.27	0.39	22.84	5	38 - 42	3.47	103.96	5008.94	16.000	22.000	16.900
17	28550.73	2364840.00	1.10	0.34	28.33	6	21 - 26	0.85	29.85	187.36	16.000	22.000	16.900
18	28401.29	1821787.13	0.85	0.26	17.14	4	34 - 37	1.70	59.96	37.91	16.000	22.000	16.900
19	29897.98	1698638.13	0.79	0.25	16.16	4	38 - 41	3.49	116.88	1534.61	16.000	22.000	16.900
20	28494.18	1644034.38	0.77	0.24	41.06	8	17 - 24	2.02	70.74	130.81	16.000	22.000	16.900
21	24588.97	1475572.38	0.69	0.21	16.96	4	28 - 31	1.76	71.72	-3774.40	16.000	22.000	17.034
22	24715.90	1351862.38	0.63	0.20	18.02	4	23 - 26	2.32	93.78	-3647.48	16.000	22.000	16.954
23	27499.26	1338321.13	0.62	0.19	18.02	4	25 - 28	2.01	73.26	-864.12	16.000	22.000	16.954
24	28462.61	1136391.13	0.53	0.16	26.97	6	15 - 20	3.15	110.73	99.24	16.000	22.000	17.115
25	31057.77	922596.38	0.43	0.13	19.39	4	28 - 31	2.02	65.04	2694.40	16.000	22.000	17.061
26	28320.43	880063.38	0.41	0.13	20.58	4	36 - 39	2.27	80.17	-42.94	16.000	22.000	16.900
27	29414.28	874310.63	0.41	0.13	15.13	4	26 - 29	1.25	42.37	1050.91	16.000	22.000	16.954
28	27535.52	690165.63	0.32	0.10	11.09	4	26 - 29	2.34	85.13	-827.86	16.000	22.000	16.954
29	23572.33	684676.75	0.32	0.10	20.89	4	23 - 26	0.89	37.91	-4791.04	16.000	22.000	16.927
30	25123.91	590838.25	0.28	0.09	20.72	4	16 - 19	1.72	68.34	-3239.46	16.000	22.000	17.034
31	28264.33	543074.56	0.25	0.08	13.33	10	15 - 24	1.55	54.73	-99.04	16.000	22.000	16.900
32	25048.03	538607.75	0.25	0.08	9.39	8	14 - 21	1.53	61.03	-3315.35	16.000	22.000	16.954
33	29850.42	501040.41	0.23	0.07	13.68	4	22 - 25	0.90	30.29	1487.05	16.000	22.000	16.900
34	28607.30	472205.41	0.22	0.07	10.21	6	18 - 23	1.03	36.06	243.93	16.000	22.000	16.900
35	33808.51	396719.56	0.18	0.06	6.88	4	24 - 27	3.57	105.67	5445.13	16.000	22.000	16.954
36	33987.34	342644.22	0.16	0.05	7.32	4	25 - 28	1.64	48.40	5623.97	16.000	22.000	16.927
37	34440.75	296839.88	0.14	0.04	8.00	4	31 - 34	3.00	86.97	6077.38	16.000	22.000	16.900
38	29375.69	221369.63	0.10	0.03	3.07	4	26 - 29	2.31	78.77	1012.32	16.000	22.000	16.900
39	30079.07	192001.66	0.09	0.03	7.00	4	18 - 21	2.88	95.65	1715.70	16.000	22.000	16.954

Copyright© 2011 - 2017 Thermo Fisher Scientific
