# Supplementary material for: Reprogramming of bacterial virulence by lysine acetylation
Source: Nat Commun. 2026 Apr 27;17:3859. doi: 10.1038/s41467-026-72244-8 (PMC13125535; doi:10.1038/s41467-026-72244-8)
Supplement: Supplementary file 5 — Supplementary Data 3 [file 41467_2026_72244_MOESM5_ESM.zip › Supplementary_Data_3/12_SnCE1_74-310_C256A_4713_12_4173_SUMUP_RE_01152026_154810.pdf]

BioPharma Finder Report

Created: 15/01/2026 15:54:55

Sample Information	
Raw File Name	D:\Data\4713\4713_12.raw
Instrument Method	C:\Xcalibur\methods\UltiMate\NoFAIMS_Intact_Protein\Direct_Injection_MS1_IT_7K_RF60_35min.meth
Vial	RA12
Injection Volume (µL)	1
Sample Weight	0
Sample Volume (µL)	0
ISTD Amount	0
Dil Factor	1

Chromatogram Parameters	
Use Restricted Time	True
Time Limits	15.000 - 24.984 minutes
Scan Range	558 - 930
m/z Range	600 - 2000
Chromatogram Trace Type	TIC
Sensitivity	High
Rel. Intensity Threshold (%)	5

Chromatogram

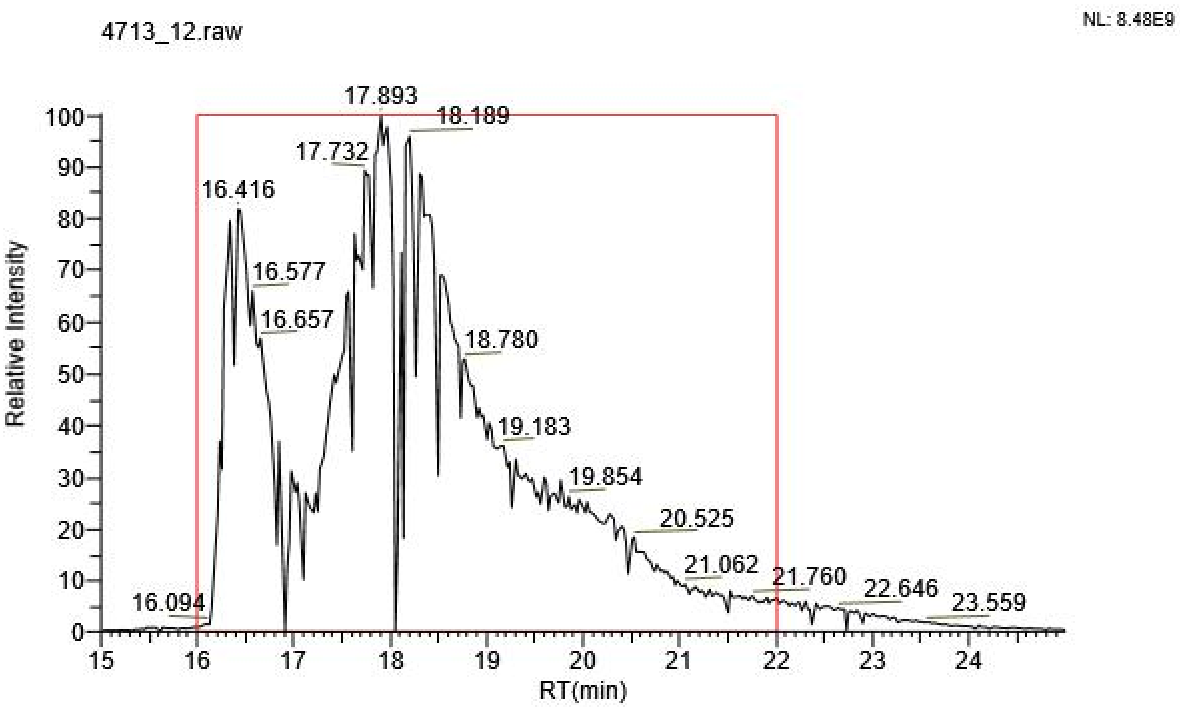

Copyright© 2011 - 2017 Thermo Fisher Scientific

01/15/2026 03:54:57

Main Parameters (ReSpect™)	
Deconvolution Results Filter	
Output Mass Range	22500 - 35000
Deconvoluted Spectra Display Mode	Isotopic Profile (new)
Charge State Distribution	
Deconvolution Mass Tolerance	30 ppm
Choice of Peak Model	
Choice of Peak Model	Intact Protein
Resolution at 400 m/z	
Raw File Specific	2000
Generate XIC for Each Component	
Calculate XIC	True

Advanced Parameters (ReSpect™)	
Charge State Distribution	
Model Mass Range	8000 - 70000
Charge State Range	7 - 100
Minimum Adjacent Charges (low & high model mass)	4 - 4
Noise Parameters	
Rel. Abundance Threshold (%)	0
Deconvolution Quality	
Quality Score Threshold	0
Choice of Peak Model	
Target Mass	28000 Da
Peak Model Parameters	
Number of Peak Models	1
Left/Right Peak Shape	2:2
Peak Filter Parameters	
Peak Detection Minimum Significance Measure	1 Standard Deviations
Peak Detection Quality Measure	95%
Specialized Parameters	
Peak Model Width Factor	1
Intensity Threshold Scale	0.01
Deconvolution Parameters	
Noise Compensation	True
Charge Carrier	H
Negative Charge	False

Source Spectra Parameters	
Source Spectra Method	Average Over Selected Retention Time
RT Range	16.000 - 22.000 minutes

Copyright© 2011 - 2017 Thermo Fisher Scientific

Source Spectrum

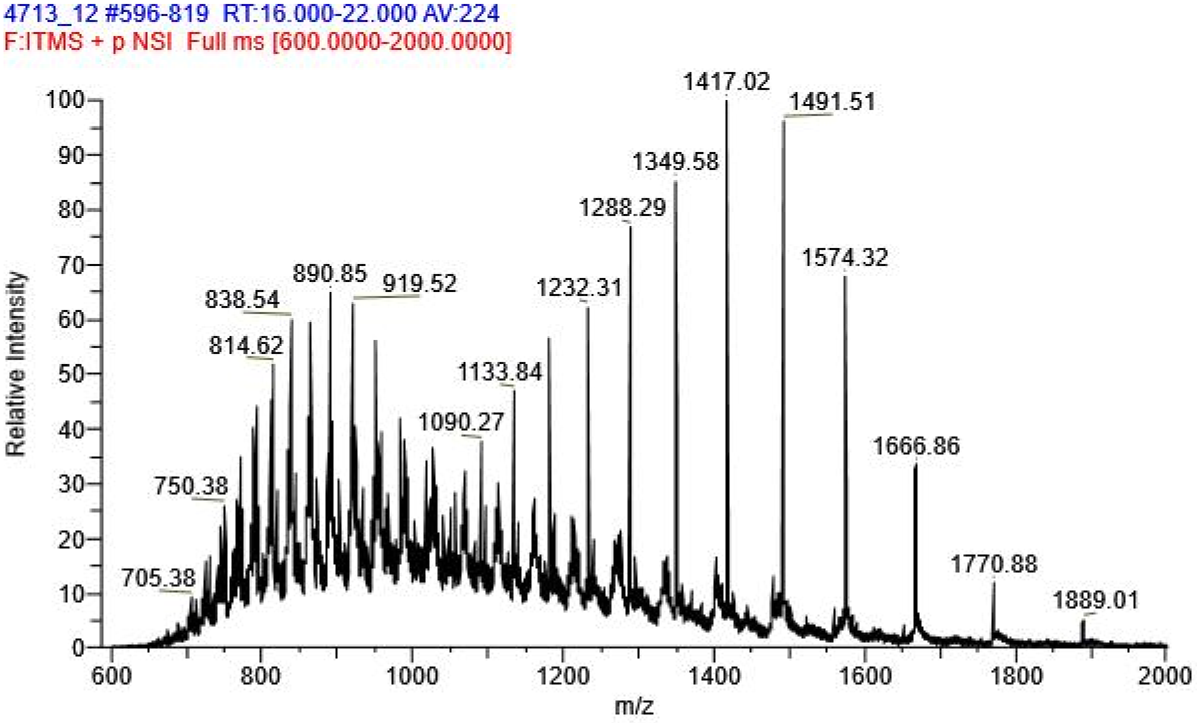

Copyright© 2011 - 2017 Thermo Fisher Scientific

Deconvoluted Spectrum

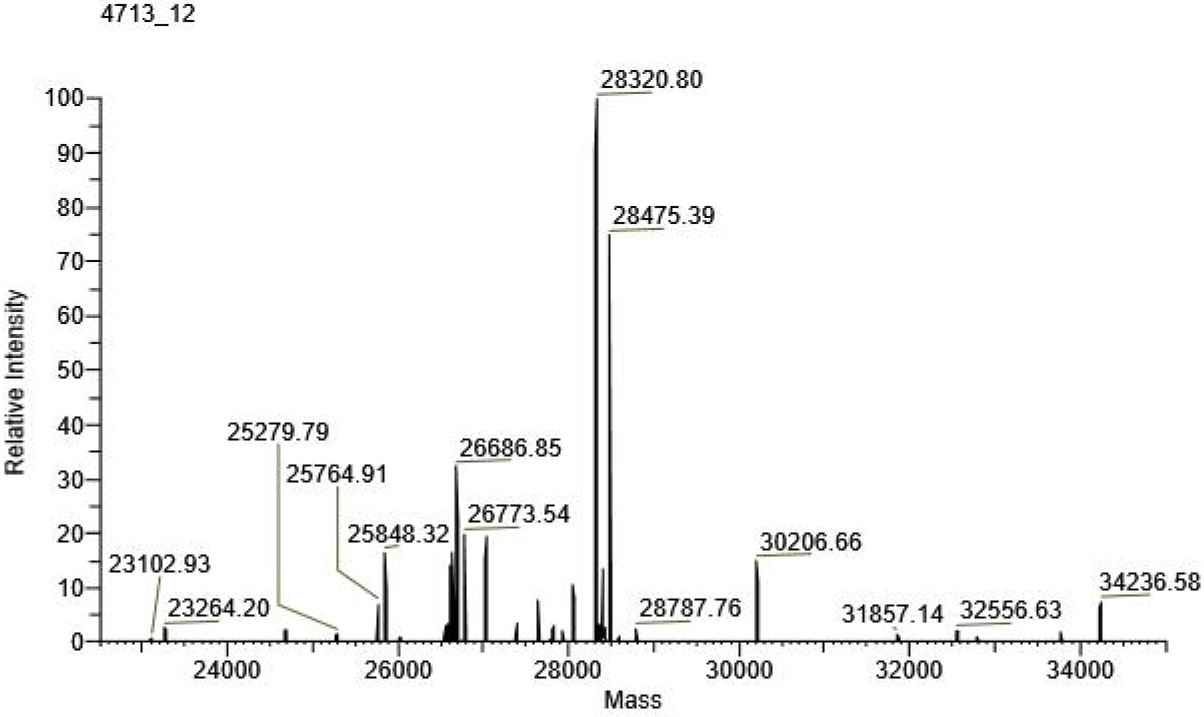

Copyright© 2011 - 2017 Thermo Fisher Scientific

ReSpect Masses Table

Row Number	Average Mass	Intensity	Relative Abundance	Fractional Abundance	Score	Number of Charge States	Charge State Distribution	Mass Std Dev	PPM Std Dev	Delta Mass	Start Time (min)	Stop Time (min)	Apex RT
1	28320.80	119706792.00	100.00	25.27	61.43	13	15 - 27	0.98	34.65	0.00	16.000	22.000	16.500
2	28475.39	55916056.00	46.71	11.81	79.94	18	15 - 32	0.77	26.92	154.59	16.000	22.000	16.340
3	26686.85	37166216.00	31.05	7.85	53.21	10	20 - 29	1.51	56.51	-1633.95	16.000	22.000	18.320
4	28476.17	34209952.00	28.58	7.22	27.14	5	34 - 38	1.32	46.23	155.37	16.000	22.000	16.310
5	26773.54	23597998.00	19.71	4.98	31.78	7	33 - 39	2.44	91.21	-1547.25	16.000	22.000	17.970
6	27028.51	23172264.00	19.36	4.89	63.96	12	22 - 33	2.55	94.27	-1292.29	16.000	22.000	16.420
7	25848.32	19509000.00	16.30	4.12	21.21	4	28 - 31	1.90	73.52	-2472.47	16.000	22.000	17.950
8	30206.66	17869774.00	14.93	3.77	22.68	4	33 - 36	2.35	77.91	1885.86	16.000	22.000	16.310
9	26610.44	16729894.00	13.98	3.53	59.76	13	15 - 27	2.23	83.64	-1710.36	16.000	22.000	18.190
10	26637.12	14518001.00	12.13	3.07	29.00	5	32 - 36	1.75	65.85	-1683.68	16.000	22.000	17.920
11	28399.94	13208224.00	11.03	2.79	22.86	4	29 - 32	2.76	97.19	79.14	16.000	22.000	17.890
12	28052.46	12506046.00	10.45	2.64	35.21	10	15 - 24	1.34	47.62	-268.34	16.000	22.000	16.440
13	27638.40	9085420.00	7.59	1.92	24.59	5	26 - 30	1.35	48.72	-682.39	16.000	22.000	18.320
14	34236.58	8650276.00	7.23	1.83	20.50	4	41 - 44	0.69	20.05	5915.78	16.000	22.000	16.340
15	25764.91	8125676.00	6.79	1.72	18.44	4	27 - 30	2.15	83.59	-2555.89	16.000	22.000	17.890
16	26634.63	5828792.00	4.87	1.23	38.12	8	20 - 27	2.20	82.60	-1686.17	16.000	22.000	17.890
17	27391.58	4028535.50	3.37	0.85	29.77	7	16 - 22	1.33	48.73	-929.22	16.000	22.000	16.680
18	26579.04	3901241.50	3.26	0.82	27.42	6	18 - 23	1.38	51.85	-1741.76	16.000	22.000	18.320
19	28362.21	3740345.00	3.12	0.79	31.17	6	20 - 25	2.40	84.55	41.42	16.000	22.000	18.190
20	26553.29	3466846.00	2.90	0.73	33.99	7	19 - 25	1.92	72.50	-1767.51	16.000	22.000	18.300
21	28420.78	3034466.25	2.53	0.64	26.86	5	18 - 22	1.59	56.01	99.98	16.000	22.000	16.470
22	23264.20	3012282.25	2.52	0.64	18.79	4	24 - 27	2.10	90.35	-5056.60	16.000	22.000	17.890
23	28398.91	2823626.75	2.36	0.60	36.81	7	21 - 27	1.27	44.84	78.12	16.000	22.000	18.320
24	27819.60	2790228.50	2.33	0.59	17.14	4	22 - 25	2.16	77.71	-501.20	16.000	22.000	18.190
25	28787.76	2763069.00	2.31	0.58	20.14	4	25 - 28	2.51	87.33	466.96	16.000	22.000	18.160
26	24678.17	2519667.50	2.10	0.53	17.28	4	24 - 27	2.24	90.57	-3642.63	16.000	22.000	16.340
27	27923.68	2345827.75	1.96	0.50	20.03	4	23 - 26	1.83	65.39	-397.12	16.000	22.000	17.970
28	32556.63	2312796.25	1.93	0.49	26.02	5	26 - 30	2.46	75.47	4235.84	16.000	22.000	18.300
29	26682.71	2284457.50	1.91	0.48	17.88	4	16 - 19	1.60	59.84	-1638.08	16.000	22.000	18.350
30	28349.21	2217638.00	1.85	0.47	15.02	4	15 - 18	2.31	81.45	28.41	16.000	22.000	16.470
31	33765.62	2100338.50	1.75	0.44	19.82	4	24 - 27	2.60	76.99	5444.82	16.000	22.000	18.220
32	26657.73	2000935.25	1.67	0.42	20.94	4	20 - 23	1.57	58.99	-1663.07	16.000	22.000	18.320
33	25279.79	1627821.38	1.36	0.34	19.43	4	16 - 19	2.53	99.95	-3041.01	16.000	22.000	18.380
34	31857.14	1557166.13	1.30	0.33	17.48	4	28 - 31	3.18	99.82	3536.34	16.000	22.000	18.300
35	28590.58	1109997.38	0.93	0.23	20.23	4	19 - 22	2.42	84.75	269.78	16.000	22.000	18.590
36	32784.91	1037942.88	0.87	0.22	17.91	4	23 - 26	3.33	101.60	4464.11	16.000	22.000	18.590
37	26021.90	836384.75	0.70	0.18	16.49	4	17 - 20	2.22	85.15	-2298.90	16.000	22.000	18.430
38	27818.45	672962.00	0.56	0.14	21.09	4	15 - 18	2.39	85.83	-502.35	16.000	22.000	18.350
39	27796.38	670100.31	0.56	0.14	19.20	4	24 - 27	2.07	74.56	-524.42	16.000	22.000	18.190
40	23102.93	667915.56	0.56	0.14	16.60	4	13 - 16	2.50	108.04	-5217.87	16.000	22.000	16.440
41	27367.31	322604.44	0.27	0.07	19.40	4	18 - 21	0.69	25.33	-953.49	16.000	22.000	18.350

Copyright© 2011 - 2017 Thermo Fisher Scientific
