# Supplementary material for: Reprogramming of bacterial virulence by lysine acetylation
Source: Nat Commun. 2026 Apr 27;17:3859. doi: 10.1038/s41467-026-72244-8 (PMC13125535; doi:10.1038/s41467-026-72244-8)
Supplement: Supplementary file 5 — Supplementary Data 3 [file 41467_2026_72244_MOESM5_ESM.zip › Supplementary_Data_3/15_SnCE1_74-310_S208A_C256A_4713_15_4173_SUMUP_RE_01152026_154906.pdf]

BioPharma Finder Report

Created: 15/01/2026 15:56:36

Sample Information	
Raw File Name	D:\Data\4713\4713_15.raw
Instrument Method	C:\Xcalibur\methods\UltiMate\NoFAIMS_Intact_Protein\Direct_Injection_MS1_IT_7K_RF60_35min.meth
Vial	RB3
Injection Volume (µL)	1
Sample Weight	0
Sample Volume (µL)	0
ISTD Amount	0
Dil Factor	1

Chromatogram Parameters	
Use Restricted Time	True
Time Limits	15.000 - 24.984 minutes
Scan Range	558 - 930
m/z Range	600 - 2000
Chromatogram Trace Type	TIC
Sensitivity	High
Rel. Intensity Threshold (%)	5

Chromatogram

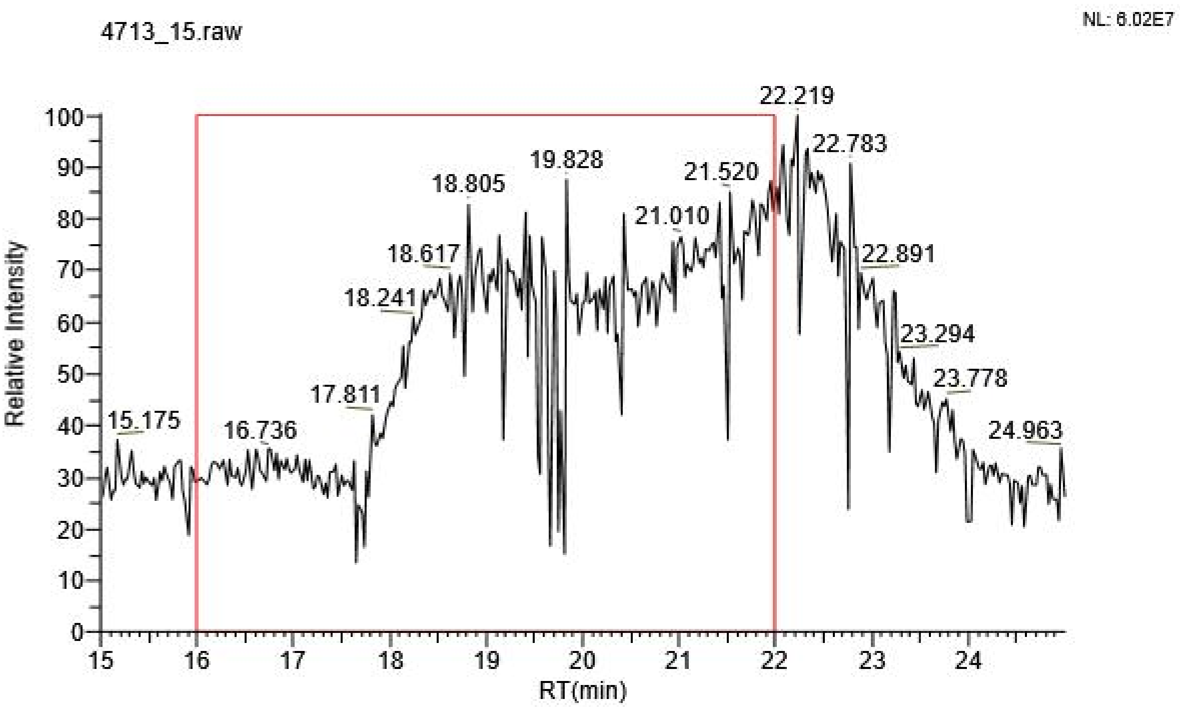

Copyright© 2011 - 2017 Thermo Fisher Scientific

01/15/2026 03:56:38

Main Parameters (ReSpect™)	
Deconvolution Results Filter	
Output Mass Range	22500 - 35000
Deconvoluted Spectra Display Mode	Isotopic Profile (new)
Charge State Distribution	
Deconvolution Mass Tolerance	30 ppm
Choice of Peak Model	
Choice of Peak Model	Intact Protein
Resolution at 400 m/z	
Raw File Specific	2000
Generate XIC for Each Component	
Calculate XIC	True

Advanced Parameters (ReSpect™)	
Charge State Distribution	
Model Mass Range	8000 - 70000
Charge State Range	7 - 100
Minimum Adjacent Charges (low & high model mass)	4 - 4
Noise Parameters	
Rel. Abundance Threshold (%)	0
Deconvolution Quality	
Quality Score Threshold	0
Choice of Peak Model	
Target Mass	28000 Da
Peak Model Parameters	
Number of Peak Models	1
Left/Right Peak Shape	2:2
Peak Filter Parameters	
Peak Detection Minimum Significance Measure	1 Standard Deviations
Peak Detection Quality Measure	95%
Specialized Parameters	
Peak Model Width Factor	1
Intensity Threshold Scale	0.01
Deconvolution Parameters	
Noise Compensation	True
Charge Carrier	H
Negative Charge	False

Source Spectra Parameters	
Source Spectra Method	Average Over Selected Retention Time
RT Range	16.000 - 22.000 minutes

Copyright© 2011 - 2017 Thermo Fisher Scientific

Source Spectrum

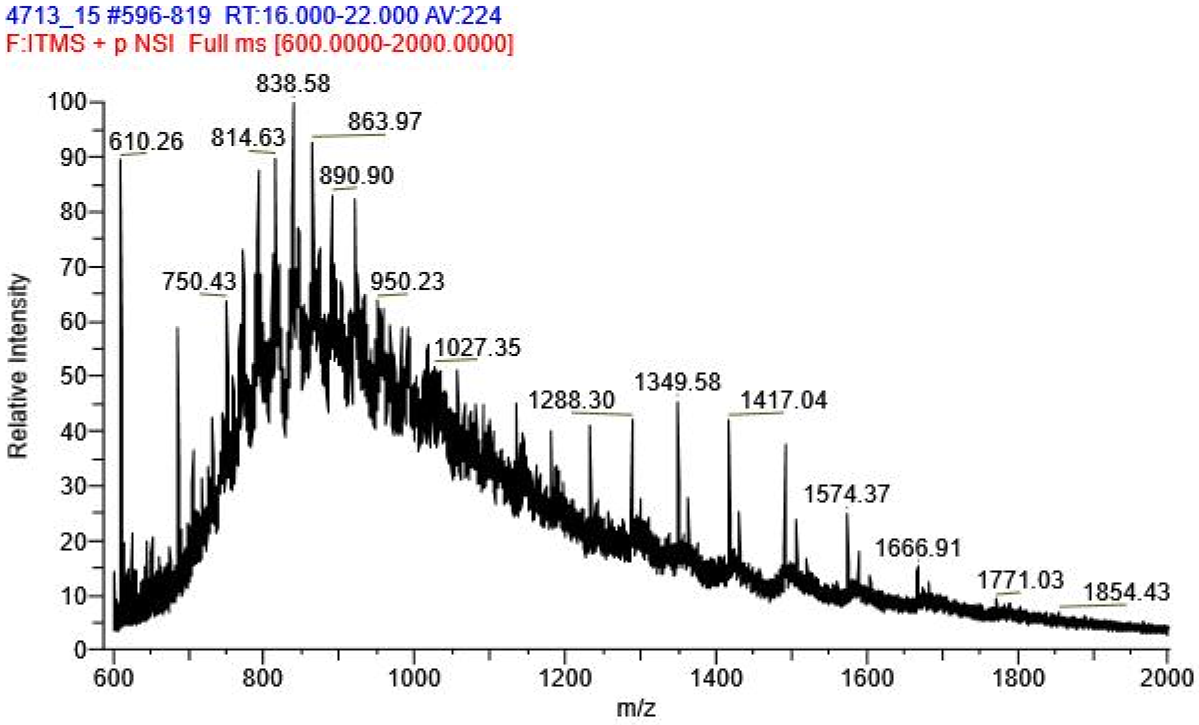

Copyright© 2011 - 2017 Thermo Fisher Scientific

01/15/2026 03:56:38

Deconvoluted Spectrum

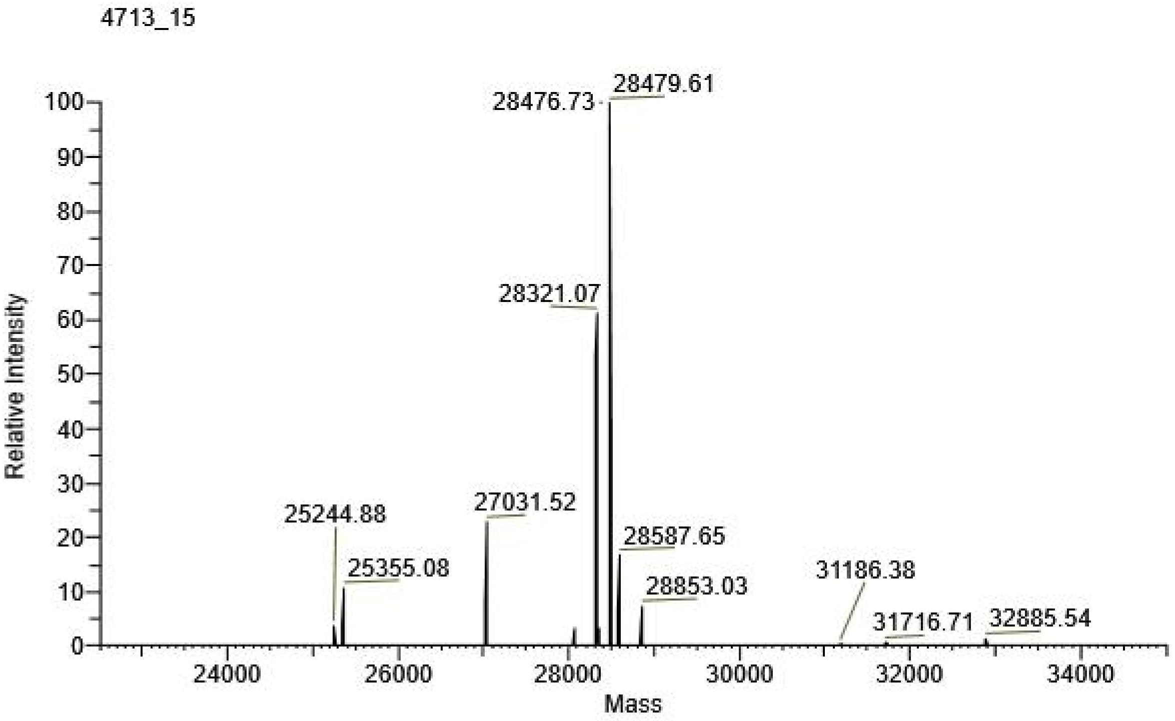

Copyright© 2011 - 2017 Thermo Fisher Scientific
01/15/2026 03:56:38

ReSpect Masses Table													
Row Number	Average Mass	Intensity	Relative Abundance	Fractional Abundance	Score	Number of Charge States	Charge State Distribution	Mass Std Dev	PPM Std Dev	Delta Mass	Start Time (min)	Stop Time (min)	Apex RT
1	28479.61	271460.69	100.00	39.75	68.13	13	29 - 41	1.61	56.57	0.00	16.000	22.000	18.540
2	28321.07	179952.70	66.29	26.35	55.07	12	15 - 26	1.38	48.83	-158.54	16.000	22.000	18.810
3	27031.52	67074.55	24.71	9.82	20.36	4	33 - 36	2.10	77.72	-1448.09	16.000	22.000	18.290
4	28587.65	49207.95	18.13	7.21	44.33	9	15 - 23	1.08	37.81	108.04	16.000	22.000	18.940
5	25355.08	31053.95	11.44	4.55	19.62	4	36 - 39	2.46	96.99	-3124.53	16.000	22.000	19.400
6	28476.73	27776.47	10.23	4.07	20.74	4	24 - 27	1.10	38.58	-2.88	16.000	22.000	21.950
7	28853.03	21113.53	7.78	3.09	29.91	6	16 - 21	1.74	60.39	373.42	16.000	22.000	19.400
8	25244.88	10507.22	3.87	1.54	21.83	4	16 - 19	2.25	89.32	-3234.73	16.000	22.000	21.950
9	28063.90	9394.23	3.46	1.38	20.12	4	39 - 42	2.43	86.69	-415.71	16.000	22.000	19.400
10	28346.20	9210.73	3.39	1.35	20.95	4	17 - 20	2.07	73.15	-133.42	16.000	22.000	18.810
11	32885.54	3529.92	1.30	0.52	16.97	4	50 - 53	2.99	91.03	4405.92	16.000	22.000	19.400
12	31716.71	1523.27	0.56	0.22	19.31	4	47 - 50	3.06	96.58	3237.10	16.000	22.000	19.400
13	31186.38	1029.54	0.38	0.15	15.30	4	21 - 24	2.21	70.88	2706.77	16.000	22.000	19.070

Copyright© 2011 - 2017 Thermo Fisher Scientific
