# Supplementary material for: Reprogramming of bacterial virulence by lysine acetylation
Source: Nat Commun. 2026 Apr 27;17:3859. doi: 10.1038/s41467-026-72244-8 (PMC13125535; doi:10.1038/s41467-026-72244-8)
Supplement: Supplementary file 5 — Supplementary Data 3 [file 41467_2026_72244_MOESM5_ESM.zip › Supplementary_Data_3/10_SnCE1_74-310_K231R_E217D_4713_10_4173_SUMUP_RE_01152026_154808.pdf]

BioPharma Finder Report

Created: 15/01/2026 15:53:44

Sample Information	
Raw File Name	D:\Data\4713\4713_10.raw
Instrument Method	C:\Xcalibur\methods\UltiMate\NoFAIMS_Intact_Protein\Direct_Injection_MS1_IT_7K_RF60_35min.meth
Vial	RA10
Injection Volume (µL)	1
Sample Weight	0
Sample Volume (µL)	0
ISTD Amount	0
Dil Factor	1

Chromatogram Parameters	
Use Restricted Time	True
Time Limits	15.000 - 24.984 minutes
Scan Range	558 - 930
m/z Range	600 - 2000
Chromatogram Trace Type	TIC
Sensitivity	High
Rel. Intensity Threshold (%)	5

Chromatogram

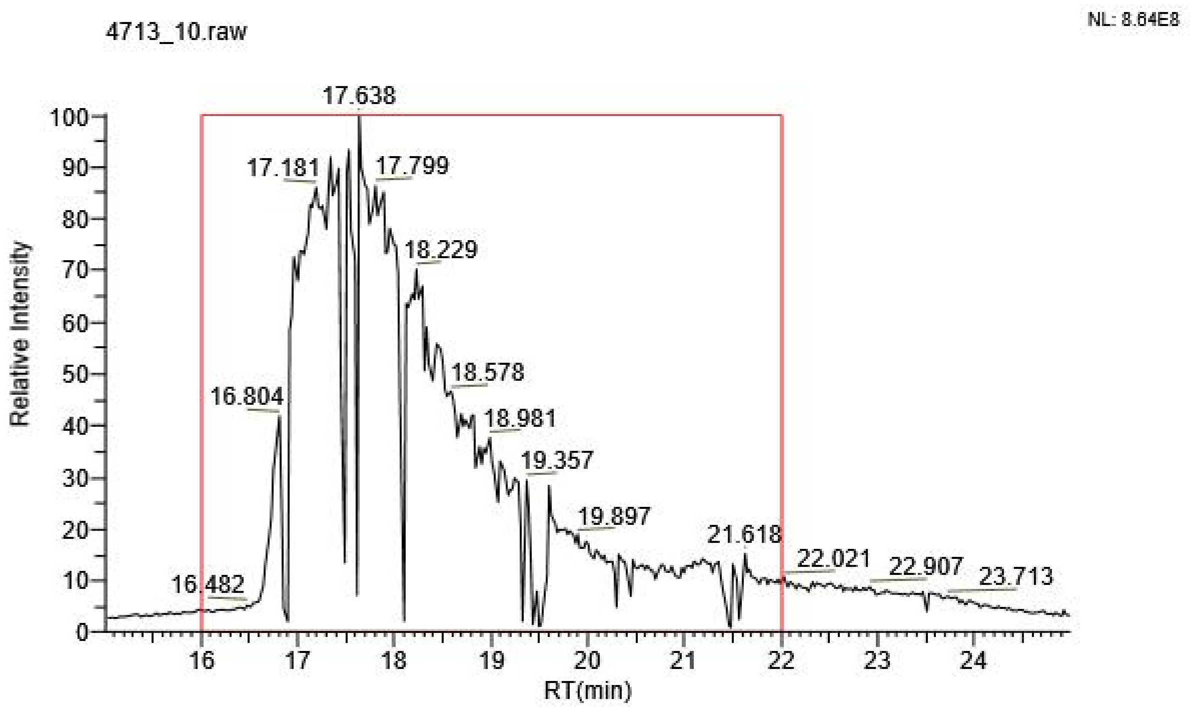

Copyright© 2011 - 2017 Thermo Fisher Scientific

Main Parameters (ReSpect™)	
Deconvolution Results Filter	
Output Mass Range	22500 - 35000
Deconvoluted Spectra Display Mode	Isotopic Profile (new)
Charge State Distribution	
Deconvolution Mass Tolerance	30 ppm
Choice of Peak Model	
Choice of Peak Model	Intact Protein
Resolution at 400 m/z	
Raw File Specific	2000
Generate XIC for Each Component	
Calculate XIC	True
**Advanced Parameters (ReSpect™)**	
Charge State Distribution	
Model Mass Range	8000 - 70000
Charge State Range	7 - 100
Minimum Adjacent Charges (low & high model mass)	4 - 4
Noise Parameters	
Rel. Abundance Threshold (%)	0
Deconvolution Quality	
Quality Score Threshold	0
Choice of Peak Model	
Target Mass	28000 Da
Peak Model Parameters	
Number of Peak Models	1
Left/Right Peak Shape	2:2
Peak Filter Parameters	
Peak Detection Minimum Significance Measure	1 Standard Deviations
Peak Detection Quality Measure	95%
Specialized Parameters	
Peak Model Width Factor	1
Intensity Threshold Scale	0.01
Deconvolution Parameters	
Noise Compensation	True
Charge Carrier	H
Negative Charge	False
**Source Spectra Parameters**	
Source Spectra Method	Average Over Selected Retention Time
RT Range	16.000 - 22.000 minutes

Copyright© 2011 - 2017 Thermo Fisher Scientific

Source Spectrum

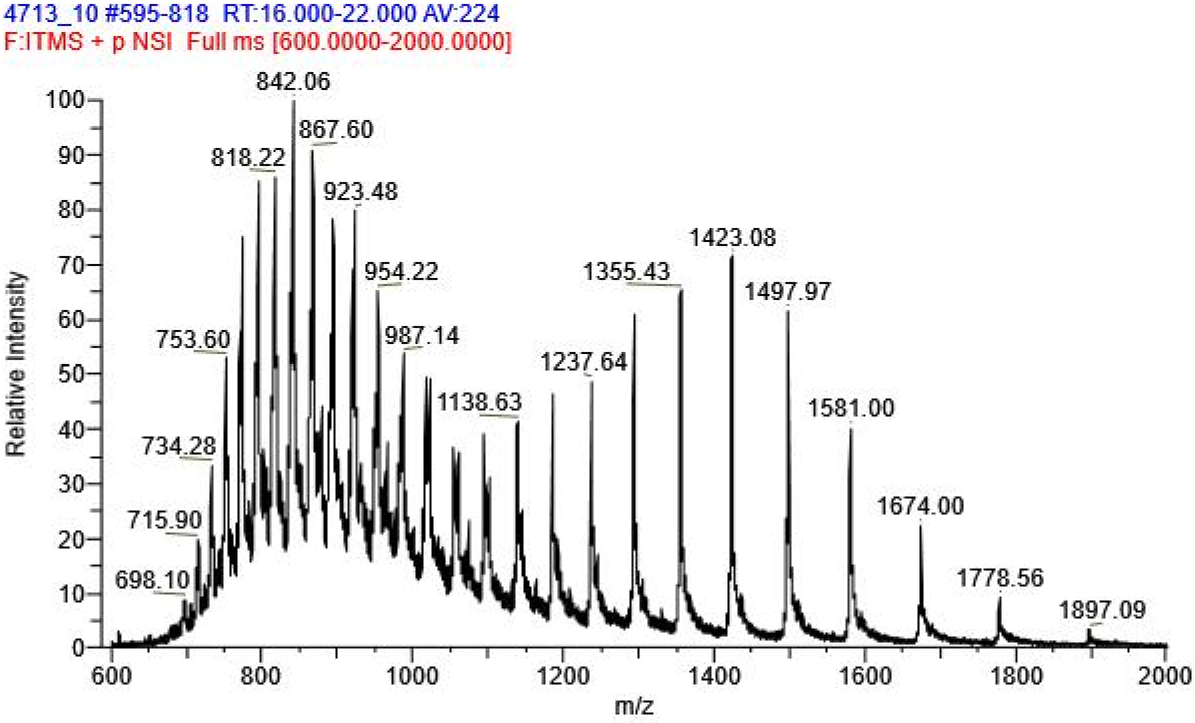

Copyright© 2011 - 2017 Thermo Fisher Scientific

Deconvoluted Spectrum

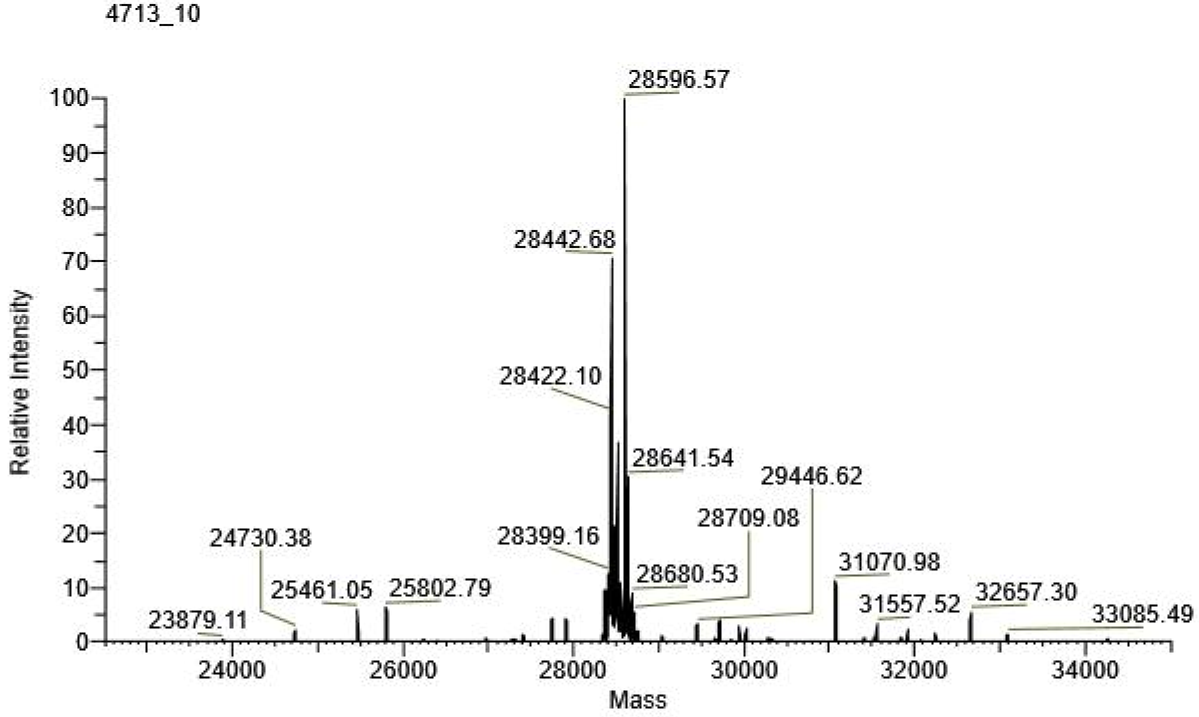

Copyright© 2011 - 2017 Thermo Fisher Scientific

01/15/2026 03:53:46

ReSpect Masses Table													
Row Number	Average Mass	Intensity	Relative Abundance	Fractional Abundance	Score	Number of Charge States	Charge State Distribution	Mass Std Dev	PPM Std Dev	Delta Mass	Start Time (min)	Stop Time (min)	Apex RT
1	28596.57	13063528.00	100.00	21.57	110.37	25	16 - 40	1.66	58.19	0.00	16.000	22.000	17.180
2	28442.68	7005843.50	53.63	11.57	63.22	13	15 - 27	0.56	19.51	-153.89	16.000	22.000	17.640
3	28641.54	3781645.25	28.95	6.24	58.09	11	28 - 38	3.70	129.27	44.97	16.000	22.000	17.640
4	28422.10	3776759.25	28.91	6.24	54.00	12	15 - 26	0.95	33.51	-174.47	16.000	22.000	17.640
5	28514.08	3486531.25	26.69	5.76	27.44	5	34 - 38	1.90	66.63	-82.50	16.000	22.000	17.100
6	28465.94	2781569.00	21.29	4.59	55.93	13	15 - 27	2.57	90.14	-130.63	16.000	22.000	17.640
7	28520.98	2425897.00	18.57	4.01	75.88	15	16 - 30	1.74	60.96	-75.60	16.000	22.000	17.640
8	28443.68	2264779.00	17.34	3.74	28.86	5	30 - 34	1.87	65.91	-152.90	16.000	22.000	17.340
9	28399.16	1583965.25	12.13	2.62	52.52	11	16 - 26	2.19	77.17	-197.41	16.000	22.000	17.500
10	31070.98	1463164.88	11.20	2.42	21.03	4	34 - 37	2.65	85.23	2474.41	16.000	22.000	17.640
11	28485.44	1303105.38	9.98	2.15	25.65	7	15 - 21	2.26	79.51	-111.13	16.000	22.000	17.640
12	28548.14	1289970.50	9.87	2.13	39.88	8	21 - 28	2.36	82.66	-48.43	16.000	22.000	17.640
13	28680.53	1085945.50	8.31	1.79	22.53	4	33 - 36	3.05	106.36	83.96	16.000	22.000	17.340
14	25802.79	819909.50	6.28	1.35	22.59	5	25 - 29	2.07	80.26	-2793.79	16.000	22.000	17.640
15	28365.28	783627.69	6.00	1.29	27.10	6	30 - 35	2.34	82.57	-231.29	16.000	22.000	16.970
16	25461.05	771358.19	5.90	1.27	20.78	4	32 - 35	1.18	46.35	-3135.53	16.000	22.000	17.420
17	28575.03	745239.56	5.70	1.23	43.17	9	16 - 24	2.08	72.82	-21.54	16.000	22.000	17.130
18	28709.08	704366.88	5.39	1.16	49.68	11	15 - 25	1.75	60.95	112.51	16.000	22.000	17.340
19	32657.30	700926.75	5.37	1.16	21.76	4	31 - 34	1.08	33.16	4060.72	16.000	22.000	17.640
20	28495.75	627775.44	4.81	1.04	22.20	5	22 - 26	3.16	110.90	-100.83	16.000	22.000	17.800
21	27745.98	552153.44	4.23	0.91	29.33	6	26 - 31	2.84	102.25	-850.59	16.000	22.000	17.640
22	27910.13	546572.69	4.18	0.90	17.52	4	30 - 33	3.02	108.33	-686.45	16.000	22.000	17.640
23	28626.29	517860.03	3.96	0.86	26.80	5	21 - 25	1.50	52.40	29.71	16.000	22.000	17.530
24	29710.06	516680.25	3.96	0.85	17.93	4	23 - 26	2.78	93.60	1113.48	16.000	22.000	17.640
25	28539.86	462968.59	3.54	0.76	20.33	5	16 - 20	1.88	65.89	-56.71	16.000	22.000	17.640
26	31557.52	427820.25	3.27	0.71	20.33	4	34 - 37	2.32	73.55	2960.95	16.000	22.000	17.640
27	29446.62	420328.28	3.22	0.69	19.65	4	28 - 31	1.75	59.31	850.05	16.000	22.000	17.640
28	28371.46	376787.63	2.88	0.62	23.08	5	19 - 23	1.63	57.56	-225.11	16.000	22.000	17.640
29	28518.79	376713.03	2.88	0.62	18.78	4	39 - 42	2.66	93.19	-77.79	16.000	22.000	17.420
30	29938.12	369124.31	2.83	0.61	19.96	4	19 - 22	2.33	77.85	1341.55	16.000	22.000	17.660
31	28503.88	365913.59	2.80	0.60	16.96	4	16 - 19	2.26	79.27	-92.70	16.000	22.000	17.640
32	28407.35	346601.97	2.65	0.57	19.94	4	32 - 35	2.28	80.32	-189.22	16.000	22.000	17.150
33	28688.02	315153.63	2.41	0.52	27.03	7	15 - 21	2.51	87.58	91.45	16.000	22.000	17.640
34	30023.78	314709.31	2.41	0.52	18.62	4	19 - 22	1.67	55.64	1427.20	16.000	22.000	17.640
35	31919.73	291644.38	2.23	0.48	19.83	4	28 - 31	2.65	83.08	3323.15	16.000	22.000	17.660
36	28366.83	286571.78	2.19	0.47	25.79	6	22 - 27	2.57	90.52	-229.74	16.000	22.000	17.130
37	24730.38	268454.16	2.05	0.44	17.03	4	29 - 32	2.52	102.10	-3866.19	16.000	22.000	17.640
38	28759.48	246406.39	1.89	0.41	26.00	5	19 - 23	2.46	85.51	162.90	16.000	22.000	17.530
39	28346.51	242196.23	1.85	0.40	34.44	7	17 - 23	2.37	83.50	-250.07	16.000	22.000	17.340
40	28637.70	233506.47	1.79	0.39	29.22	6	17 - 22	2.27	79.20	41.12	16.000	22.000	17.800
41	28734.34	221152.47	1.69	0.37	20.32	4	19 - 22	2.71	94.39	137.77	16.000	22.000	17.880
42	28557.70	216310.97	1.66	0.36	18.96	4	16 - 19	0.39	13.63	-38.87	16.000	22.000	17.660
43	28617.22	215194.38	1.65	0.36	18.63	4	17 - 20	2.48	86.73	20.65	16.000	22.000	17.660
44	32238.84	200197.89	1.53	0.33	15.57	4	25 - 28	2.05	63.59	3642.27	16.000	22.000	17.880
45	33085.49	162108.47	1.24	0.27	17.20	4	29 - 32	1.89	57.25	4488.91	16.000	22.000	17.660
46	27406.48	162011.00	1.24	0.27	18.73	4	21 - 24	3.04	110.79	-1190.10	16.000	22.000	17.210
47	29034.15	135462.67	1.04	0.22	19.34	4	27 - 30	1.98	68.05	437.57	16.000	22.000	17.530
48	31836.28	91531.84	0.70	0.15	17.75	4	19 - 22	3.26	102.31	3239.71	16.000	22.000	17.400

Copyright© 2011 - 2017 Thermo Fisher Scientific

Row Number	Average Mass	Intensity	Relative Abundance	Fractional Abundance	Score	Number of Charge States	Charge State Distribution	Mass Std Dev	PPM Std Dev	Delta Mass	Start Time (min)	Stop Time (min)	Apex RT
49	31526.34	88382.64	0.68	0.15	18.18	4	21 - 24	2.26	71.56	2929.76	16.000	22.000	17.660
50	26969.37	84586.95	0.65	0.14	21.52	4	15 - 18	1.66	61.72	-1627.21	16.000	22.000	17.530
51	30280.20	83680.91	0.64	0.14	32.96	8	16 - 23	2.42	79.90	1683.63	16.000	22.000	17.530
52	30002.83	81260.77	0.62	0.13	12.76	4	19 - 22	3.63	120.94	1406.26	16.000	22.000	17.640
53	29654.25	79330.51	0.61	0.13	13.68	4	27 - 30	3.73	125.74	1057.68	16.000	22.000	17.640
54	31406.71	78988.73	0.60	0.13	17.97	4	26 - 29	2.13	67.70	2810.14	16.000	22.000	17.660
55	34263.55	65561.31	0.50	0.11	19.56	4	22 - 25	3.54	103.32	5666.98	16.000	22.000	17.640
56	30323.39	65433.14	0.50	0.11	23.26	5	17 - 21	2.59	85.49	1726.82	16.000	22.000	17.880
57	27308.02	62208.41	0.48	0.10	19.56	4	22 - 25	1.45	53.11	-1288.55	16.000	22.000	17.640
58	25791.94	60523.04	0.46	0.10	11.41	4	17 - 20	2.23	86.53	-2804.63	16.000	22.000	17.260
59	31896.10	60412.41	0.46	0.10	16.67	4	19 - 22	2.92	91.60	3299.52	16.000	22.000	17.640
60	28670.74	56397.18	0.43	0.09	19.27	4	15 - 18	1.66	57.94	74.16	16.000	22.000	17.640
61	26240.33	53579.76	0.41	0.09	15.95	4	21 - 24	2.27	86.35	-2356.24	16.000	22.000	17.640
62	27274.85	51744.77	0.40	0.09	22.99	5	17 - 21	2.04	74.91	-1321.72	16.000	22.000	17.640
63	23879.11	47626.57	0.36	0.08	21.02	4	17 - 20	1.51	63.23	-4717.47	16.000	22.000	17.660
64	28748.77	46660.70	0.36	0.08	18.90	4	15 - 18	2.87	99.77	152.20	16.000	22.000	17.660
65	29843.26	44602.05	0.34	0.07	5.44	5	20 - 24	3.13	104.85	1246.68	16.000	22.000	17.500
66	28729.00	43929.03	0.34	0.07	21.75	4	15 - 18	1.73	60.25	132.42	16.000	22.000	17.830
67	32065.79	35675.66	0.27	0.06	8.31	4	18 - 21	1.10	34.30	3469.21	16.000	22.000	17.830
68	26389.98	18995.87	0.15	0.03	16.43	4	20 - 23	1.12	42.55	-2206.59	16.000	22.000	17.340

Copyright© 2011 - 2017 Thermo Fisher Scientific
