# Supplementary material for: Reprogramming of bacterial virulence by lysine acetylation
Source: Nat Commun. 2026 Apr 27;17:3859. doi: 10.1038/s41467-026-72244-8 (PMC13125535; doi:10.1038/s41467-026-72244-8)
Supplement: Supplementary file 5 — Supplementary Data 3 [file 41467_2026_72244_MOESM5_ESM.zip › Supplementary_Data_3/2_SnCE1_74-310_WT_SIRT1_treated_4713_02_4173_SUMUP_RE_01152026_154800.pdf]

BioPharma Finder Report

Created: 15/01/2026 15:48:55

Sample Information	
Raw File Name	D:\Data\4713\4713_02.raw
Instrument Method	C:\Xcalibur\methods\UltiMate\NoFAIMS_Intact_Protein\Direct_Injection_MS1_IT_7K_RF60_35min.meth
Vial	RA2
Injection Volume (µL)	1
Sample Weight	0
Sample Volume (µL)	0
ISTD Amount	0
Dil Factor	1

Chromatogram Parameters	
Use Restricted Time	True
Time Limits	15.000 - 24.984 minutes
Scan Range	558 - 930
m/z Range	600 - 2000
Chromatogram Trace Type	TIC
Sensitivity	High
Rel. Intensity Threshold (%)	5

Chromatogram

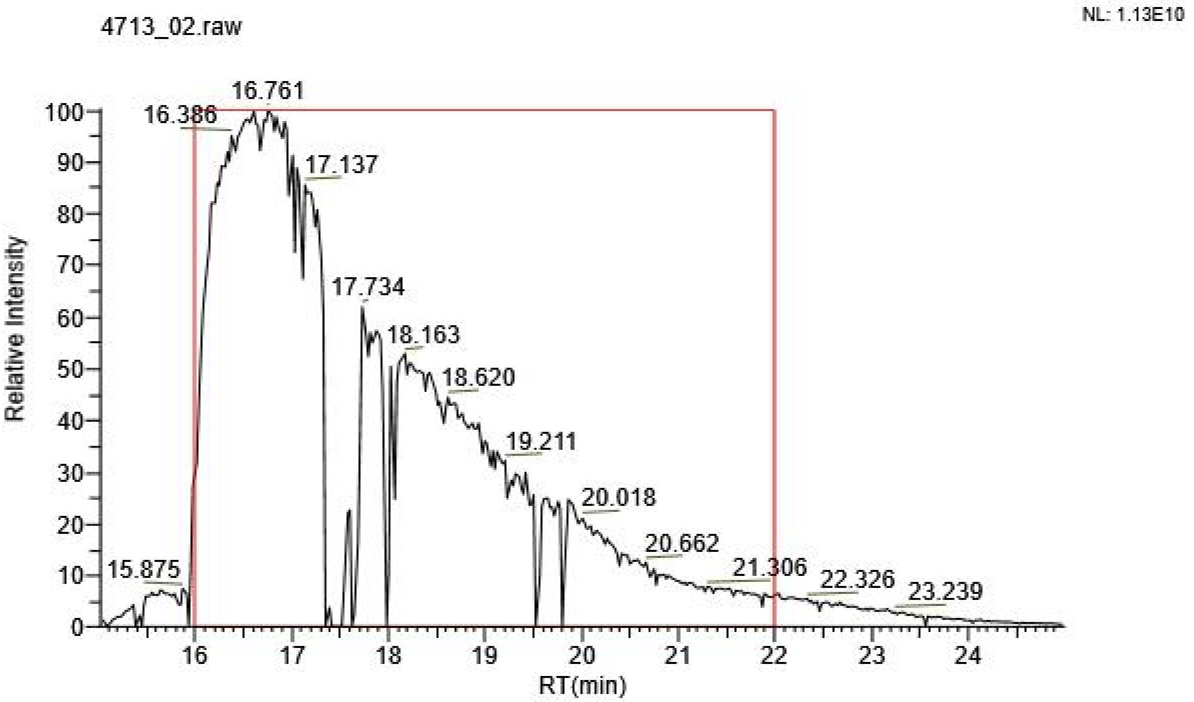

Copyright© 2011 - 2017 Thermo Fisher Scientific

01/15/2026 03:48:59

Main Parameters (ReSpect™)	
Deconvolution Results Filter	
Output Mass Range	22500 - 35000
Deconvoluted Spectra Display Mode	Isotopic Profile (new)
Charge State Distribution	
Deconvolution Mass Tolerance	30 ppm
Choice of Peak Model	
Choice of Peak Model	Intact Protein
Resolution at 400 m/z	
Raw File Specific	2000
Generate XIC for Each Component	
Calculate XIC	True

Advanced Parameters (ReSpect™)	
Charge State Distribution	
Model Mass Range	8000 - 70000
Charge State Range	7 - 100
Minimum Adjacent Charges (low & high model mass)	4 - 4
Noise Parameters	
Rel. Abundance Threshold (%)	0
Deconvolution Quality	
Quality Score Threshold	0
Choice of Peak Model	
Target Mass	28000 Da
Peak Model Parameters	
Number of Peak Models	1
Left/Right Peak Shape	2:2
Peak Filter Parameters	
Peak Detection Minimum Significance Measure	1 Standard Deviations
Peak Detection Quality Measure	95%
Specialized Parameters	
Peak Model Width Factor	1
Intensity Threshold Scale	0.01
Deconvolution Parameters	
Noise Compensation	True
Charge Carrier	H
Negative Charge	False

Source Spectra Parameters	
Source Spectra Method	Average Over Selected Retention Time
RT Range	16.000 - 22.000 minutes

Copyright© 2011 - 2017 Thermo Fisher Scientific

Source Spectrum

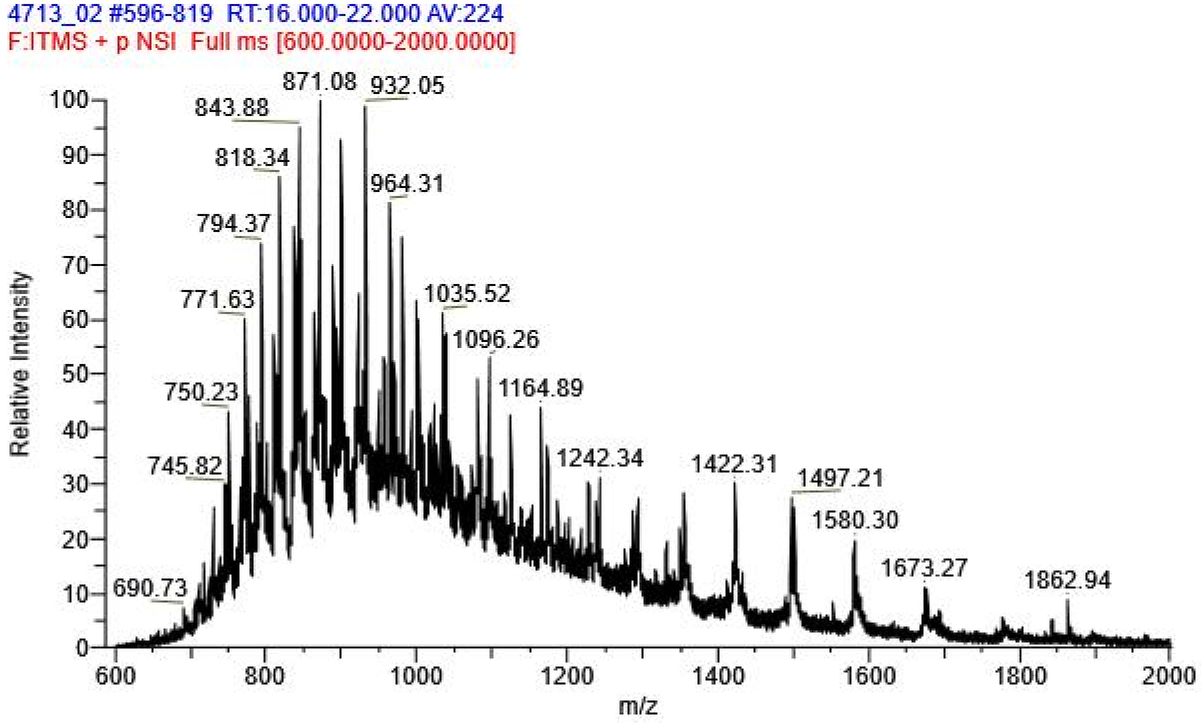

Copyright© 2011 - 2017 Thermo Fisher Scientific

01/15/2026 03:48:59

Deconvoluted Spectrum

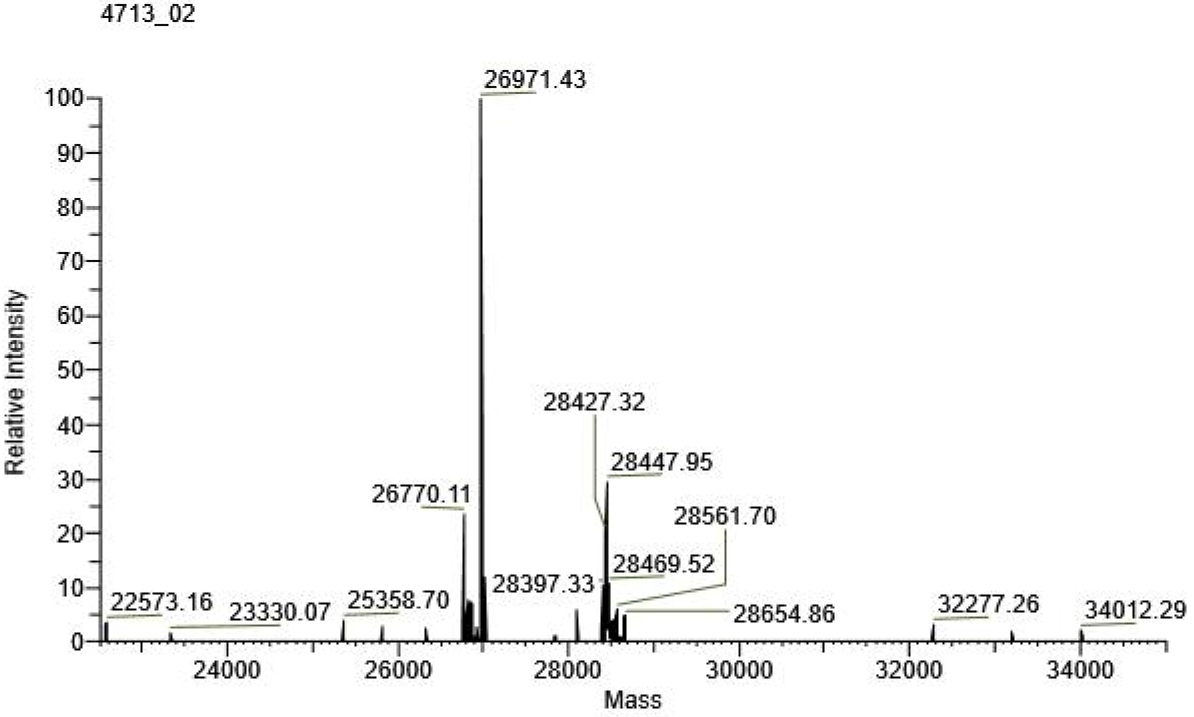

Copyright© 2011 - 2017 Thermo Fisher Scientific

01/15/2026 03:48:59

ReSpect Masses Table													
Row Number	Average Mass	Intensity	Relative Abundance	Fractional Abundance	Score	Number of Charge States	Charge State Distribution	Mass Std Dev	PPM Std Dev	Delta Mass	Start Time (min)	Stop Time (min)	Apex RT
1	26971.43	133468920.00	100.00	36.26	101.91	23	15 - 37	1.56	57.97	0.00	16.000	22.000	16.440
2	26770.11	31256142.00	23.42	8.49	64.18	14	18 - 31	1.20	44.81	-201.32	16.000	22.000	16.490
3	28447.95	31102232.00	23.30	8.45	31.57	6	32 - 37	2.02	71.11	1476.52	16.000	22.000	16.170
4	28427.32	26775334.00	20.06	7.27	49.36	11	15 - 25	1.28	44.95	1455.89	16.000	22.000	16.650
5	28469.52	14039190.00	10.52	3.81	37.59	9	15 - 23	1.42	49.93	1498.09	16.000	22.000	17.080
6	28397.33	12655980.00	9.48	3.44	22.56	4	33 - 36	2.62	92.36	1425.90	16.000	22.000	16.600
7	26813.98	10176112.00	7.62	2.76	22.05	4	28 - 31	2.07	77.17	-157.45	16.000	22.000	16.710
8	26856.70	9677330.00	7.25	2.63	22.53	4	29 - 32	1.75	65.22	-114.74	16.000	22.000	16.840
9	28449.56	8638319.00	6.47	2.35	46.60	11	15 - 25	2.01	70.52	1478.13	16.000	22.000	17.080
10	28561.70	7981136.00	5.98	2.17	18.49	4	34 - 37	2.45	85.79	1590.27	16.000	22.000	16.440
11	28093.10	7745065.00	5.80	2.10	19.47	4	25 - 28	2.97	105.56	1121.67	16.000	22.000	16.650
12	28654.86	6383514.00	4.78	1.73	20.25	4	33 - 36	2.18	76.06	1683.43	16.000	22.000	16.490
13	27013.53	5336413.50	4.00	1.45	18.70	4	25 - 28	2.43	89.86	42.10	16.000	22.000	16.730
14	28511.94	5230154.00	3.92	1.42	37.00	8	15 - 22	2.71	94.92	1540.50	16.000	22.000	17.140
15	25358.70	5147082.00	3.86	1.40	27.30	5	26 - 30	1.14	45.14	-1612.73	16.000	22.000	16.600
16	27014.66	4839013.50	3.63	1.31	20.03	4	20 - 23	1.50	55.58	43.23	16.000	22.000	16.730
17	27016.34	4810919.50	3.60	1.31	24.39	5	30 - 34	2.40	88.90	44.90	16.000	22.000	16.630
18	28491.97	4786161.50	3.59	1.30	31.62	7	16 - 22	1.20	42.13	1520.54	16.000	22.000	17.160
19	22573.16	4614498.00	3.46	1.25	18.75	4	20 - 23	2.48	109.99	-4398.28	16.000	22.000	17.080
20	32277.26	4123574.75	3.09	1.12	18.64	4	25 - 28	2.56	79.34	5305.83	16.000	22.000	16.170
21	25814.62	3694702.50	2.77	1.00	25.99	5	23 - 27	1.91	74.02	-1156.81	16.000	22.000	16.550
22	26918.36	3529515.75	2.64	0.96	17.95	4	29 - 32	2.87	106.68	-53.07	16.000	22.000	16.950
23	28404.02	3465701.75	2.60	0.94	32.79	7	16 - 22	1.55	54.48	1432.58	16.000	22.000	17.060
24	26319.38	3248579.00	2.43	0.88	19.13	4	32 - 35	2.03	77.30	-652.05	16.000	22.000	16.390
25	34012.29	2778874.75	2.08	0.75	29.84	6	24 - 29	2.69	79.13	7040.85	16.000	22.000	16.520
26	33195.64	2383654.00	1.79	0.65	18.00	4	41 - 44	2.35	70.88	6224.21	16.000	22.000	16.310
27	23330.07	2076965.13	1.56	0.56	15.75	4	18 - 21	2.86	122.75	-3641.36	16.000	22.000	17.000
28	27012.58	1562771.50	1.17	0.42	20.43	4	15 - 18	1.43	52.88	41.15	16.000	22.000	17.000
29	27841.74	1357866.88	1.02	0.37	19.01	4	24 - 27	1.11	40.01	870.30	16.000	22.000	16.490
30	26894.93	1344560.25	1.01	0.37	16.13	4	25 - 28	2.40	89.16	-76.50	16.000	22.000	16.390
31	28602.87	1247387.50	0.93	0.34	18.07	4	16 - 19	2.91	101.79	1631.44	16.000	22.000	17.190
32	28477.19	1149097.00	0.86	0.31	15.06	4	23 - 26	2.55	89.53	1505.76	16.000	22.000	16.140
33	28529.39	876734.50	0.66	0.24	25.26	5	15 - 19	2.98	104.29	1557.96	16.000	22.000	17.140
34	26947.49	612967.19	0.46	0.17	16.56	4	16 - 19	2.72	101.05	-23.94	16.000	22.000	17.080

Copyright© 2011 - 2017 Thermo Fisher Scientific
