# Supplementary material for: Reprogramming of bacterial virulence by lysine acetylation
Source: Nat Commun. 2026 Apr 27;17:3859. doi: 10.1038/s41467-026-72244-8 (PMC13125535; doi:10.1038/s41467-026-72244-8)
Supplement: Supplementary file 5 — Supplementary Data 3 [file 41467_2026_72244_MOESM5_ESM.zip › Supplementary_Data_3/25_SnCE1_83-310_WT_4713_25_4173_SUMUP_RE_01152026_154916.pdf]

BioPharma Finder Report

Created: 15/01/2026 16:02:08

Sample Information	
Raw File Name	D:\Data\4713\4713_25.raw
Instrument Method	C:\Xcalibur\methods\UltiMate\NoFAIMS_Intact_Protein\Direct_Injection_MS1_IT_7K_RF60_35min.meth
Vial	RC1
Injection Volume (µL)	1
Sample Weight	0
Sample Volume (µL)	0
ISTD Amount	0
Dil Factor	1

Chromatogram Parameters	
Use Restricted Time	True
Time Limits	15.000 - 24.984 minutes
Scan Range	558 - 930
m/z Range	600 - 2000
Chromatogram Trace Type	TIC
Sensitivity	High
Rel. Intensity Threshold (%)	5

Chromatogram

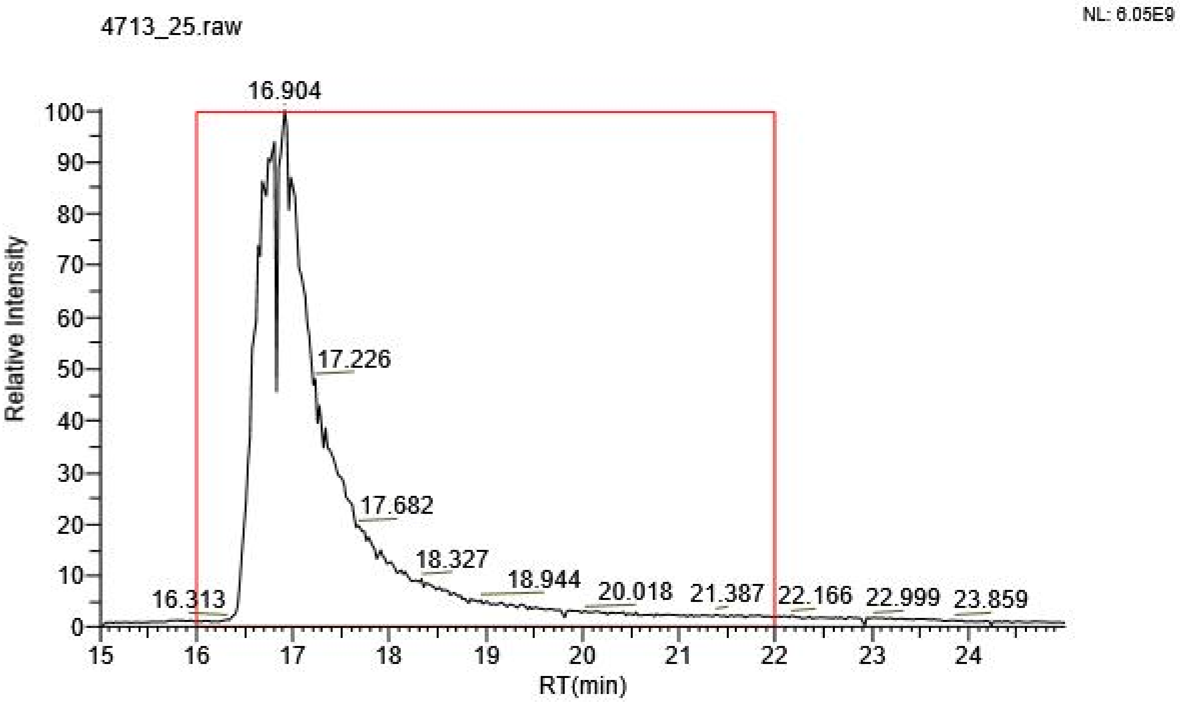

Copyright© 2011 - 2017 Thermo Fisher Scientific

Main Parameters (ReSpect™)	
Deconvolution Results Filter	
Output Mass Range	22500 - 35000
Deconvoluted Spectra Display Mode	Isotopic Profile (new)
Charge State Distribution	
Deconvolution Mass Tolerance	30 ppm
Choice of Peak Model	
Choice of Peak Model	Intact Protein
Resolution at 400 m/z	
Raw File Specific	2000
Generate XIC for Each Component	
Calculate XIC	True
**Advanced Parameters (ReSpect™)**	
Charge State Distribution	
Model Mass Range	8000 - 70000
Charge State Range	7 - 100
Minimum Adjacent Charges (low & high model mass)	4 - 4
Noise Parameters	
Rel. Abundance Threshold (%)	0
Deconvolution Quality	
Quality Score Threshold	0
Choice of Peak Model	
Target Mass	28000 Da
Peak Model Parameters	
Number of Peak Models	1
Left/Right Peak Shape	2:2
Peak Filter Parameters	
Peak Detection Minimum Significance Measure	1 Standard Deviations
Peak Detection Quality Measure	95%
Specialized Parameters	
Peak Model Width Factor	1
Intensity Threshold Scale	0.01
Deconvolution Parameters	
Noise Compensation	True
Charge Carrier	H
Negative Charge	False
**Source Spectra Parameters**	
Source Spectra Method	Average Over Selected Retention Time
RT Range	16.000 - 22.000 minutes

Copyright© 2011 - 2017 Thermo Fisher Scientific

Source Spectrum

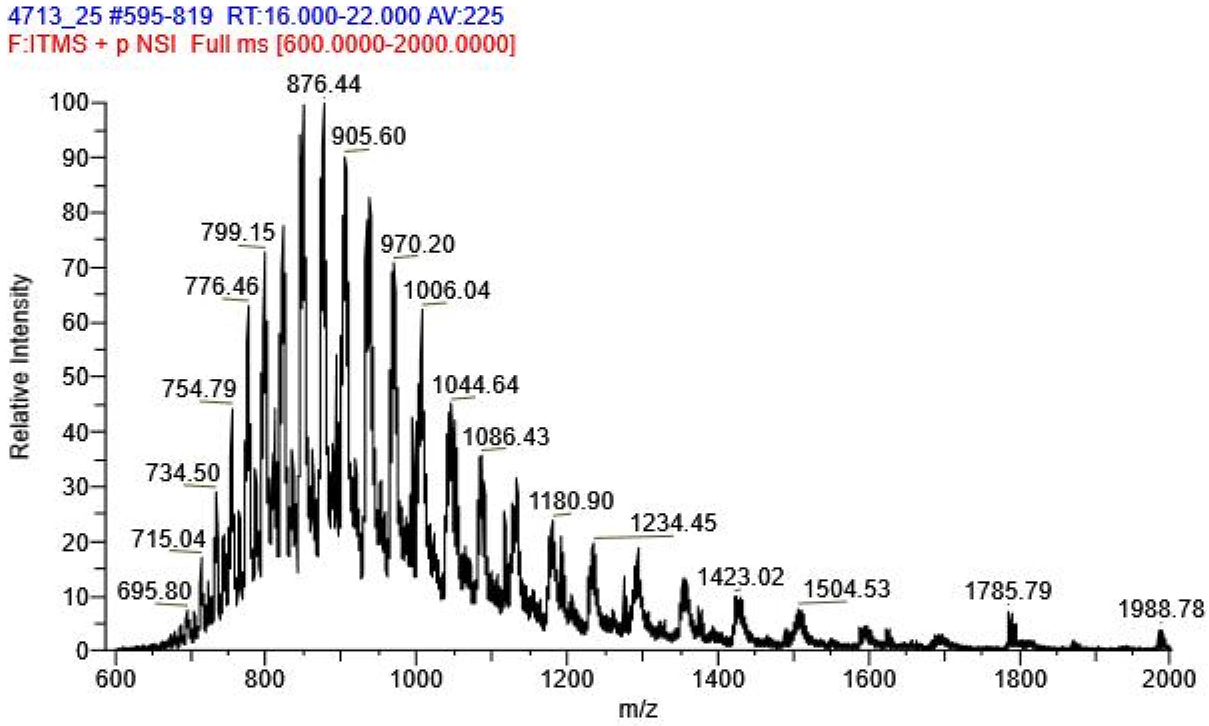

Copyright© 2011 - 2017 Thermo Fisher Scientific

01/15/2026 04:02:10

Deconvoluted Spectrum

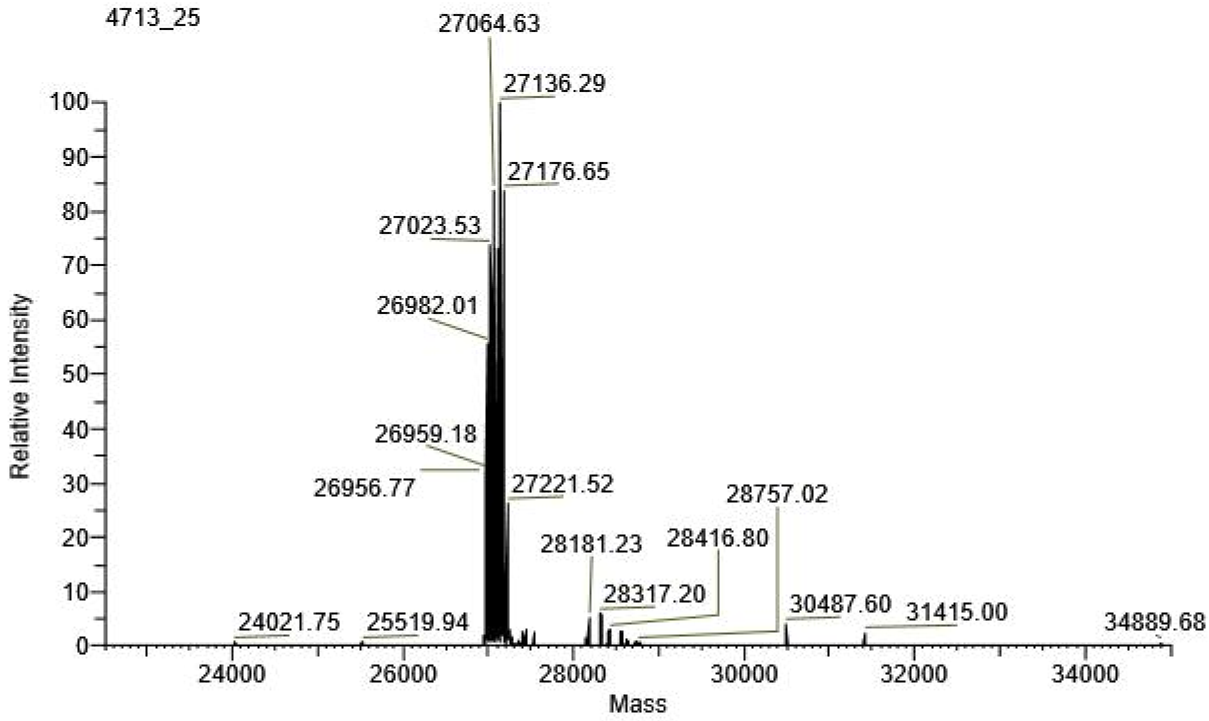

Copyright© 2011 - 2017 Thermo Fisher Scientific
01/15/2026 04:02:10

ReSpect Masses Table													
Row Number	Average Mass	Intensity	Relative Abundance	Fractional Abundance	Score	Number of Charge States	Charge State Distribution	Mass Std Dev	PPM Std Dev	Delta Mass	Start Time (min)	Stop Time (min)	Apex RT
1	27136.29	42282492.00	100.00	17.45	92.38	23	15 - 37	1.51	55.67	0.00	16.000	22.000	16.740
2	27176.65	31849522.00	75.33	13.15	63.67	13	26 - 38	1.74	63.85	40.36	16.000	22.000	16.900
3	27064.63	31774326.00	75.15	13.11	61.50	11	26 - 36	1.61	59.42	-71.66	16.000	22.000	16.800
4	27023.53	31142436.00	73.65	12.85	97.01	23	14 - 36	1.52	56.39	-112.76	16.000	22.000	16.690
5	27104.39	29037768.00	68.68	11.98	55.00	11	25 - 35	1.79	66.07	-31.90	16.000	22.000	16.930
6	26982.01	23451442.00	55.46	9.68	94.21	22	15 - 36	1.65	61.11	-154.28	16.000	22.000	16.640
7	27221.52	6304208.50	14.91	2.60	24.35	5	33 - 37	2.72	99.92	85.23	16.000	22.000	16.900
8	27063.19	3767765.50	8.91	1.56	38.96	9	15 - 23	1.90	70.13	-73.10	16.000	22.000	16.800
9	27177.73	3731165.00	8.82	1.54	35.91	8	16 - 23	1.10	40.32	41.45	16.000	22.000	16.900
10	27156.12	3626325.00	8.58	1.50	39.93	9	16 - 24	2.44	89.97	19.84	16.000	22.000	16.900
11	27223.96	3604363.75	8.52	1.49	16.37	4	25 - 28	1.85	67.95	87.67	16.000	22.000	16.900
12	28317.20	2533875.50	5.99	1.05	22.11	4	29 - 32	2.79	98.45	1180.91	16.000	22.000	16.930
13	27108.56	2327074.75	5.50	0.96	34.32	7	21 - 27	1.94	71.67	-27.72	16.000	22.000	16.930
14	28181.23	2147729.25	5.08	0.89	13.94	4	27 - 30	1.66	58.87	1044.95	16.000	22.000	16.800
15	27089.65	1864837.75	4.41	0.77	15.15	4	23 - 26	1.74	64.34	-46.64	16.000	22.000	16.900
16	30487.60	1675515.88	3.96	0.69	22.23	4	28 - 31	2.19	71.97	3351.32	16.000	22.000	16.980
17	27220.30	1624244.75	3.84	0.67	37.96	8	15 - 22	0.81	29.75	84.01	16.000	22.000	16.900
18	27043.77	1379269.75	3.26	0.57	25.58	6	17 - 22	1.94	71.73	-92.52	16.000	22.000	16.800
19	27199.94	1271095.38	3.01	0.52	37.84	9	14 - 22	1.89	69.53	63.66	16.000	22.000	16.900
20	28416.80	1253411.75	2.96	0.52	19.22	4	25 - 28	1.76	62.07	1280.52	16.000	22.000	16.930
21	27440.11	1249419.63	2.95	0.52	19.53	4	27 - 30	2.56	93.30	303.82	16.000	22.000	17.010
22	27003.26	1238379.50	2.93	0.51	40.09	9	15 - 23	1.56	57.88	-133.03	16.000	22.000	16.690
23	27242.67	1207819.63	2.86	0.50	28.17	7	16 - 22	2.00	73.50	106.38	16.000	22.000	16.900
24	28558.27	1075457.75	2.54	0.44	18.35	4	21 - 24	1.99	69.56	1421.98	16.000	22.000	16.800
25	27397.13	1070557.88	2.53	0.44	21.31	4	23 - 26	2.36	86.25	260.84	16.000	22.000	16.930
26	27538.55	1028651.75	2.43	0.42	18.05	4	33 - 36	2.16	78.38	402.27	16.000	22.000	17.170
27	27116.44	999220.13	2.36	0.41	24.87	6	15 - 20	1.20	44.14	-19.85	16.000	22.000	16.930
28	26959.18	959480.81	2.27	0.40	49.15	10	15 - 24	1.55	57.67	-177.11	16.000	22.000	16.640
29	31415.00	955412.81	2.26	0.39	21.81	4	30 - 33	1.90	60.33	4278.71	16.000	22.000	16.900
30	27280.54	676125.56	1.60	0.28	37.96	7	15 - 21	1.86	68.12	144.25	16.000	22.000	16.900
31	27100.73	638522.06	1.51	0.26	24.76	5	15 - 19	1.84	67.95	-35.56	16.000	22.000	16.880
32	28148.18	581516.88	1.38	0.24	16.40	5	25 - 29	2.63	93.49	1011.89	16.000	22.000	16.800
33	27259.81	511451.47	1.21	0.21	37.99	7	15 - 21	1.81	66.58	123.52	16.000	22.000	17.010
34	26956.77	510389.22	1.21	0.21	19.27	4	26 - 29	2.33	86.55	-179.52	16.000	22.000	16.640
35	28625.01	489832.13	1.16	0.20	20.01	4	21 - 24	1.29	45.21	1488.72	16.000	22.000	16.980
36	27353.16	384981.78	0.91	0.16	21.85	6	17 - 22	2.70	98.66	216.88	16.000	22.000	16.930
37	28732.03	322345.00	0.76	0.13	17.84	4	18 - 21	3.17	110.30	1595.75	16.000	22.000	16.880
38	24021.75	293949.97	0.70	0.12	16.87	4	21 - 24	1.55	64.45	-3114.54	16.000	22.000	16.690
39	25519.94	282513.75	0.67	0.12	24.08	5	15 - 19	1.55	60.73	-1616.35	16.000	22.000	16.690
40	28776.85	225184.39	0.53	0.09	19.42	5	16 - 20	2.37	82.35	1640.57	16.000	22.000	16.980
41	28712.21	208979.25	0.49	0.09	21.37	4	16 - 19	1.75	61.04	1575.92	16.000	22.000	16.850
42	28757.02	204458.92	0.48	0.08	20.02	4	19 - 22	2.47	85.73	1620.74	16.000	22.000	16.930
43	34889.68	176629.02	0.42	0.07	11.06	4	36 - 39	1.97	56.52	7753.39	16.000	22.000	16.900
44	28609.46	138053.16	0.33	0.06	7.83	4	18 - 21	2.81	98.25	1473.17	16.000	22.000	16.800
45	27317.96	111299.65	0.26	0.05	8.41	4	16 - 19	2.04	74.56	181.68	16.000	22.000	17.010
46	28390.73	95888.41	0.23	0.04	5.18	4	23 - 26	2.56	90.08	1254.44	16.000	22.000	16.900

Copyright© 2011 - 2017 Thermo Fisher Scientific
