# Supplementary material for: Reprogramming of bacterial virulence by lysine acetylation
Source: Nat Commun. 2026 Apr 27;17:3859. doi: 10.1038/s41467-026-72244-8 (PMC13125535; doi:10.1038/s41467-026-72244-8)
Supplement: Supplementary file 5 — Supplementary Data 3 [file 41467_2026_72244_MOESM5_ESM.zip › Supplementary_Data_3/5_SnCE1_74-310_S208A_4713_05_4173_SUMUP_RE_01152026_154803.pdf]

BioPharma Finder Report

Created: 15/01/2026 15:50:48

Sample Information	
Raw File Name	D:\Data\4713\4713_05.raw
Instrument Method	C:\Xcalibur\methods\UltiMate\NoFAIMS_Intact_Protein\Direct_Injection_MS1_IT_7K_RF60_35min.meth
Vial	RA5
Injection Volume (µL)	1
Sample Weight	0
Sample Volume (µL)	0
ISTD Amount	0
Dil Factor	1

Chromatogram Parameters	
Use Restricted Time	True
Time Limits	15.000 - 24.984 minutes
Scan Range	558 - 930
m/z Range	600 - 2000
Chromatogram Trace Type	TIC
Sensitivity	High
Rel. Intensity Threshold (%)	5

Chromatogram

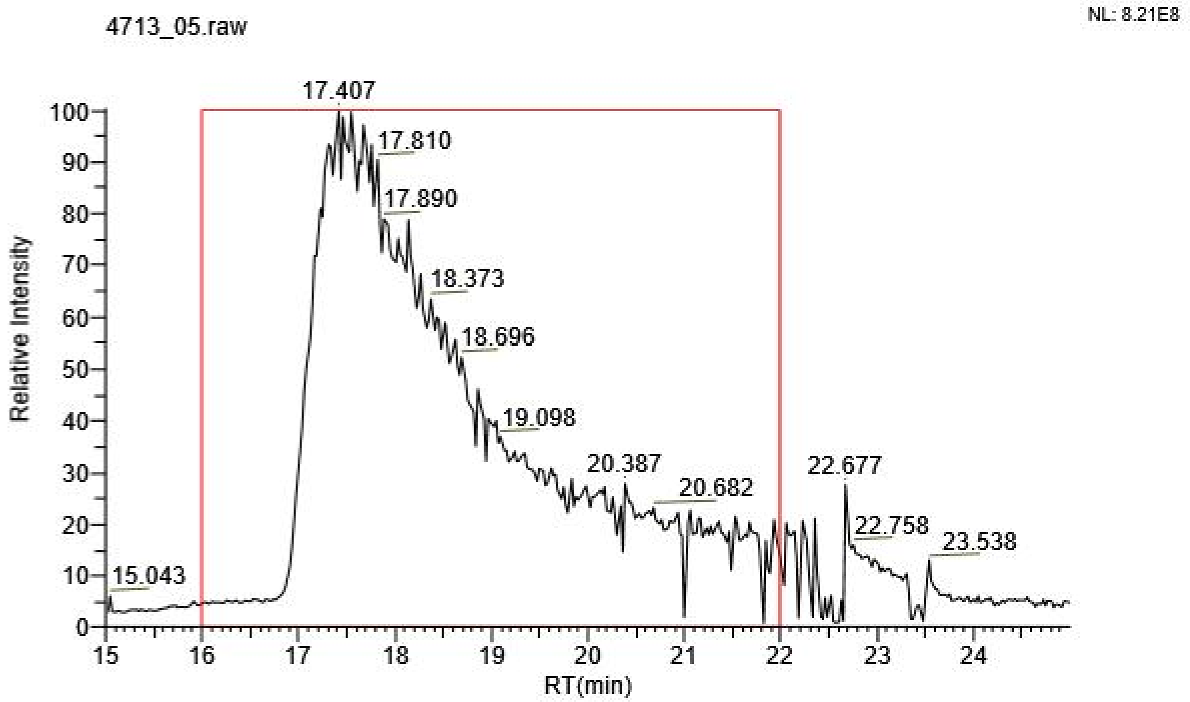

Copyright© 2011 - 2017 Thermo Fisher Scientific

Main Parameters (ReSpect™)	
Deconvolution Results Filter	
Output Mass Range	22500 - 35000
Deconvoluted Spectra Display Mode	Isotopic Profile (new)
Charge State Distribution	
Deconvolution Mass Tolerance	30 ppm
Choice of Peak Model	
Choice of Peak Model	Intact Protein
Resolution at 400 m/z	
Raw File Specific	2000
Generate XIC for Each Component	
Calculate XIC	True

Advanced Parameters (ReSpect™)	
Charge State Distribution	
Model Mass Range	8000 - 70000
Charge State Range	7 - 100
Minimum Adjacent Charges (low & high model mass)	4 - 4
Noise Parameters	
Rel. Abundance Threshold (%)	0
Deconvolution Quality	
Quality Score Threshold	0
Choice of Peak Model	
Target Mass	28000 Da
Peak Model Parameters	
Number of Peak Models	1
Left/Right Peak Shape	2:2
Peak Filter Parameters	
Peak Detection Minimum Significance Measure	1 Standard Deviations
Peak Detection Quality Measure	95%
Specialized Parameters	
Peak Model Width Factor	1
Intensity Threshold Scale	0.01
Deconvolution Parameters	
Noise Compensation	True
Charge Carrier	H
Negative Charge	False

Source Spectra Parameters	
Source Spectra Method	Average Over Selected Retention Time
RT Range	16.000 - 22.000 minutes

Copyright© 2011 - 2017 Thermo Fisher Scientific

Source Spectrum

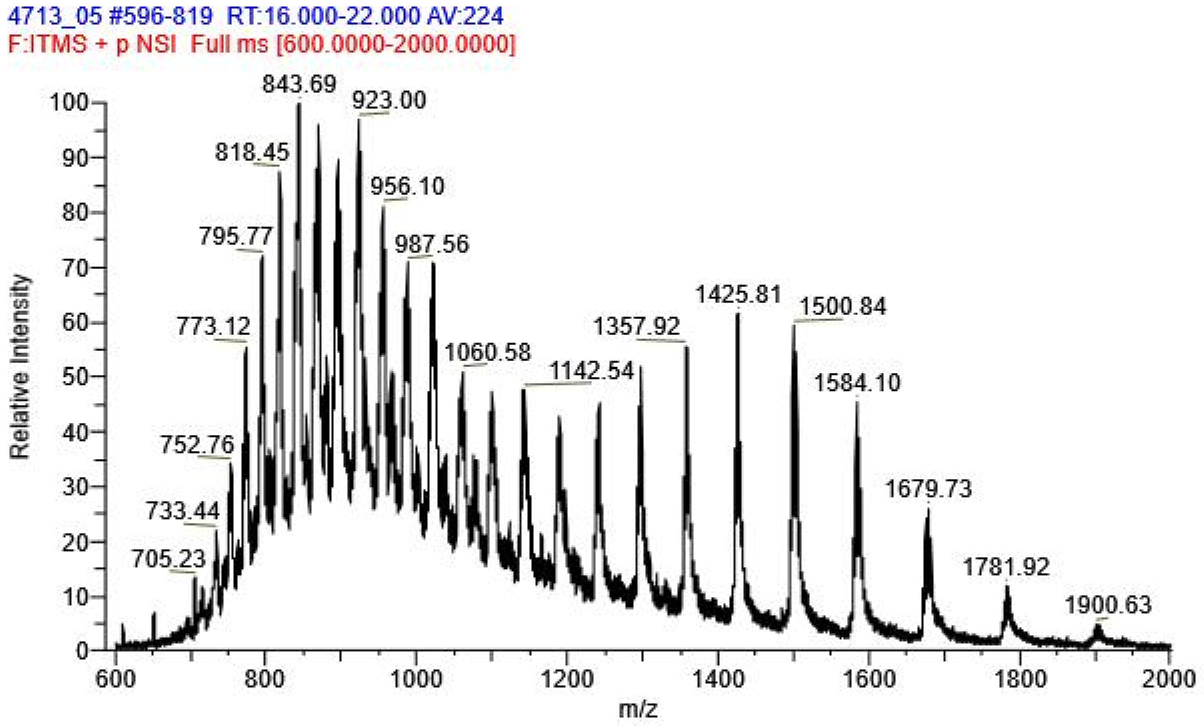

Copyright© 2011 - 2017 Thermo Fisher Scientific

01/15/2026 03:50:50

Deconvoluted Spectrum

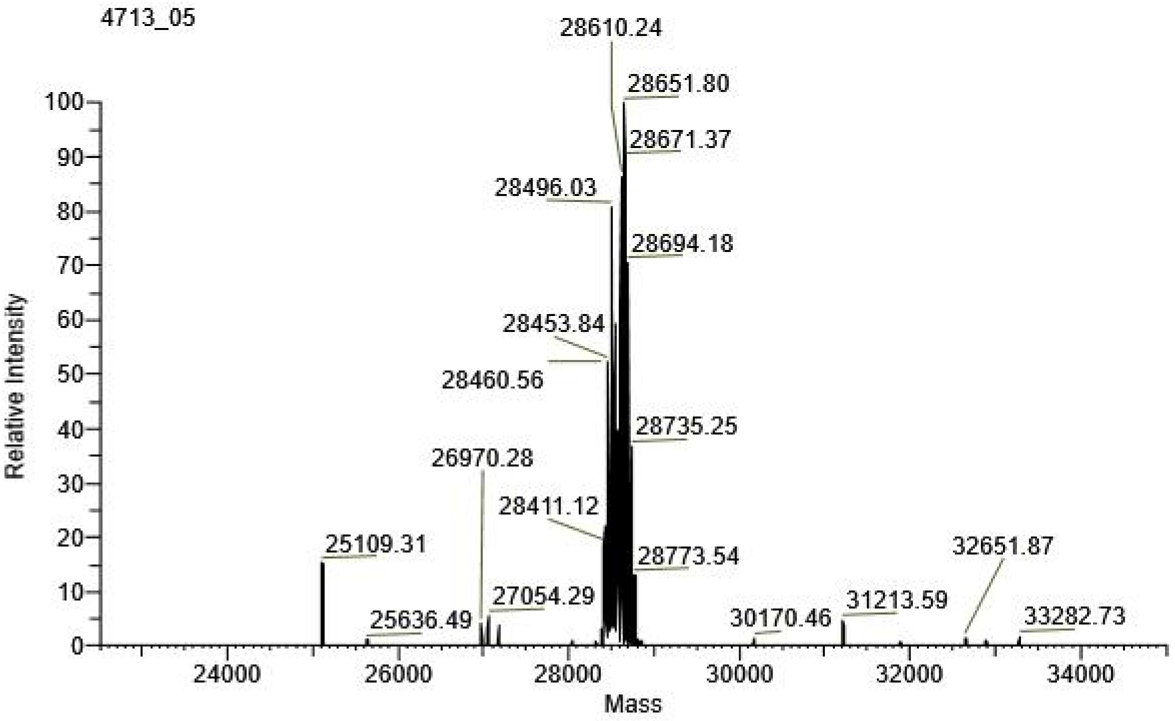

Copyright© 2011 - 2017 Thermo Fisher Scientific

01/15/2026 03:50:50

ReSpect Masses Table													
Row Number	Average Mass	Intensity	Relative Abundance	Fractional Abundance	Score	Number of Charge States	Charge State Distribution	Mass Std Dev	PPM Std Dev	Delta Mass	Start Time (min)	Stop Time (min)	Apex RT
1	28651.80	7931118.50	100.00	13.18	98.89	22	18 - 39	1.52	53.20	0.00	16.000	22.000	17.410
2	28610.24	6077178.50	76.62	10.10	58.39	11	29 - 39	1.56	54.58	-41.55	16.000	22.000	17.410
3	28694.18	5582210.50	70.38	9.27	109.42	24	15 - 38	2.29	79.82	42.38	16.000	22.000	17.460
4	28537.94	4688529.50	59.12	7.79	88.66	16	15 - 30	0.79	27.83	-113.85	16.000	22.000	17.760
5	28496.03	3889507.50	49.04	6.46	52.80	11	15 - 25	1.05	37.00	-155.77	16.000	22.000	17.810
6	28453.84	3391848.50	42.77	5.64	63.93	13	15 - 27	1.37	48.24	-197.96	16.000	22.000	17.700
7	28499.45	3003151.75	37.87	4.99	38.17	7	28 - 34	1.46	51.37	-152.34	16.000	22.000	17.300
8	28735.25	2828728.25	35.67	4.70	78.34	15	23 - 37	1.57	54.56	83.46	16.000	22.000	17.540
9	28580.21	2818517.25	35.54	4.68	67.47	13	15 - 27	0.94	33.02	-71.59	16.000	22.000	18.130
10	28567.74	2817482.25	35.52	4.68	37.11	9	32 - 40	2.58	90.34	-84.05	16.000	22.000	17.380
11	28460.56	1959874.00	24.71	3.26	32.65	6	29 - 34	1.71	59.94	-191.24	16.000	22.000	17.300
12	28624.31	1395049.63	17.59	2.32	55.39	12	15 - 26	2.76	96.33	-27.48	16.000	22.000	17.810
13	28475.07	1283690.25	16.19	2.13	51.57	11	15 - 25	0.93	32.64	-176.73	16.000	22.000	17.760
14	28605.44	1225882.00	15.46	2.04	56.76	12	15 - 26	2.28	79.67	-46.35	16.000	22.000	17.700
15	25109.31	1209393.88	15.25	2.01	22.69	4	27 - 30	0.79	31.33	-3542.49	16.000	22.000	17.460
16	28411.12	1164572.63	14.68	1.93	35.89	8	15 - 22	0.83	29.26	-240.68	16.000	22.000	17.540
17	28517.77	1150103.50	14.50	1.91	41.95	9	15 - 23	0.96	33.82	-134.03	16.000	22.000	17.810
18	28560.02	1065477.63	13.43	1.77	44.06	10	15 - 24	1.38	48.32	-91.78	16.000	22.000	17.810
19	28421.75	1060311.13	13.37	1.76	34.47	6	29 - 34	1.68	59.10	-230.05	16.000	22.000	17.300
20	28431.51	1027834.06	12.96	1.71	41.97	9	15 - 23	0.82	28.69	-220.28	16.000	22.000	17.540
21	28773.54	1026920.63	12.95	1.71	40.38	8	25 - 32	1.87	65.06	121.74	16.000	22.000	17.760
22	28418.63	559691.69	7.06	0.93	22.19	4	24 - 27	2.81	98.98	-233.17	16.000	22.000	17.650
23	27054.29	435033.94	5.49	0.72	23.47	4	27 - 30	1.69	62.49	-1597.51	16.000	22.000	17.490
24	31213.59	363095.66	4.58	0.60	17.60	4	33 - 36	2.73	87.38	2561.79	16.000	22.000	17.410
25	26970.28	324177.97	4.09	0.54	35.59	7	23 - 29	0.75	27.72	-1681.52	16.000	22.000	17.300
26	27178.67	295913.91	3.73	0.49	21.63	5	18 - 22	1.43	52.70	-1473.13	16.000	22.000	17.680
27	28671.37	280059.53	3.53	0.47	39.29	8	16 - 23	2.32	80.77	19.57	16.000	22.000	17.410
28	28387.70	245266.47	3.09	0.41	36.20	7	17 - 23	1.54	54.34	-264.10	16.000	22.000	17.410
29	33282.73	124953.04	1.58	0.21	17.74	4	29 - 32	1.83	54.84	4630.94	16.000	22.000	17.540
30	28718.18	120250.24	1.52	0.20	25.92	5	17 - 21	1.39	48.41	66.38	16.000	22.000	17.760
31	30170.46	105908.21	1.34	0.18	15.96	4	18 - 21	2.11	70.03	1518.67	16.000	22.000	17.730
32	32651.87	104674.59	1.32	0.17	15.90	4	32 - 35	2.00	61.27	4000.07	16.000	22.000	17.460
33	28804.45	93617.98	1.18	0.16	33.53	6	16 - 21	1.65	57.27	152.65	16.000	22.000	17.620
34	25636.49	85015.61	1.07	0.14	22.13	4	35 - 38	2.29	89.24	-3015.30	16.000	22.000	17.410
35	28735.66	78315.76	0.99	0.13	22.02	4	17 - 20	1.26	43.70	83.87	16.000	22.000	17.540
36	28039.18	72949.96	0.92	0.12	24.73	5	18 - 22	1.67	59.66	-612.62	16.000	22.000	19.260
37	32888.14	72442.42	0.91	0.12	20.24	4	27 - 30	2.67	81.10	4236.34	16.000	22.000	17.680
38	28846.07	63478.75	0.80	0.11	25.94	5	16 - 20	2.39	82.91	194.28	16.000	22.000	19.040
39	28311.98	62048.92	0.78	0.10	21.09	4	17 - 20	1.46	51.72	-339.82	16.000	22.000	17.380
40	27034.00	53695.49	0.68	0.09	20.74	4	17 - 20	2.35	87.09	-1617.80	16.000	22.000	17.810
41	31882.75	51827.67	0.65	0.09	19.31	4	20 - 23	3.62	113.61	3230.95	16.000	22.000	17.680

Copyright© 2011 - 2017 Thermo Fisher Scientific
