# Supplementary material for: Reprogramming of bacterial virulence by lysine acetylation
Source: Nat Commun. 2026 Apr 27;17:3859. doi: 10.1038/s41467-026-72244-8 (PMC13125535; doi:10.1038/s41467-026-72244-8)
Supplement: Supplementary file 5 — Supplementary Data 3 [file 41467_2026_72244_MOESM5_ESM.zip › Supplementary_Data_3/21_SnCE1_74-310_AcK209_C256A_4713_21_4713_mas_range_25k_30k_lc_range_8min_16min_12222025_164911.pdf]

BioPharma Finder Report

Created: 22/12/2025 16:50:43

Sample Information	
Raw File Name	D:\Data\4713\4713_21.raw
Instrument Method	C:\Xcalibur\methods\UltiMate\NoFAIMS_Intact_Protein\Direct_Injection_TD_Thermo_Settings_25min.meth
Vial	RF9
Injection Volume (µL)	1
Sample Weight	0
Sample Volume (µL)	0
ISTD Amount	0
Dil Factor	1

Chromatogram Parameters	
Use Restricted Time	True
Time Limits	8.000 - 16.000 minutes
Scan Range	227 - 617
m/z Range	400 - 2000
Chromatogram Trace Type	TIC
Sensitivity	High
Rel. Intensity Threshold (%)	5

Chromatogram

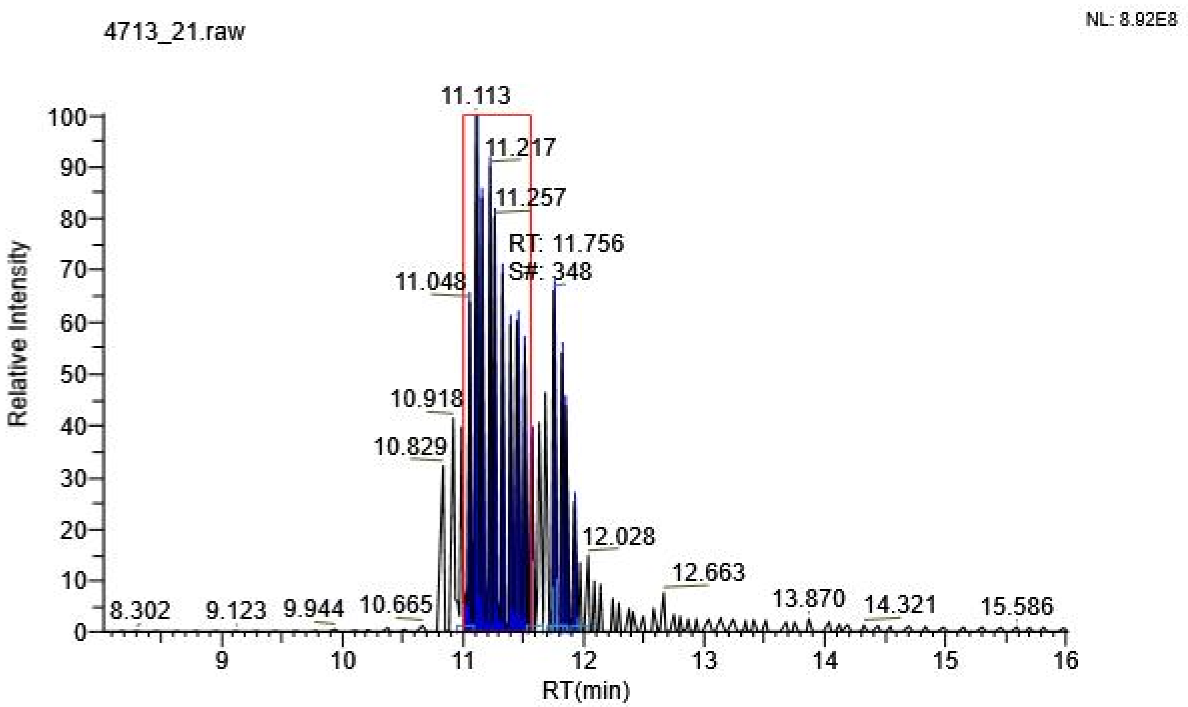

Copyright© 2011 - 2017 Thermo Fisher Scientific

12/22/2025 04:50:43

Main Parameters (ReSpect™)	
Deconvolution Results Filter	
Output Mass Range	25000 - 30000
Deconvoluted Spectra Display Mode	Isotopic Profile (new)
Charge State Distribution	
Deconvolution Mass Tolerance	50 ppm
Choice of Peak Model	
Choice of Peak Model	Intact Protein
Resolution at 400 m/z	
Raw File Specific	5303
Generate XIC for Each Component	
Calculate XIC	True

Advanced Parameters (ReSpect™)	
Charge State Distribution	
Model Mass Range	27000 - 30000
Charge State Range	10 - 50
Minimum Adjacent Charges (low & high model mass)	4 - 4
Noise Parameters	
Rel. Abundance Threshold (%)	5
Deconvolution Quality	
Quality Score Threshold	5
Choice of Peak Model	
Target Mass	28000 Da
Peak Model Parameters	
Number of Peak Models	1
Left/Right Peak Shape	2:2
Peak Filter Parameters	
Peak Detection Minimum Significance Measure	1 Standard Deviations
Peak Detection Quality Measure	95%
Specialized Parameters	
Peak Model Width Factor	1
Intensity Threshold Scale	0.01
Deconvolution Parameters	
Noise Compensation	True
Charge Carrier	H
Negative Charge	False

Source Spectra Parameters	
Source Spectra Method	Auto Peak Detection
Sensitivity	High
Rel. Intensity Threshold (%)	5

Copyright© 2011 - 2017 Thermo Fisher Scientific

Source Spectrum

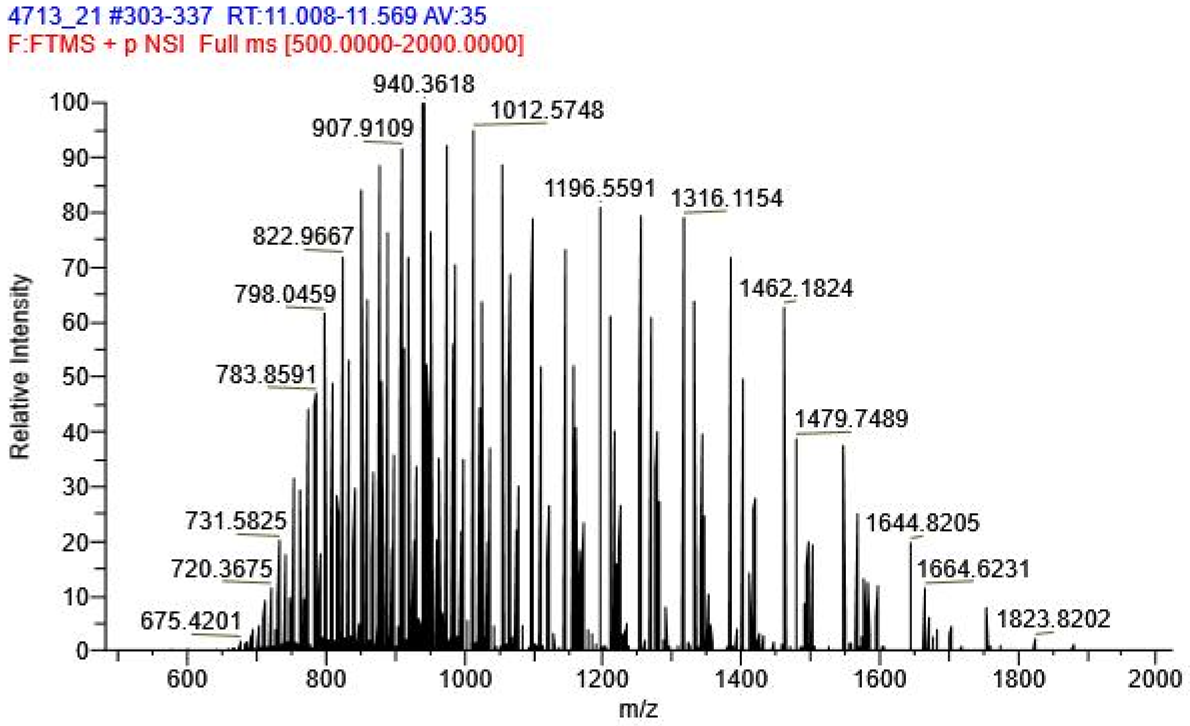

Copyright© 2011 - 2017 Thermo Fisher Scientific

12/22/2025 04:50:43

Deconvoluted Spectrum

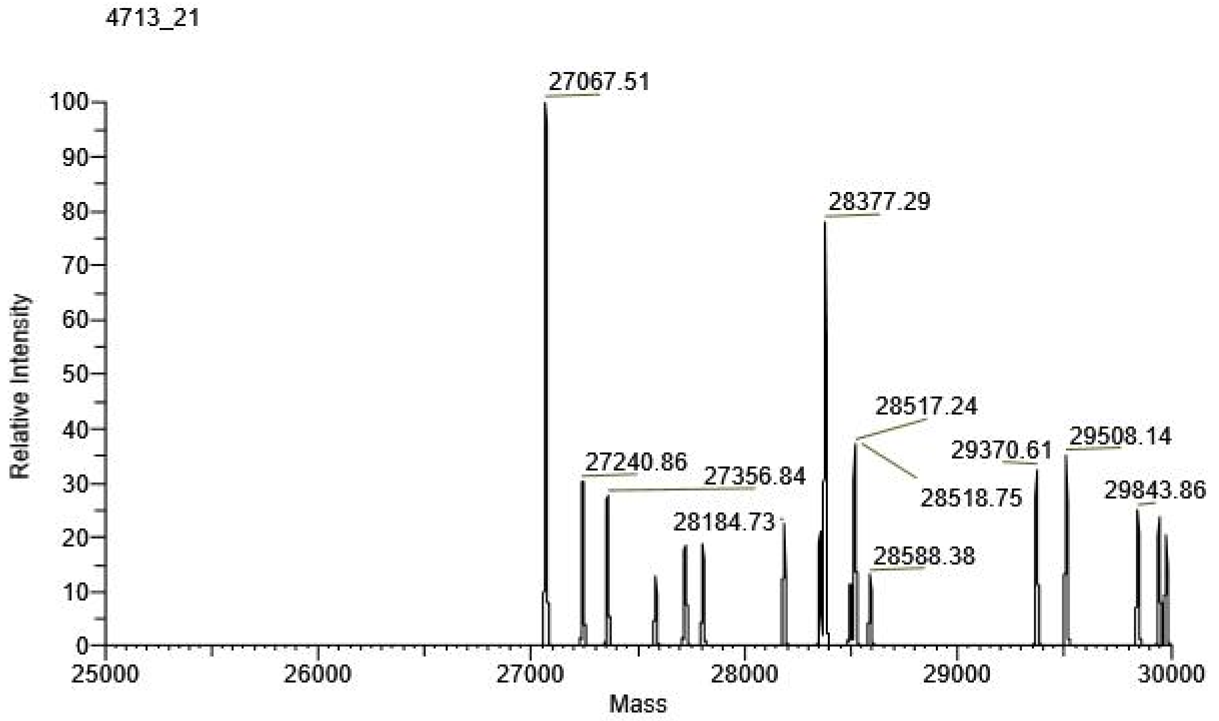

Copyright© 2011 - 2017 Thermo Fisher Scientific

ReSpect Masses Table														
Row Number	Average Mass	Intensity	Relative Abundance	Fractional Abundance	Score	Number of Charge States	Charge State Distribution	Mass Std Dev	PPM Std Dev	Delta Mass	Start Time (min)	Stop Time (min)	Apex RT
1	27067.51	54288232.00	100.00	18.78	94.03	17	19 - 35	1.42	52.56	0.00	11.008	11.569	11.320
2	28377.29	42264556.00	77.85	14.62	38.21	8	17 - 24	0.96	33.77	1309.79	11.008	11.569	11.570
3	29508.14	19021540.00	35.04	6.58	20.55	4	34 - 37	1.64	55.53	2440.63	11.008	11.569	11.220
4	29370.61	17555272.00	32.34	6.07	21.32	5	30 - 34	4.21	143.30	2303.10	11.008	11.569	11.110
5	27240.86	16386440.00	30.18	5.67	19.22	4	28 - 31	2.25	82.64	173.36	11.008	11.569	11.110
6	27356.84	14985313.00	27.60	5.18	21.29	5	33 - 37	1.74	63.68	289.34	11.008	11.569	11.220
7	29843.86	13548680.00	24.96	4.69	19.83	4	35 - 38	2.09	69.93	2776.35	11.008	11.569	11.220
8	29944.89	12858887.00	23.69	4.45	22.72	5	35 - 39	4.25	141.92	2877.38	11.008	11.569	11.220
9	28184.73	12204820.00	22.48	4.22	16.22	4	33 - 36	3.00	106.32	1117.22	11.008	11.569	11.220
10	28517.24	11931326.00	21.98	4.13	18.87	4	28 - 31	3.23	113.30	1449.73	11.008	11.569	11.220
11	28354.34	11420427.00	21.04	3.95	19.71	4	29 - 32	4.19	147.92	1286.83	11.008	11.569	11.500
12	29976.59	11058137.00	20.37	3.83	20.28	4	38 - 41	3.21	106.94	2909.08	11.008	11.569	11.110
13	27804.21	10145744.00	18.69	3.51	21.80	4	35 - 38	1.03	37.06	736.70	11.008	11.569	11.110
14	28518.75	8518047.00	15.69	2.95	22.84	7	33 - 39	4.66	163.52	1451.24	11.008	11.569	11.450
15	28588.38	7141165.00	13.15	2.47	17.47	4	34 - 37	4.67	163.47	1520.87	11.008	11.569	11.110
16	27719.42	6938665.00	12.78	2.40	19.77	4	32 - 35	2.46	88.66	651.92	11.008	11.569	11.390
17	27582.90	6933063.50	12.77	2.40	23.00	5	34 - 38	1.54	55.75	515.40	11.008	11.569	11.220
18	28495.16	6136015.00	11.30	2.12	18.56	4	37 - 40	3.87	135.87	1427.66	11.008	11.569	11.150
19	27725.55	5755959.00	10.60	1.99	16.51	4	36 - 39	2.60	93.73	658.04	11.008	11.569	11.110

Copyright© 2011 - 2017 Thermo Fisher Scientific

BioPharma Finder Report

Created: 22/12/2025 16:50:43

Sample Information	
Raw File Name	D:\Data\4713\4713_21.raw
Instrument Method	C:\Xcalibur\methods\UltiMate\NoFAIMS_Intact_Protein\Direct_Injection_TD_Thermo_Settings_25min.meth
Vial	RF9
Injection Volume (µL)	1
Sample Weight	0
Sample Volume (µL)	0
ISTD Amount	0
Dil Factor	1

Chromatogram Parameters	
Use Restricted Time	True
Time Limits	8.000 - 16.000 minutes
Scan Range	227 - 617
m/z Range	400 - 2000
Chromatogram Trace Type	TIC
Sensitivity	High
Rel. Intensity Threshold (%)	5

Chromatogram

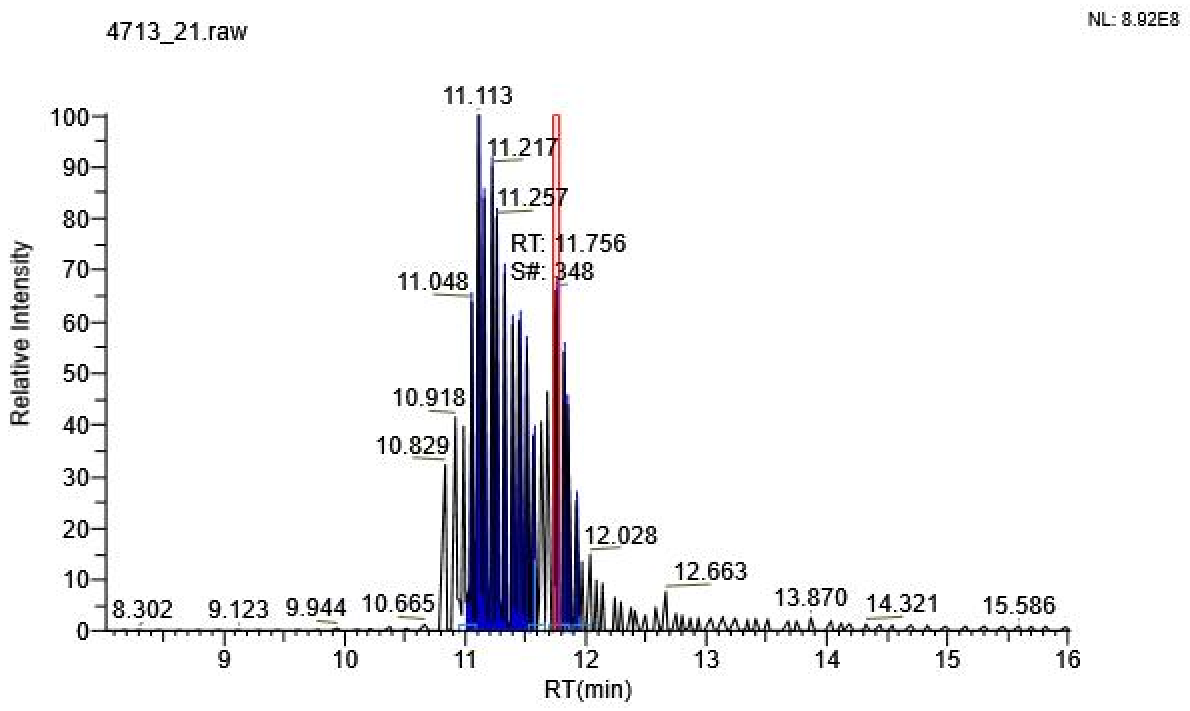

Copyright© 2011 - 2017 Thermo Fisher Scientific

Main Parameters (ReSpect™)	
Deconvolution Results Filter	
Output Mass Range	25000 - 30000
Deconvoluted Spectra Display Mode	Isotopic Profile (new)
Charge State Distribution	
Deconvolution Mass Tolerance	50 ppm
Choice of Peak Model	
Choice of Peak Model	Intact Protein
Resolution at 400 m/z	
Raw File Specific	5303
Generate XIC for Each Component	
Calculate XIC	True

Advanced Parameters (ReSpect™)	
Charge State Distribution	
Model Mass Range	27000 - 30000
Charge State Range	10 - 50
Minimum Adjacent Charges (low & high model mass)	4 - 4
Noise Parameters	
Rel. Abundance Threshold (%)	5
Deconvolution Quality	
Quality Score Threshold	5
Choice of Peak Model	
Target Mass	28000 Da
Peak Model Parameters	
Number of Peak Models	1
Left/Right Peak Shape	2:2
Peak Filter Parameters	
Peak Detection Minimum Significance Measure	1 Standard Deviations
Peak Detection Quality Measure	95%
Specialized Parameters	
Peak Model Width Factor	1
Intensity Threshold Scale	0.01
Deconvolution Parameters	
Noise Compensation	True
Charge Carrier	H
Negative Charge	False

Source Spectra Parameters	
Source Spectra Method	Auto Peak Detection
Sensitivity	High
Rel. Intensity Threshold (%)	5

Copyright© 2011 - 2017 Thermo Fisher Scientific

Source Spectrum

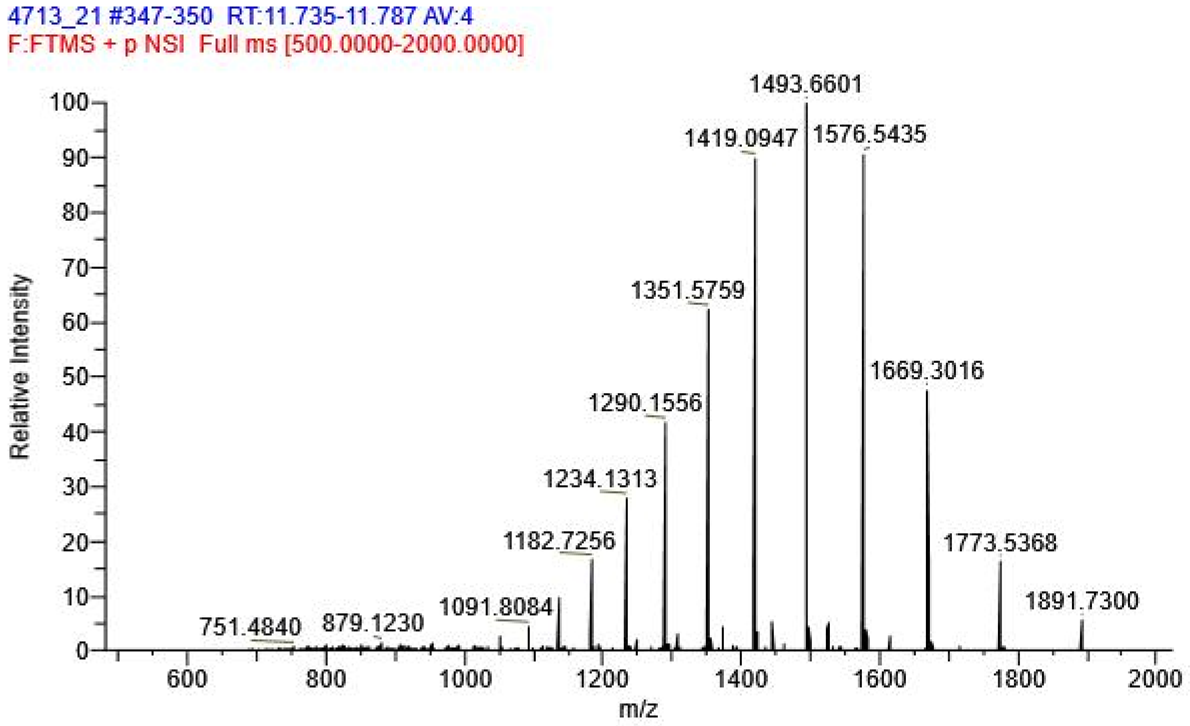

Copyright© 2011 - 2017 Thermo Fisher Scientific

12/22/2025 04:50:43

Deconvoluted Spectrum

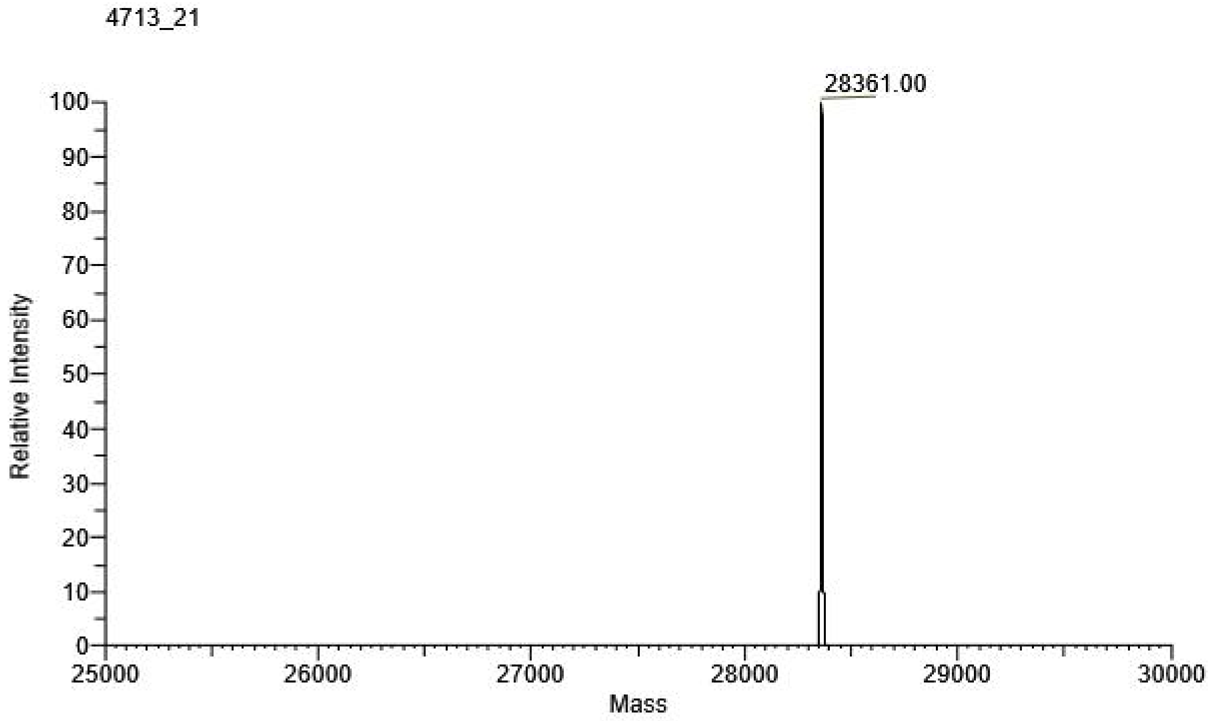

Copyright© 2011 - 2017 Thermo Fisher Scientific

ReSpect Masses Table														
Row Number	Average Mass	Intensity	Relative Abundance	Fractional Abundance	Score	Number of Charge States	Charge State Distribution	Mass Std Dev	PPM Std Dev	Delta Mass	Start Time (min)	Stop Time (min)	Apex RT
1	28361.00	2500542464.00	100.00	100.00	58.81	13	15 - 27	0.18	6.43	0.00	11.735	11.787	11.750

Copyright© 2011 - 2017 Thermo Fisher Scientific

BioPharma Finder Report

Created: 22/12/2025 16:50:44

Sample Information	
Raw File Name	D:\Data\4713\4713_21.raw
Instrument Method	C:\Xcalibur\methods\UltiMate\NoFAIMS_Intact_Protein\Direct_Injection_TD_Thermo_Settings_25min.meth
Vial	RF9
Injection Volume (µL)	1
Sample Weight	0
Sample Volume (µL)	0
ISTD Amount	0
Dil Factor	1

Chromatogram Parameters	
Use Restricted Time	True
Time Limits	8.000 - 16.000 minutes
Scan Range	227 - 617
m/z Range	400 - 2000
Chromatogram Trace Type	TIC
Sensitivity	High
Rel. Intensity Threshold (%)	5

Chromatogram

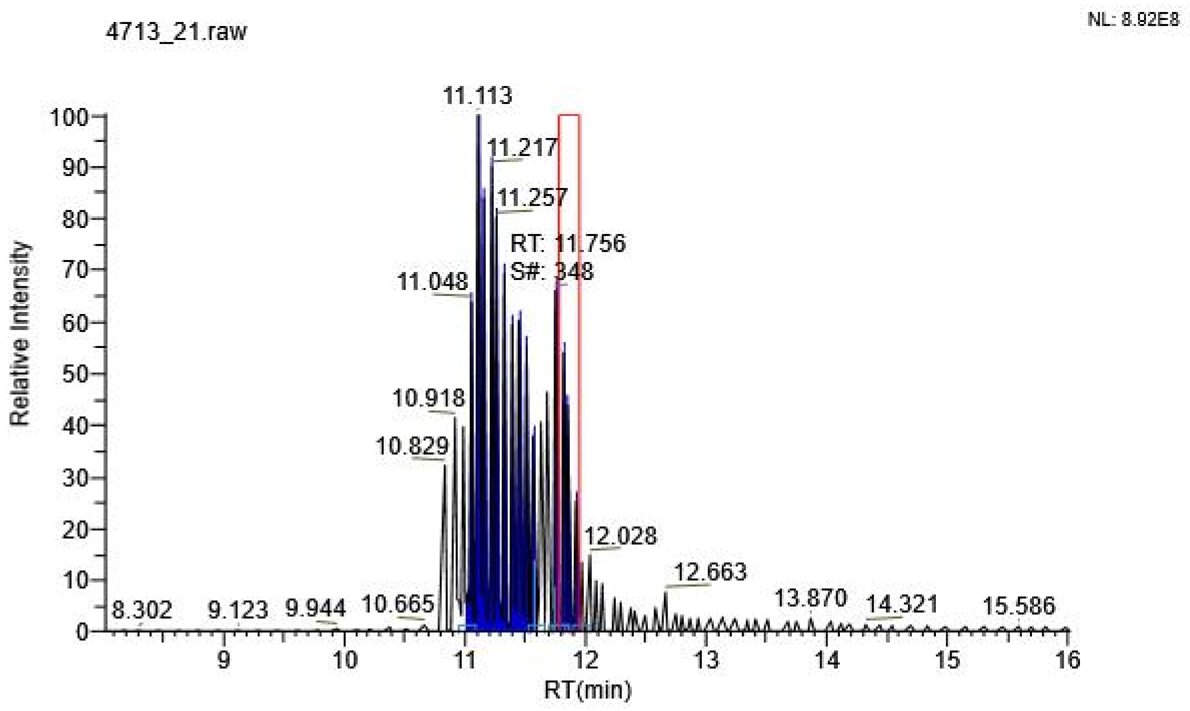

Copyright© 2011 - 2017 Thermo Fisher Scientific

Main Parameters (ReSpect™)	
Deconvolution Results Filter	
Output Mass Range	25000 - 30000
Deconvoluted Spectra Display Mode	Isotopic Profile (new)
Charge State Distribution	
Deconvolution Mass Tolerance	50 ppm
Choice of Peak Model	
Choice of Peak Model	Intact Protein
Resolution at 400 m/z	
Raw File Specific	5303
Generate XIC for Each Component	
Calculate XIC	True

Advanced Parameters (ReSpect™)	
Charge State Distribution	
Model Mass Range	27000 - 30000
Charge State Range	10 - 50
Minimum Adjacent Charges (low & high model mass)	4 - 4
Noise Parameters	
Rel. Abundance Threshold (%)	5
Deconvolution Quality	
Quality Score Threshold	5
Choice of Peak Model	
Target Mass	28000 Da
Peak Model Parameters	
Number of Peak Models	1
Left/Right Peak Shape	2:2
Peak Filter Parameters	
Peak Detection Minimum Significance Measure	1 Standard Deviations
Peak Detection Quality Measure	95%
Specialized Parameters	
Peak Model Width Factor	1
Intensity Threshold Scale	0.01
Deconvolution Parameters	
Noise Compensation	True
Charge Carrier	H
Negative Charge	False

Source Spectra Parameters	
Source Spectra Method	Auto Peak Detection
Sensitivity	High
Rel. Intensity Threshold (%)	5

Copyright© 2011 - 2017 Thermo Fisher Scientific

Source Spectrum

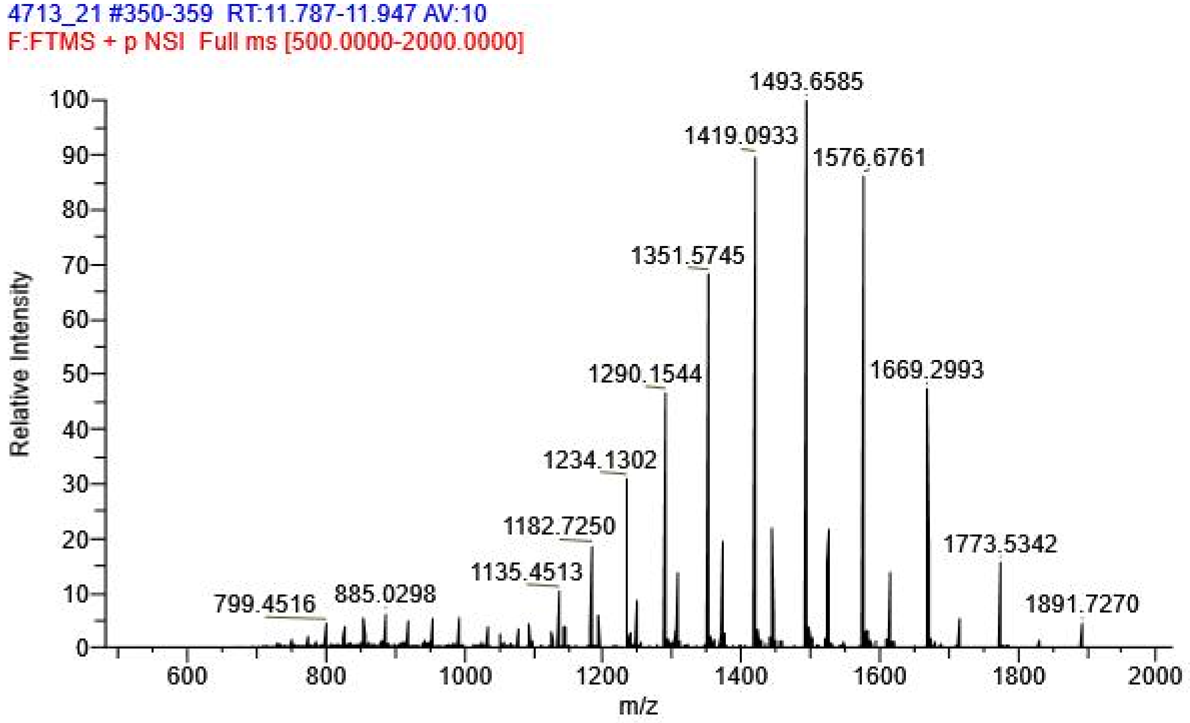

Copyright© 2011 - 2017 Thermo Fisher Scientific 12/22/2025 04:50:44

Deconvoluted Spectrum

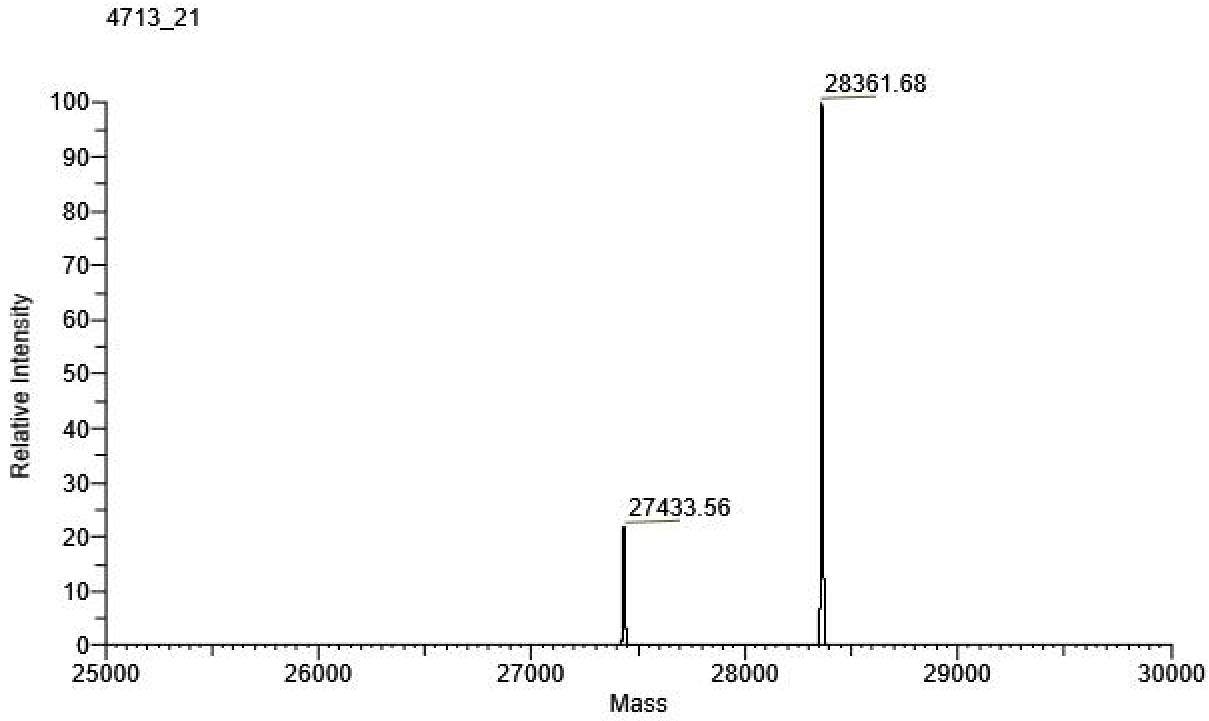

Copyright© 2011 - 2017 Thermo Fisher Scientific

ReSpect Masses Table														
Row Number	Average Mass	Intensity	Relative Abundance	Fractional Abundance	Score	Number of Charge States	Charge State Distribution	Mass Std Dev	PPM Std Dev	Delta Mass	Start Time (min)	Stop Time (min)	Apex RT
1	28361.68	1181931136.00	100.00	82.15	63.23	15	15 - 29	0.26	9.06	0.00	11.787	11.947	11.820
2	27433.56	256755408.00	21.72	17.85	47.62	12	15 - 26	0.24	8.62	-928.11	11.787	11.947	11.860

Copyright© 2011 - 2017 Thermo Fisher Scientific
