# Supplementary material for: Reprogramming of bacterial virulence by lysine acetylation
Source: Nat Commun. 2026 Apr 27;17:3859. doi: 10.1038/s41467-026-72244-8 (PMC13125535; doi:10.1038/s41467-026-72244-8)
Supplement: Supplementary file 5 — Supplementary Data 3 [file 41467_2026_72244_MOESM5_ESM.zip › Supplementary_Data_3/11_SnCE1_74-310_K247R_4713_11_4173_SUMUP_RE_01152026_154809.pdf]

BioPharma Finder Report

Created: 15/01/2026 15:54:21

Sample Information	
Raw File Name	D:\Data\4713\4713_11.raw
Instrument Method	C:\Xcalibur\methods\UltiMate\NoFAIMS_Intact_Protein\Direct_Injection_MS1_IT_7K_RF60_35min.meth
Vial	RA11
Injection Volume (µL)	1
Sample Weight	0
Sample Volume (µL)	0
ISTD Amount	0
Dil Factor	1

Chromatogram Parameters	
Use Restricted Time	True
Time Limits	15.000 - 24.984 minutes
Scan Range	558 - 930
m/z Range	600 - 2000
Chromatogram Trace Type	TIC
Sensitivity	High
Rel. Intensity Threshold (%)	5

Chromatogram

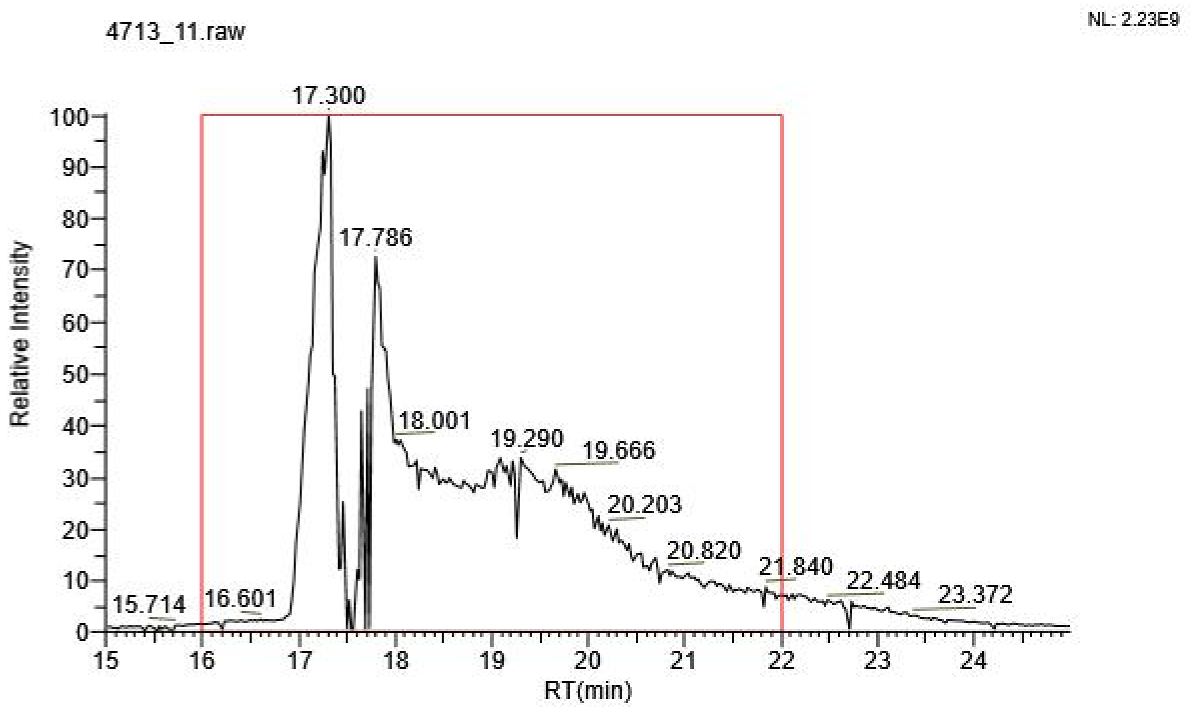

Copyright© 2011 - 2017 Thermo Fisher Scientific

01/15/2026 03:54:23

Main Parameters (ReSpect™)	
Deconvolution Results Filter	
Output Mass Range	22500 - 35000
Deconvoluted Spectra Display Mode	Isotopic Profile (new)
Charge State Distribution	
Deconvolution Mass Tolerance	30 ppm
Choice of Peak Model	
Choice of Peak Model	Intact Protein
Resolution at 400 m/z	
Raw File Specific	2000
Generate XIC for Each Component	
Calculate XIC	True
**Advanced Parameters (ReSpect™)**	
Charge State Distribution	
Model Mass Range	8000 - 70000
Charge State Range	7 - 100
Minimum Adjacent Charges (low & high model mass)	4 - 4
Noise Parameters	
Rel. Abundance Threshold (%)	0
Deconvolution Quality	
Quality Score Threshold	0
Choice of Peak Model	
Target Mass	28000 Da
Peak Model Parameters	
Number of Peak Models	1
Left/Right Peak Shape	2:2
Peak Filter Parameters	
Peak Detection Minimum Significance Measure	1 Standard Deviations
Peak Detection Quality Measure	95%
Specialized Parameters	
Peak Model Width Factor	1
Intensity Threshold Scale	0.01
Deconvolution Parameters	
Noise Compensation	True
Charge Carrier	H
Negative Charge	False
**Source Spectra Parameters**	
Source Spectra Method	Average Over Selected Retention Time
RT Range	16.000 - 22.000 minutes

Copyright© 2011 - 2017 Thermo Fisher Scientific

Source Spectrum

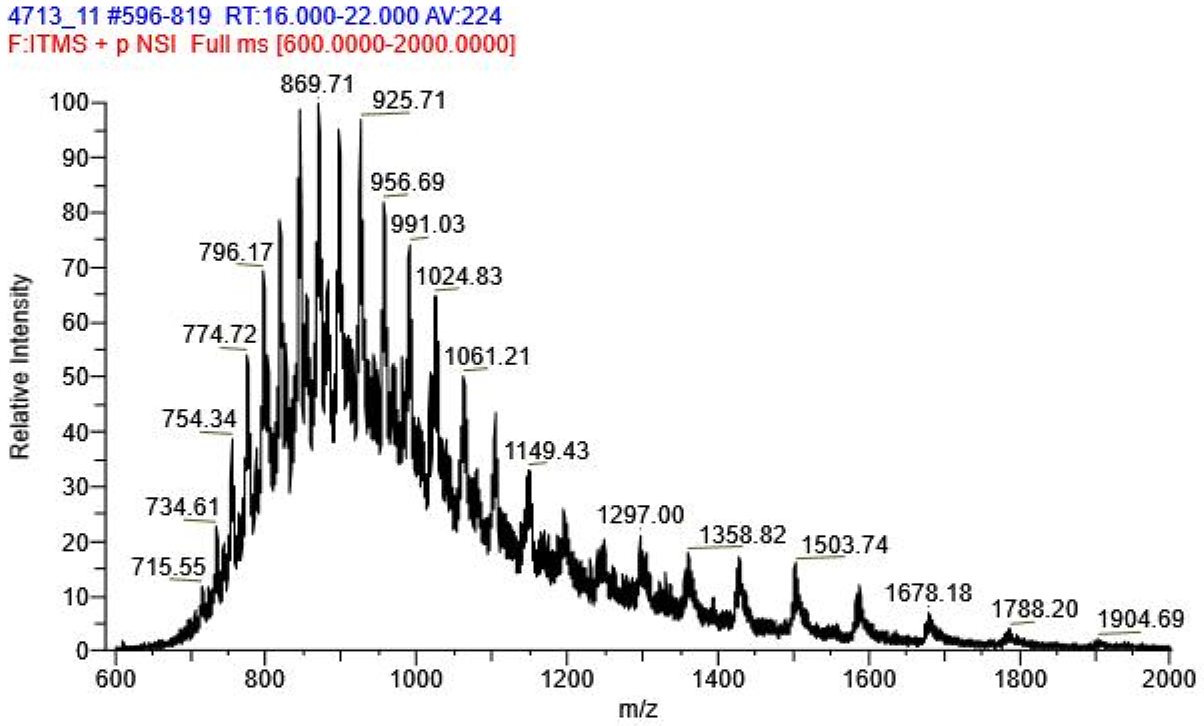

Copyright© 2011 - 2017 Thermo Fisher Scientific

Deconvoluted Spectrum

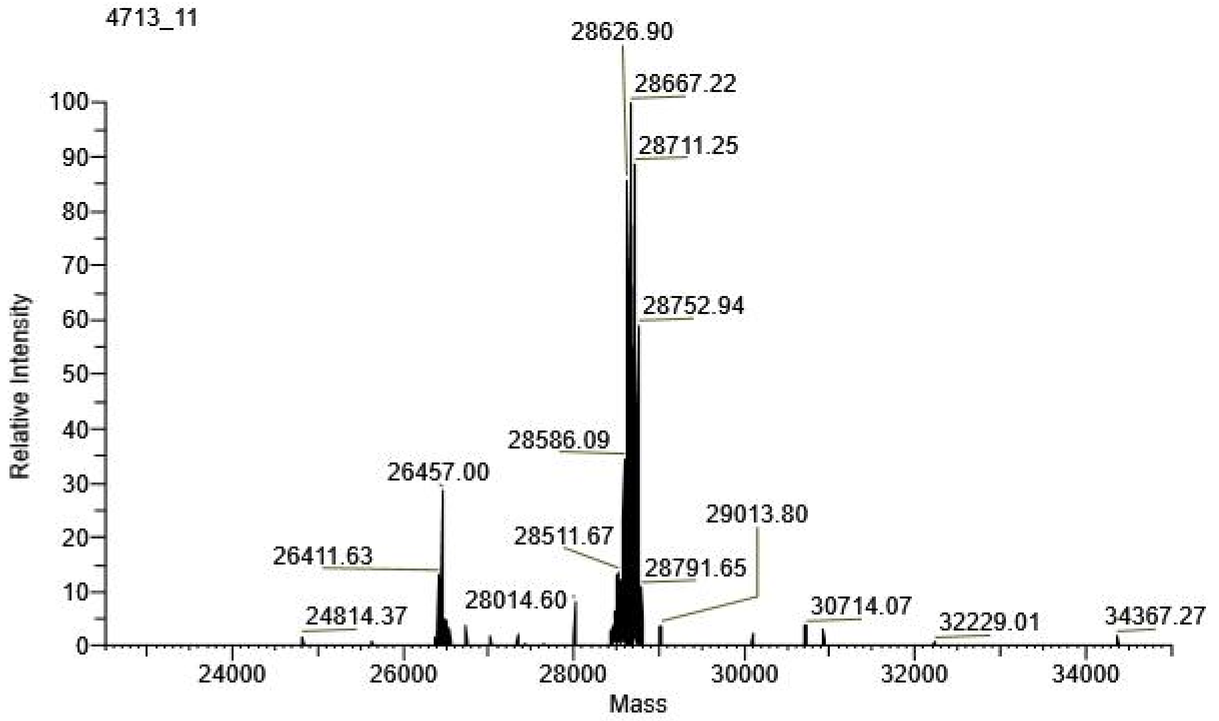

Copyright© 2011 - 2017 Thermo Fisher Scientific

01/15/2026 03:54:23

ReSpect Masses Table													
Row Number	Average Mass	Intensity	Relative Abundance	Fractional Abundance	Score	Number of Charge States	Charge State Distribution	Mass Std Dev	PPM Std Dev	Delta Mass	Start Time (min)	Stop Time (min)	Apex RT
1	28667.22	10569898.00	100.00	19.06	82.26	20	17 - 36	1.53	53.40	0.00	16.000	22.000	17.300
2	28711.25	8876480.00	83.98	16.00	62.64	12	25 - 36	1.91	66.54	44.03	16.000	22.000	17.300
3	28626.90	8644387.00	81.78	15.59	68.82	12	27 - 38	1.65	57.71	-40.32	16.000	22.000	17.250
4	28752.94	6211568.50	58.77	11.20	71.22	17	19 - 35	2.89	100.41	85.72	16.000	22.000	17.300
5	28586.09	3291018.25	31.14	5.93	46.72	9	28 - 36	1.72	60.06	-81.13	16.000	22.000	17.250
6	26457.00	2756526.75	26.08	4.97	54.44	10	26 - 35	1.48	56.00	-2210.22	16.000	22.000	17.300
7	28511.67	1416685.88	13.40	2.55	43.39	10	15 - 24	1.13	39.75	-155.55	16.000	22.000	17.840
8	26411.63	1380834.25	13.06	2.49	52.16	12	18 - 29	2.26	85.43	-2255.59	16.000	22.000	17.300
9	28554.27	1274815.25	12.06	2.30	44.77	10	15 - 24	1.45	50.67	-112.96	16.000	22.000	17.300
10	28791.65	1128832.50	10.68	2.04	34.17	6	27 - 32	2.16	75.15	124.43	16.000	22.000	17.790
11	28014.60	848268.44	8.03	1.53	21.25	4	34 - 37	2.19	78.28	-652.63	16.000	22.000	17.300
12	28596.04	696523.88	6.59	1.26	35.70	8	15 - 22	1.69	59.12	-71.18	16.000	22.000	17.330
13	28469.87	671193.63	6.35	1.21	33.71	8	15 - 22	1.70	59.57	-197.36	16.000	22.000	17.300
14	28709.12	533101.63	5.04	0.96	33.92	7	17 - 23	1.37	47.63	41.90	16.000	22.000	17.330
15	26497.51	510147.28	4.83	0.92	24.43	6	26 - 31	2.02	76.36	-2169.71	16.000	22.000	17.330
16	28623.56	500226.53	4.73	0.90	35.57	7	18 - 24	2.65	92.56	-43.66	16.000	22.000	17.250
17	28576.13	427578.69	4.05	0.77	31.08	7	16 - 22	2.76	96.69	-91.09	16.000	22.000	17.300
18	30714.07	391197.16	3.70	0.71	17.52	4	27 - 30	2.45	79.93	2046.85	16.000	22.000	17.330
19	26730.61	389689.41	3.69	0.70	20.41	4	33 - 36	1.86	69.65	-1936.61	16.000	22.000	17.300
20	28446.38	379507.09	3.59	0.68	33.50	7	17 - 23	1.71	60.20	-220.84	16.000	22.000	17.270
21	29013.80	374597.50	3.54	0.68	24.33	5	27 - 31	1.69	58.28	346.58	16.000	22.000	17.270
22	26539.52	338402.94	3.20	0.61	21.19	4	28 - 31	1.59	59.76	-2127.70	16.000	22.000	17.270
23	28533.28	331117.53	3.13	0.60	29.46	7	15 - 21	1.68	58.93	-133.95	16.000	22.000	17.330
24	30922.21	315144.28	2.98	0.57	17.07	4	29 - 32	2.28	73.73	2254.99	16.000	22.000	17.330
25	28639.35	314673.88	2.98	0.57	21.04	5	17 - 21	2.59	90.32	-27.87	16.000	22.000	17.330
26	26454.58	308597.53	2.92	0.56	19.53	4	19 - 22	2.36	89.03	-2212.64	16.000	22.000	17.330
27	28427.70	294043.75	2.78	0.53	34.20	7	16 - 22	1.20	42.18	-239.53	16.000	22.000	17.300
28	28585.04	253246.23	2.40	0.46	17.04	4	23 - 26	2.85	99.66	-82.18	16.000	22.000	17.250
29	28487.99	246553.33	2.33	0.44	31.25	7	16 - 22	1.35	47.48	-179.23	16.000	22.000	17.330
30	30097.63	234104.22	2.21	0.42	25.54	6	18 - 23	2.14	71.05	1430.41	16.000	22.000	17.300
31	27342.65	213674.56	2.02	0.39	16.15	4	19 - 22	1.44	52.67	-1324.57	16.000	22.000	17.330
32	34367.27	190544.58	1.80	0.34	19.08	4	45 - 48	3.38	98.46	5700.05	16.000	22.000	17.250
33	27012.17	184279.00	1.74	0.33	21.85	4	21 - 24	1.66	61.31	-1655.05	16.000	22.000	17.300
34	28687.59	182773.64	1.73	0.33	19.35	4	18 - 21	1.22	42.69	20.37	16.000	22.000	17.270
35	24814.37	164013.59	1.55	0.30	21.42	4	19 - 22	0.15	6.17	-3852.85	16.000	22.000	17.330
36	26372.38	162848.63	1.54	0.29	18.56	4	20 - 23	2.39	90.73	-2294.84	16.000	22.000	17.300
37	28780.51	130497.55	1.23	0.24	20.67	4	17 - 20	1.53	53.31	113.29	16.000	22.000	17.330
38	28732.85	110762.53	1.05	0.20	20.10	4	17 - 20	2.75	95.55	65.63	16.000	22.000	17.300
39	25623.92	83367.79	0.79	0.15	20.04	4	15 - 18	1.38	54.03	-3043.30	16.000	22.000	17.330
40	32229.01	74540.48	0.71	0.13	20.73	5	24 - 28	2.55	79.04	3561.79	16.000	22.000	17.250
41	27425.21	28946.52	0.27	0.05	10.59	4	24 - 27	3.10	113.06	-1242.01	16.000	22.000	17.300
42	27642.64	26040.48	0.25	0.05	5.73	5	24 - 28	2.35	85.17	-1024.58	16.000	22.000	17.300

Copyright© 2011 - 2017 Thermo Fisher Scientific
