# Supplementary material for: Reprogramming of bacterial virulence by lysine acetylation
Source: Nat Commun. 2026 Apr 27;17:3859. doi: 10.1038/s41467-026-72244-8 (PMC13125535; doi:10.1038/s41467-026-72244-8)
Supplement: Supplementary file 5 — Supplementary Data 3 [file 41467_2026_72244_MOESM5_ESM.zip › Supplementary_Data_3/1_SnCE1_74-310_WT_4713_01_4173_SUMUP_RE_01152026_154759.pdf]

BioPharma Finder Report

Created: 15/01/2026 15:48:21

Sample Information	
Raw File Name	D:\Data\4713\4713_01.raw
Instrument Method	C:\Xcalibur\methods\UltiMate\NoFAIMS_Intact_Protein\Direct_Injection_MS1_IT_7K_RF60_35min.meth
Vial	RA1
Injection Volume (µL)	1
Sample Weight	0
Sample Volume (µL)	0
ISTD Amount	0
Dil Factor	1

Chromatogram Parameters	
Use Restricted Time	True
Time Limits	15.000 - 24.984 minutes
Scan Range	558 - 930
m/z Range	600 - 2000
Chromatogram Trace Type	TIC
Sensitivity	High
Rel. Intensity Threshold (%)	5

Chromatogram

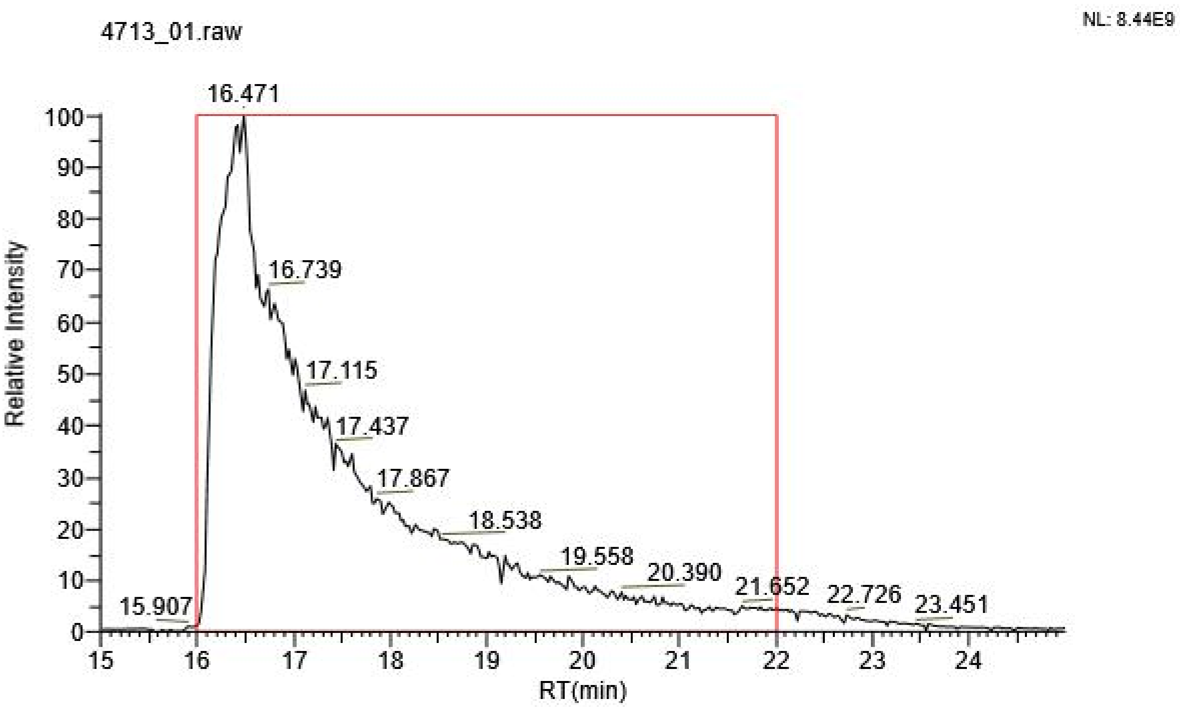

Copyright© 2011 - 2017 Thermo Fisher Scientific

Main Parameters (ReSpect™)	
Deconvolution Results Filter	
Output Mass Range	22500 - 35000
Deconvoluted Spectra Display Mode	Isotopic Profile (new)
Charge State Distribution	
Deconvolution Mass Tolerance	30 ppm
Choice of Peak Model	
Choice of Peak Model	Intact Protein
Resolution at 400 m/z	
Raw File Specific	2000
Generate XIC for Each Component	
Calculate XIC	True

Advanced Parameters (ReSpect™)	
Charge State Distribution	
Model Mass Range	8000 - 70000
Charge State Range	7 - 100
Minimum Adjacent Charges (low & high model mass)	4 - 4
Noise Parameters	
Rel. Abundance Threshold (%)	0
Deconvolution Quality	
Quality Score Threshold	0
Choice of Peak Model	
Target Mass	28000 Da
Peak Model Parameters	
Number of Peak Models	1
Left/Right Peak Shape	2:2
Peak Filter Parameters	
Peak Detection Minimum Significance Measure	1 Standard Deviations
Peak Detection Quality Measure	95%
Specialized Parameters	
Peak Model Width Factor	1
Intensity Threshold Scale	0.01
Deconvolution Parameters	
Noise Compensation	True
Charge Carrier	H
Negative Charge	False

Source Spectra Parameters	
Source Spectra Method	Average Over Selected Retention Time
RT Range	16.000 - 22.000 minutes

Copyright© 2011 - 2017 Thermo Fisher Scientific

Source Spectrum

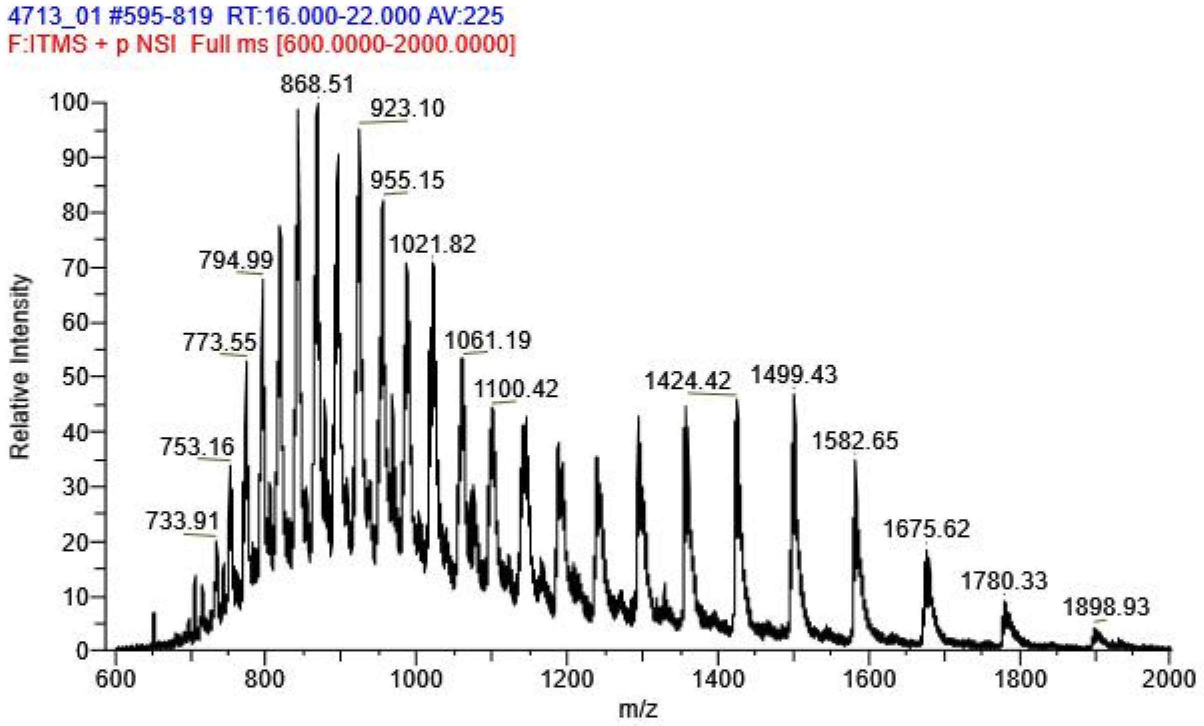

Copyright© 2011 - 2017 Thermo Fisher Scientific

01/15/2026 03:48:23

Deconvoluted Spectrum

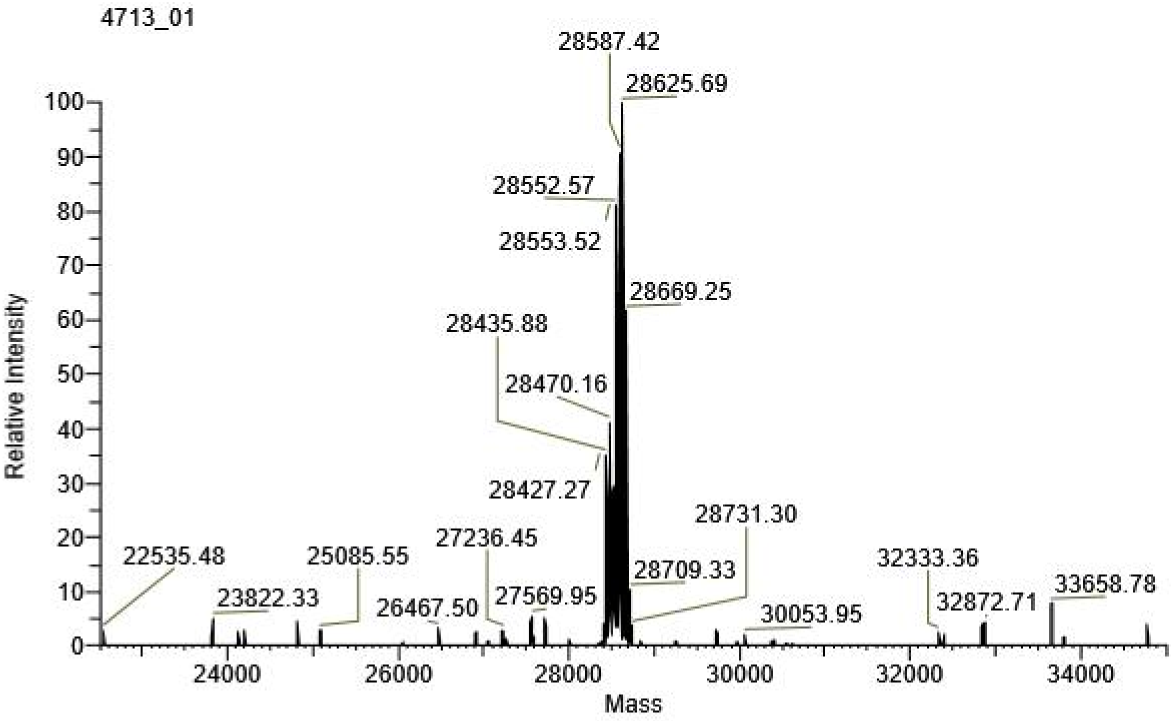

Copyright© 2011 - 2017 Thermo Fisher Scientific

01/15/2026 03:48:23

ReSpect Masses Table													
Row Number	Average Mass	Intensity	Relative Abundance	Fractional Abundance	Score	Number of Charge States	Charge State Distribution	Mass Std Dev	PPM Std Dev	Delta Mass	Start Time (min)	Stop Time (min)	Apex RT
1	28625.69	83032608.00	100.00	14.89	120.14	25	15 - 39	2.07	72.46	0.00	16.000	22.000	16.470
2	28587.42	69061256.00	83.17	12.39	66.11	13	28 - 40	1.34	46.74	-38.27	16.000	22.000	16.420
3	28552.57	50200824.00	60.46	9.00	68.23	13	25 - 37	2.05	71.77	-73.11	16.000	22.000	16.420
4	28669.25	42610752.00	51.32	7.64	53.17	9	28 - 36	1.65	57.41	43.56	16.000	22.000	16.470
5	28470.16	29390482.00	35.40	5.27	50.65	11	15 - 25	1.30	45.53	-155.53	16.000	22.000	16.790
6	28511.73	24316524.00	29.29	4.36	49.34	10	15 - 24	0.66	23.11	-113.96	16.000	22.000	16.390
7	28435.88	23691056.00	28.53	4.25	63.97	12	25 - 36	2.16	75.85	-189.80	16.000	22.000	16.180
8	28427.27	20388654.00	24.55	3.66	45.40	10	15 - 24	0.75	26.47	-198.42	16.000	22.000	16.710
9	28553.52	17653308.00	21.26	3.17	39.40	9	15 - 23	1.84	64.49	-72.17	16.000	22.000	16.470
10	28580.30	17430880.00	20.99	3.13	46.16	10	18 - 27	3.37	118.05	-45.39	16.000	22.000	16.390
11	28600.95	14858783.00	17.90	2.67	49.77	9	18 - 26	1.72	60.05	-24.73	16.000	22.000	16.500
12	28447.78	13567994.00	16.34	2.43	44.80	10	15 - 24	1.53	53.64	-177.91	16.000	22.000	16.500
13	28531.78	13514563.00	16.28	2.42	39.49	9	15 - 23	1.80	63.22	-93.91	16.000	22.000	16.820
14	28490.32	13440934.00	16.19	2.41	40.28	9	15 - 23	0.61	21.30	-135.37	16.000	22.000	16.500
15	28666.51	9862965.00	11.88	1.77	45.33	10	15 - 24	1.15	40.27	40.82	16.000	22.000	16.500
16	28475.63	9413069.00	11.34	1.69	27.02	5	32 - 36	1.31	45.86	-150.05	16.000	22.000	16.310
17	28645.05	9135039.00	11.00	1.64	40.85	9	16 - 24	1.41	49.36	19.36	16.000	22.000	16.420
18	33658.78	6377398.00	7.68	1.14	21.99	4	32 - 35	1.14	33.79	5033.09	16.000	22.000	16.420
19	28527.39	5128360.00	6.18	0.92	20.31	4	25 - 28	2.58	90.54	-98.29	16.000	22.000	16.280
20	28709.33	4463510.00	5.38	0.80	26.04	5	26 - 30	2.12	73.67	83.64	16.000	22.000	16.420
21	27569.95	4426063.00	5.33	0.79	30.71	7	22 - 28	1.97	71.49	-1055.74	16.000	22.000	16.470
22	27716.56	4186314.25	5.04	0.75	19.67	4	35 - 38	2.32	83.85	-909.13	16.000	22.000	16.390
23	23822.33	4120414.50	4.96	0.74	19.89	4	28 - 31	1.63	68.30	-4803.36	16.000	22.000	16.390
24	28709.45	4074531.75	4.91	0.73	27.83	5	19 - 23	1.20	41.76	83.76	16.000	22.000	16.390
25	24811.10	3705828.25	4.46	0.66	22.39	5	23 - 27	2.10	84.56	-3814.59	16.000	22.000	16.390
26	32872.71	3521917.25	4.24	0.63	14.42	4	31 - 34	2.77	84.34	4247.02	16.000	22.000	16.440
27	27548.70	3511923.50	4.23	0.63	28.21	6	21 - 26	2.66	96.74	-1076.99	16.000	22.000	16.470
28	32847.70	3452225.75	4.16	0.62	21.53	4	29 - 32	2.39	72.64	4222.01	16.000	22.000	16.470
29	28405.07	3358703.75	4.05	0.60	45.93	10	15 - 24	2.19	77.04	-220.62	16.000	22.000	16.630
30	34776.79	3238008.50	3.90	0.58	21.35	4	33 - 36	2.96	84.99	6151.10	16.000	22.000	16.470
31	28731.30	3112881.75	3.75	0.56	25.72	5	19 - 23	1.51	52.55	105.62	16.000	22.000	16.470
32	26467.50	2708125.00	3.26	0.49	19.14	4	23 - 26	2.08	78.69	-2158.18	16.000	22.000	16.420
33	28568.52	2392348.25	2.88	0.43	15.00	4	15 - 18	2.94	102.79	-57.17	16.000	22.000	16.740
34	29728.14	2388536.75	2.88	0.43	16.97	4	24 - 27	2.43	81.65	1102.45	16.000	22.000	16.440
35	25085.55	2340914.00	2.82	0.42	17.82	4	25 - 28	2.75	109.74	-3540.13	16.000	22.000	16.360
36	24190.20	2327228.25	2.80	0.42	17.84	4	26 - 29	1.37	56.61	-4435.49	16.000	22.000	16.390
37	22535.48	2267996.00	2.73	0.41	19.81	4	29 - 32	1.48	65.59	-6090.21	16.000	22.000	16.420
38	27215.98	2233565.50	2.69	0.40	39.19	8	17 - 24	2.06	75.87	-1409.71	16.000	22.000	16.500
39	24120.38	2100203.75	2.53	0.38	17.02	4	24 - 27	2.10	87.16	-4505.31	16.000	22.000	16.360
40	26908.01	2079845.75	2.50	0.37	21.29	4	15 - 18	1.80	67.04	-1717.67	16.000	22.000	16.710
41	28689.41	2053722.50	2.47	0.37	17.20	4	17 - 20	2.50	86.99	63.72	16.000	22.000	16.390
42	32333.36	2010572.25	2.42	0.36	17.36	4	36 - 39	2.47	76.31	3707.68	16.000	22.000	16.500
43	32399.96	1716485.88	2.07	0.31	23.34	5	32 - 36	2.16	66.60	3774.28	16.000	22.000	16.360
44	30053.95	1667851.00	2.01	0.30	14.90	4	19 - 22	2.70	89.85	1428.26	16.000	22.000	16.470
45	33804.26	1320844.13	1.59	0.24	15.34	4	26 - 29	2.45	72.56	5178.57	16.000	22.000	16.470
46	27258.46	1095047.50	1.32	0.20	20.12	4	18 - 21	1.33	48.88	-1367.23	16.000	22.000	16.500
47	27998.95	932577.81	1.12	0.17	18.31	4	25 - 28	3.03	108.21	-626.74	16.000	22.000	16.470
48	30400.21	788406.31	0.95	0.14	21.44	4	17 - 20	2.35	77.44	1774.52	16.000	22.000	16.420

Copyright© 2011 - 2017 Thermo Fisher Scientific

Row Number	Average Mass	Intensity	Relative Abundance	Fractional Abundance	Score	Number of Charge States	Charge State Distribution	Mass Std Dev	PPM Std Dev	Delta Mass	Start Time (min)	Stop Time (min)	Apex RT
49	30381.09	764952.13	0.92	0.14	18.86	4	17 - 20	2.87	94.47	1755.40	16.000	22.000	16.580
50	28835.89	689291.13	0.83	0.12	20.06	4	17 - 20	2.04	70.75	210.20	16.000	22.000	16.500
51	29240.39	680204.00	0.82	0.12	24.53	5	40 - 44	2.03	69.32	614.71	16.000	22.000	16.440
52	27050.38	630640.38	0.76	0.11	11.56	4	17 - 20	3.05	112.90	-1575.30	16.000	22.000	16.790
53	27236.45	605760.06	0.73	0.11	22.48	5	17 - 21	1.97	72.22	-1389.24	16.000	22.000	16.420
54	28378.23	597272.75	0.72	0.11	21.62	4	17 - 20	1.92	67.61	-247.46	16.000	22.000	16.500
55	29968.10	549243.81	0.66	0.10	15.74	4	38 - 41	2.23	74.35	1342.41	16.000	22.000	16.260
56	29264.49	521211.84	0.63	0.09	20.45	4	19 - 22	2.46	84.06	638.80	16.000	22.000	16.850
57	26051.09	507461.72	0.61	0.09	16.88	4	25 - 28	1.83	70.13	-2574.60	16.000	22.000	16.360
58	28352.18	455876.97	0.55	0.08	20.91	4	18 - 21	2.01	70.87	-273.51	16.000	22.000	16.310
59	30549.78	354568.22	0.43	0.06	20.95	4	16 - 19	0.75	24.43	1924.09	16.000	22.000	16.950
60	30612.28	283879.22	0.34	0.05	19.37	4	16 - 19	3.41	111.41	1986.59	16.000	22.000	16.500
61	26888.29	209377.44	0.25	0.04	3.79	4	14 - 17	2.18	81.21	-1737.39	16.000	22.000	16.790

Copyright© 2011 - 2017 Thermo Fisher Scientific
